# A New Method for Production of Chiral 2-Aryl-2-fluoropropanoic Acids Using an Effective Kinetic Resolution of Racemic 2-Aryl-2-fluoropropanoic Acids

**DOI:** 10.3390/molecules17067356

**Published:** 2012-06-14

**Authors:** Atsushi Tengeiji, Isamu Shiina

**Affiliations:** Department of Applied Chemistry, Faculty of Science, Tokyo University of Science, 1-3 Kagurazaka, Shinjuku-ku, Tokyo 162-8601, Japan

**Keywords:** kinetic resolution, asymmetric esterification, 2-aryl-2-fluoropropanoic acids, fluorinated drugs, 2-fluoroibuprofen, ibuprofen

## Abstract

We report a new method for the preparation of chiral 2-aryl-2-fluoropropanoic acids, including 2-fluoroibuprofen, a fluorinated analogue of non-steroidal anti-inflammatory drugs (NSAIDs), by the kinetic resolution of racemic 2-aryl-2-fluoropropanoic acids using enantioselective esterification. By applying pivalic anhydride (Piv_2_O) as a coupling agent, bis(*α*-naphthyl)methanol [(*α*-Np)_2_CHOH] as an achiral alcohol, and (+)-benzotetramisole (BTM) as a chiral acyl-transfer catalyst, a series of racemic 2-aryl-2-fluoropropanoic acids were kinetically separated to afford the optically active carboxylic acids and the corresponding esters with good to high enantiomeric excesses. This technology can provide a convenient approach to furnish the chiral *α*-fluorinated drugs containing quaternary carbons at the *α*-positions in the 2-aryl-2-fluoropropanoic acid structure.

## 1. Introduction

Recently, fluorinated compounds have grown in use in medicinal chemistry, because the insertion of a fluorine atom can dramatically change the metabolism, activity, and other properties of a compound [1]. With this trend, an increasing number of strategies for the syntheses of fluorinated drugs, which include the chiral quaternary carbons bonding with a fluorine atom, has been established [2]. Among these compounds, the 2-aryl-2-fluoropropanoic acids, such as 2-fluoroibuprofen, a fluorinated analogue of non-steroidal anti-inflammatory drugs (NSAIDs), have been frequently synthesized by several groups [3,4,5,6,7,8,9,10,11,12,13], because the substitution of a fluorine atom for a hydrogen atom at the *α*-positions in the 2-aryl-2-fluoropropanoic acids prohibits the unwanted epimerization of NSAIDs, which converts them from the biologically active chiral forms to less active epimerized forms *in vivo* [14,15,16].

**Scheme 1:**
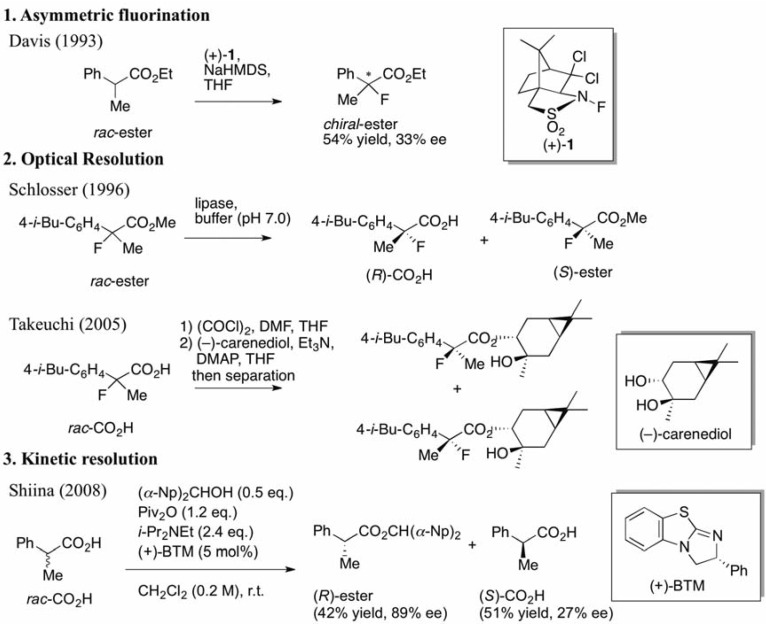
Previous studies for the preparation of chiral 2-aryl-2-fluoropropanoic acids (asymmetric fluorination, optical resolutions) and our kinetic resolution system.

In 1993, Davis *et al.* demonstrated the asymmetric fluorination of metal enolates, which were derived from carbonyl compounds, using *N*-fluoro dichlorocamphorsultam (+)-**1**, as illustrated in Scheme 1 [3]. Although the resulting enantioselectivities of the formed chiral 2-fluorinated compounds were unsatisfactory, their first trial was viewed as a pioneering study for the preparation of chiral 2-fluoro-2-arylpropanoic acids. 

Thereafter, two groups succeeded in developing practical methods for the synthesis of chiral 2-fluoroibuprofen using their original optical resolution systems (Scheme 1). Schlosser *et al.* kinetically separated a racemic mixture of carboxylic esters by enantioselective hydrolysis using lipase in 1996 [4]. On the other hand, Takeuchi *et al.* transformed a racemic mixture of carboxylic acids into a diastereomeric mixture of esters by esterification with a chiral alcohol, (–)-carenediol, and then they separated them by column chromatography in 2005 [5]. However, the former protocol required strict validations of the conditions and substrate tolerance of enzymes, while the latter presented difficulties in the quantitative use of the expensive chiral alcohol.

To address these issues, we introduced a non-enzymatic kinetic resolution system, that is, we established a new synthetic technology for the production of chiral 2-arylpropanoic acids including (*S*)-ibuprofen from a previous study (Scheme 1) [17,18]. The kinetic resolution efficiently functions using pivalic anhydride (Piv_2_O) as a coupling agent, bis(*α*-naphthyl)methanol [(*α*-Np)_2_CHOH] as an achiral nucleophile, and commercially available (+)-benzotetramisole (BTM) [19] as a chiral acyl-transfer catalyst. By only mixing racemic carboxylic acids with the coupling reagents and chiral BTM, the corresponding chiral carboxylic esters and the unreacted chiral carboxylic acids could be obtained in high enantiomeric excesses. Furthermore, in a mechanistic study, it was revealed that this enantioselective esterification proceeds through a mixed anhydride (MA), a reactive intermediate, formed from the substrate and the coupling reagent, followed by formation of a transition state (TS) with the achiral alcohol and chiral BTM [18]. To begin our study, we anticipated that the BTM-mediated kinetic resolution of the racemic 2-aryl-2-fluoropropanoic acids would successfully take place because of the similarity in size between the fluorine and hydrogen atoms. On the other hand, there was some concern that the electronegativity of the fluorine atom at the *α*-positions in 2-aryl-2-fluoropropanoic acid would negatively affect the selectivity in this system. In the present study, the kinetic resolution of the racemic 2-aryl-2-fluoropropanoic acids was examined and the detailed substrate scope was clarified.

## 2. Results and Discussion

In our previous study concerning the preparation of chiral 2-arylpropanoic acids [18], several successful optimizations of the reaction conditions identified the most suitable ratios of each reagent: 0.5 eq. of (*α*-Np)_2_CHOH, 1.2 eq. of Piv_2_O, 1.8 eq. of *i*-Pr_2_NEt, and 5 mol% of (+)-BTM. Therefore, we decided to introduce these optimized conditions to the current study, and began the investigation by screening solvents. The simple 2-fluoro-2-phenylpropanoic acid was selected as a model substrate, and four commonly-used solvents were employed in our initial screening (Table 1). 

**Table 1:** Screening of solvents. 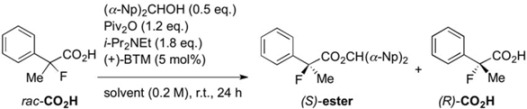

Entry	solvent	Yield ^a^ of ester/CO_2_H (%)	ee of ester/CO_2_H (%)	*s*
1	CH_2_Cl_2_	13/80	91/11	24
2	THF	31/52	92/43	36
3	DMF	18/46	90/17	23
4	Et_2_O	47/40	87/70	31

*^a^* isolated yield.

In order to evaluate the effectiveness of the kinetic resolution, we used a selectivity factor (*s*-value), which shows the ratio of reactivities of the (*R*)- and (*S*)-carboxylic acids [20]. In all entries, the enantiomeric excesses of the produced esters and *s*-values were dramatically high, while the yields of the esters depended significantly on the solvent used. THF gave the best *s*-value, but a deficient yield of the desired carboxylic ester was observed as shown by Entry 2 (31% yield, *s* = 36). Eventually, diethyl ether was determined as the most suitable solvent for the conversion of the starting carboxylic acid into the corresponding chiral ester (Entry 4, 47% yield, *s* = 31).

Next, with the conditions verified, a variety of 2-aryl-2-fluoropropanoic acids were examined in the investigation of the substrate scope (Table 2). In entries 2–9, carboxylic acids including a Me or Cl substituent at the *ortho*-, *meta*-, or *para*-position in the 2-phenyl group were employed. As a result, the substituent effect on the phenyl group clearly appeared. The *o*-tolyl group produced an excellent selectivity in this reaction system (Entry 2, *s* = 242) as well as the *o*-chlorophenyl group (Entry 6, *s* = 32). On the other hand, carboxylic acids having a *meta-*substituted phenyl group gave moderate selectivities compared to those having the *ortho*-substituted phenyl group (Entry 3, *s* = 21 and Entry 7, *s* = 13). Conversion yields of the carboxylic acids into the corresponding esters had been reduced in entries 4 and 8 (Entry 4, 29% yield and Entry 8, 20% yield) because precipitation of the salts, which were generated from the carboxylic acids with *i*-Pr_2_NEt, presumably prevented the progression of the asymmetric esterification. However, fine-tuning of the amounts of each reagent showed that reducing *i*-Pr_2_NEt to 0.5 eq. improved the conversion of the carboxylic acids into the desired esters (Entry 5, 45% yield and Entry 9, 48% yield).

**Table 2:** Kinetic resolution of 2-aryl-2-fluoropropanoic acids using (+)-BTM. 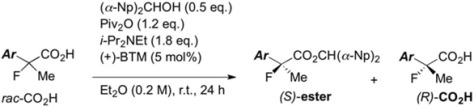

Entry	Ar	Yield ^b^ of ester/CO_2_H (%)	ee of ester/CO_2_H (%)	*s*
1	C_6_H_5_	47/40	87/70	31
2	*o*-Me-C_6_H_4_	40/47	98/68	242
3	*m*-Me-C_6_H_4_	38/47	84/61	21
4	*p*-Me-C_6_H_4_	29/36	90/42	29
5 ^a^	*p*-Me-C_6_H_4_	45/46	84/78	27
6	*o*-Cl-C_6_H_4_	36/56	91/43	32
7	*m*-Cl-C_6_H_4_	40/50	76/54	13
8	*p*-Cl-C_6_H_4_	20/62	81/21	12
9 ^a^	*p*-Cl-C_6_H_4_	48/44	75/74	16
10	*o*-F-C_6_H_4_	35/45	60/37	5.6
11	*o*-Br-C_6_H_4_	34/51	94/51	58
12	*o*-MeO-C_6_H_4_	18/29	89/20	20
13	*p*-*i*-Bu-C_6_H_4_	40/43	88/63	31

^a^ 0.5 eq. of *i*-Pr_2_NEt was used. *^b^* isolated yield.

Other *ortho*-substituted carboxylic acids were further investigated (Entries 10–12). The *o*-fluorophenyl group dramatically decreased the selectivity (Entry 10, *s* = 5.6), and it is speculated that the high electronegativity of the fluorine atom at the *ortho*-position in the phenyl group interrupted the enantioselective esterification. In contrast, the *o*-bromophenyl group did not inhibit selectivity, and a good selectivity was observed in Entry 11 (*s* = 58). When the reaction of racemic 2-aryl-2-fluoro-propanoic acids including the *o*-methoxyphenyl group was examined, rapid decomposition of the substrate proceeded that decreased the isolated yields of the desired ester and the recovered carboxylic acid (Entry 12). The kinetic resolution of the racemic 2-fluoroibuprofen also afforded the optically active ester of (*S*)-(+)-2-fluoroibuprofen in good yield with a high selectivity (Entry 13, 40% yield, 88% ee, *s* = 31).

**Scheme 2:**
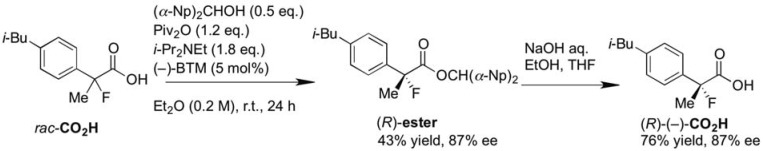
Production of (*R*)-(–)-2-fluoroibuprofen by the kinetic resolution using (–)-BTM.

Finally, we successfully prepared the chiral precursor of (*R*)-(–)-2-fluoroibuprofen by the present kinetic resolution using (–)-BTM as shown in Scheme 2, and hydrolysis of the formed ester produced the desired (*R*)-(–)-2-fluoroibuprofen (87% ee) ([α]_D_^27^ −26.9° (*c* 1.2, EtOH)), in which the absolute configuration was determined as *R* by comparison with the specific optical rotation of the identical compound ([α]_D_^24^ -22.6° (*c* 1.2, EtOH)) reported by Schlosser [4]. In a similar fashion, it is easy to consider that the other produced chiral esters of the 2-aryl-2-fluoropropanoic acids shown in Table 2 take *S* configuration.

The direction of the fluorine atom on the quaternary carbon in (*S*)-(+)-2-fluoroibuprofen, which was synthesized by the asymmetric esterification using (+)-BTM, identified with that of the hydrogen atom on the quaternary carbon in (*R*)-(–)-2-ibuprofen, which was similarly synthesized using the same (+)-catalyst [17]. Therefore, it is postulated that the high electronegativity of the fluorine atom at the *α*-position in 2-aryl-2-fluoropropanoic acid did not affect the stability of the transition structures in the chiral induction stage of the asymmetric esterification, and we anticipated that the fluorine atom works in the same manner as the hydrogen atom in the stereo-discriminating steps of this kinetic resolution system.

**Scheme 3:**
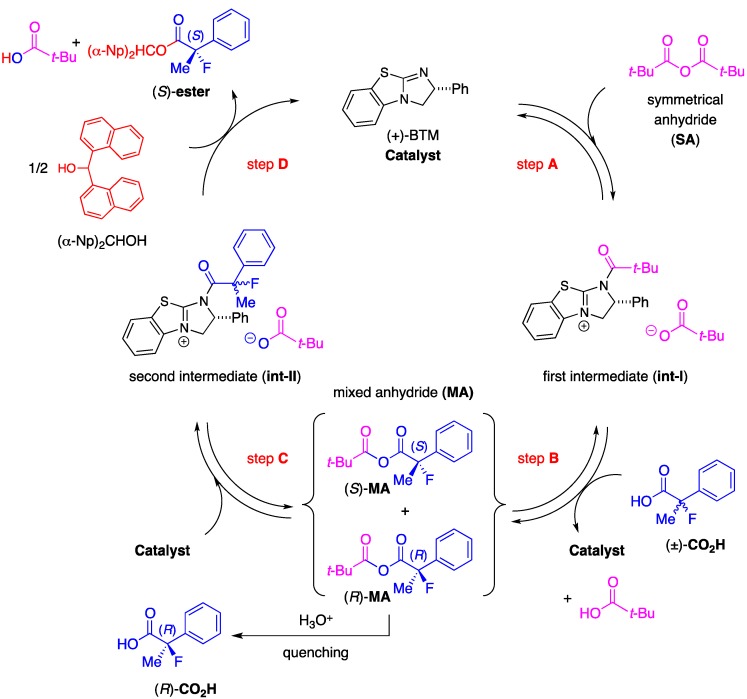
Plausible reaction pathway of the kinetic resolution of the racemic 2-fluoro-2-phenylpropanoic acid.

In order to enrich our understanding about the chiral recognition mechanism of the kinetic resolution of racemic 2-aryl-2-fluoropropanoic acids, the theoretical aspects of the asymmetric esterification were then considered. Combined with the perspectives detailed in our previous report [18], we estimated the catalytic cycle of this reaction as depicted in Scheme 3.

First, a mixed anhydride (**MA**) forms *in situ* as a key intermediate from the reaction of pivalic anhydride (Piv_2_O) with the racemic 2-fluoro-2-phenylpropanoic acid [(±)-**CO_2_H**] via generation of zwitterion (**int-I**) through steps **A** and **B** by promotion of acyl-transfer catalyst, (+)-BTM. In the next step **C**, (*S*)- and (*R*)-**MA** would be activated again by (+)-BTM to form the corresponding zwitterionic species (**int-II**), and half the amount of **int-II** generated from (*S*)-**MA** would selectively react with (*α*-Np)_2_CHOH to afford the desired (*S*)-carboxylic ester [(*S*)-**ester**] with high enantiomeric excess through step **D**. On the other hand, the remaining half of the mixed anhydride [(*R*)-**MA**] would be hydrolyzed to produce the unreacted (*R*)-2-fluoro-2-phenylpropanoic acid [(*R*)-**CO_2_H**] as a recovered optically active starting material with good enantiopurity. It is anticipated the step **D** is the key enantio-determining step during this multiple transacylation process. 

Based on these considerations described above, determination of the transition state forming the optically active (*S*)-**ester** from (*S*)-2-fluoro-2-phenylpropanoic acid with (*α*-Np)_2_CHOH, (+)-BTM, and Piv_2_O via **Int-II** was carried out using the density functional theory (DFT) calculations at the B3LYP/6-31G*//B3LYP/6-31G* level according to the method previously reported [18,21].

**Scheme 4:**
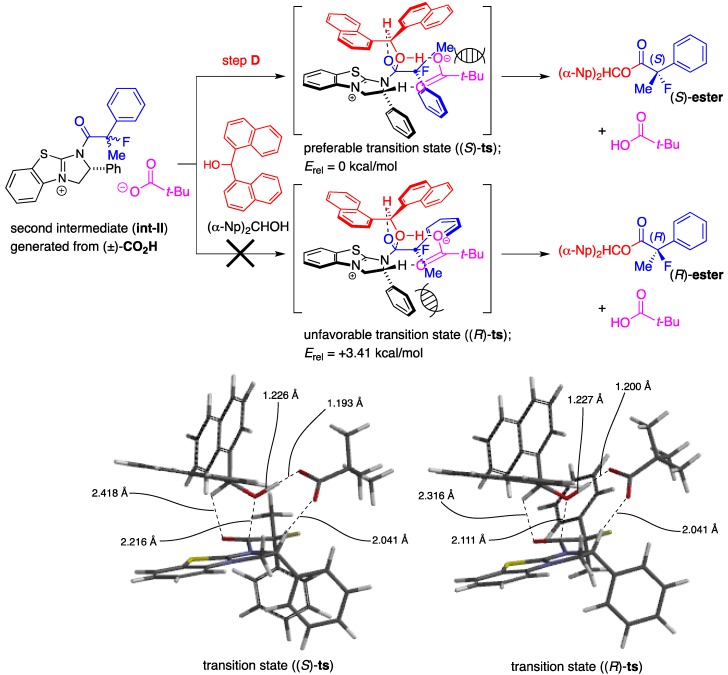
Calculated transition state of the kinetic resolution of racemic 2-fluoro-2-phenylpropanoic acid.

We successfully obtained the transition state ((*S*)-**ts**) to produce the desired ester (*S*)**-ester** as depicted in Scheme 4, and the high selectivity found to be attained in the present kinetic resolution could be explained by the rapid transformation of (*S*)-**CO_2_H** into (*S*)-**ester** via this stabilized transition structure consisting of the (*α*-Np)_2_CHOH and the dihydroimidazolium salt (**int-II**) derived from the mixed anhydride (*S*)-**MA** and (+)-BTM. The distance of the forming carbon-oxygen bond (between carbonyl carbon of the acid component and oxygen of hydroxyl) is 2.216 Å, accompanied with the coordination of oxygen in carbonyl moiety onto hydrogen at C-1 of alcohol at a distance of 2.418 Å. It is further observed that the distance of the cleaving oxygen-hydrogen bond (between oxygen and hydrogen in hydroxy) is 1.226 Å. 

A frequency analysis of (*S*)-**ts** revealed that the nucleophilic attack of the alcohol to carbonyl group and the deprotonation of the hydroxyl group with the pivalate anion proceeded under the concerted reaction mechanism because the carbon-oxygen bond-forming step and the oxygen-hydrogen bond-cleaving process occurred synchronously. The diarylcarbinol moiety of (*α*-Np)_2_CHOH in (*S*)-**ts** has a rigid structure in which the conformation is restricted by the attractive interaction between one of the naphthalene rings and the positive electronic charge on the face of the dihydroimidazolium salt as well as coordination of oxygens in the pivalate anion onto hydrogen in hydroxyl (1.193 Å) and hydrogen at C-2 of the dihydroimidazolium salt (2.041 Å). On the other hand, complexation of (*α*-Np)_2_CHOH with the dihydroimidazolium salt (**int-II**) including (*R*)-**MA** and (+)-BTM, an enantiomer of (*S*)-**MA**, produced an unstable structure (*R*)-**ts**, which has a much higher energy (*E*_rel_ = +3.41 kcal/mol) derived from steric repulsion between the methyl substituent at the *α*-position of (*R*)-**CO_2_H** and the phenyl group at C-2 of the dihydroimidazolium salt to afford the corresponding (*R*)-**ester**. Therefore, the desired chiral (*S*)-**ester** was selectively obtained by the rapid transformation of (*S*)-**MA** through the transition state (*S*)-**ts**. Each transition state (*S*)-**ts** or (*R*)-**ts** derived from 2-fluoro-2-phenylpropanoic acid has a very similar structure to that derived from 2-phenylpropanoic acid, which was disclosed in the former study [18]. Therefore, on the basis of the theoretical calculations, it is strongly supported that there is a close analogy between fluorine and hydrogen atoms at the *α*-positions in 2-fluoro-2-phenylpropanoic acid and 2-phenylpropanoic acid when the chiral recognition process takes place in the present asymmetric esterification.

## 3. Experimental

### 3.1. General

All melting points are uncorrected. ^1^H- and ^13^C-NMR spectra were recorded in chloroform-*d* with chloroform as internal standard. Column chromatography was performed on Silica gel 60 (Merck) or Wakogel B5F. Thin layer chromatography was performed on Wakogel B5F. All reactions were carried out under argon atmosphere in dried glassware, unless otherwise noted. Diethyl ether was distilled from sodium and benzophenone, dichloromethane was distilled from diphosphorus pentoxide, then calcium hydride, and dried over MS 4A, and THF and DMF were distilled from calcium hydride, and dried over MS 4A. All reagents were purchased from Tokyo Kasei Kogyo Co., Ltd (TCI), Kanto Chemical Co., Inc. or Aldrich Chemical Co., Inc., and used without further purification, unless otherwise noted. 

### 3.2. General Procedure for the Preparation of Racemic Ethyl 2-Fluoro-2-arylpropanoates ***2a–k***


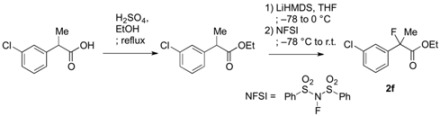


To a solution of 2-(3-chlorophenyl)propanoic acid {prepared from 2-(3-chlorophenyl)acetic acid according to [18] and [22]} (920 mg, 4.98 mmol) in ethanol (10 mL) at 0 °C was added sulfuric acid (3 mL). The reaction mixture was stirred at 0 °C for 5 min, and then refluxed for 7 h. After cooling to room temperature, the reaction mixture was diluted with water (30 mL) and extracted with ethyl acetate. The organic layer was washed with saturated aqueous NaHCO_3_ and brine, and then dried over sodium sulfate. After filtration of the mixture and evaporation of the solvent, ethyl 2-(3-chlorophenyl)propanoate (994 mg, 94% yield) was obtained as a pale yellow oil. This crude product was used in the next reaction without further purification. 

To a stirred solution of ethyl 2-(3-chlorophenyl)propanoate (987 mg, 4.74 mmol) in THF (10 mL) at −78 °C was added LHMDS in THF (1.0 M, 5.69 mL, 5.69 mmol). The mixture was stirred at −78 °C for 20 min, and then at 0 °C for 20 min. After cooling to −78 °C, *N*-fluorobenzenesulfonimide (NFSI) (1.87 g, 5.93 mmol) in THF (10 mL) was added to the reaction mixture. After gradually raised to room temperature for 8 h, the reaction mixture was diluted with 1 M hydrochloric acid (10 mL) and water (20 mL). The mixture was extracted with hexane, and the organic layer was washed with water, and then dried over sodium sulfate. After filtration of the mixture and evaporation of the solvent, the crude product was purified by silica gel column chromatography (ethyl acetate/hexane = 1/30) to afford **2f** (983 mg, 92% yield) as a colorless oil. If the fluorination was not sufficient and any precursor remained in the product, the fluorination process as described above was repeated.

#### *Ethyl 2-Fluoro-2-phenylpropanoate* (**2a**) [4,7]


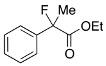


IR (neat): 1,754, 1,496, 1,123, 732, 696 cm^−1^; ^1^H-NMR (CDCl_3_): δ 7.53–7.48 (m, 2H, Ar), 7.42–7.29 (m, 3H, Ar), 4.24 (q, *J*_H-H_ = 7.2 Hz, 2H, OEt), 1.93 (d, *J*_H-F_ = 22.4 Hz, 3H, Me), 1.26 (t, *J*_H-H_ = 7.2 Hz, 3H, OEt); ^13^C-NMR (CDCl_3_): δ 170.9 (d, *J*_C-F_ = 26.4 Hz, 1), 139.3 (d, *J*_C-F_ = 21.9 Hz), 128.5 (d, *J*_C-F_ = 1.4Hz), 128.4, 124.6 (d, *J*_C-F_ = 8.1 Hz), 94.6 (d, *J*_C-F_ = 186.3 Hz, 2), 61.9 (Et), 24.8 (d, *J*_C-F_ = 23.5 Hz, 3), 14.0 (Et); HR MS: calcd for C11H13FO2Na (M+Na^+^) 219.0788, found 210.0792.

#### *Ethyl 2-Fluoro-2-(o-tolyl)propanoate* (**2b**)


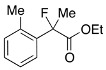


IR (neat): 1,751, 1,491, 1,117, 741 cm^−1^; ^1^H-NMR (CDCl_3_): δ 7.44–7.37 (m, 1H, Ar), 7.29–7.12 (m, 3H, Ar), 4.24 (q, *J*_H-H_ = 7.2 Hz, 2H, OEt), 2.37 (s, 3H, *o*-Me), 1.98 (d, *J*_H-F_ = 22.4 Hz, 3H, 2-Me), 1.25 (t, *J*_H-H_ = 7.2 Hz, 3H, OEt); ^>13^C-NMR (CDCl_3_): δ 171.5 (d, *J*_C-F_ = 26.5 Hz, 1), 136.7 (d, *J*_C-F_ = 20.5 Hz), 136.7, 131.9, 128.9 (d, *J*_C-F_ = 1.4 Hz), 125.9 (d, *J*_C-F_ = 7.3 Hz), 125.8, 95.0 (d, *J*_C-F_ = 183.3 Hz, 2), 61.9 (Et), 24.1 (d, *J*_C-F_ = 25.0 Hz, 3), 20.2 (d, *J*_C-F_ = 5.1 Hz, *o*-Me), 14.0 (Et); HR MS: calcd for C12H15FO2Na (M+Na^+^) 233.0948, found 233.0944.

#### *Ethyl 2-Fluoro-2-(m-tolyl)propanoate* (**2c**)


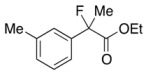


IR (neat): 1,755, 1,608, 1,489, 1,122, 852, 731, 697 cm^−1^; ^1^H-NMR (CDCl_3_): δ 7.34–7.22 (m, 3H, Ar), 7.20–7.12 (m, 1H, Ar), 4.22 (q, *J*_H-H_ = 7.2 Hz, 2H, OEt), 2.37 (s, 3H, *m*-Me), 1.91 (d, *J*_H-F_ = 22.0 Hz, 3H, 2-Me), 1.26 (t, *J*_H-H_ = 7.2 Hz, 3H, OEt); ^13^C-NMR (CDCl_3_): δ 71.0 (d, *J*_C-F_ = 27.2 Hz, 1), 139.3 (d, *J*_C-F_ = 22.7 Hz), 138.2, 129.3 (d, *J*_C-F_ = 1.4 Hz), 128.3, 125.2 (d, *J*_C-F_ = 8.9 Hz), 121.6 (d, *J*_C-F_ = 8.2 Hz), 94.7 (d, *J*_C-F_ = 186.4 Hz, 2), 61.9 (Et), 24.8 (d, *J*_C-F_ = 24.8 Hz, 3), 21.5 (*m*-Me), 14.0 (Et); HR MS: calcd for C12H15FO2Na (M+Na^+^) 233.0948, found 233.0941.

#### *Ethyl 2-Fluoro-2-(p-tolyl)propanoate* (**2d**) [7]


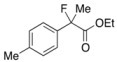


IR (neat): 1,754, 1,616, 1,514, 1,114, 818, 739 cm^−1^; ^1^H-NMR (CDCl_3_): δ 7.41–7.33 (m, 2H, Ar), 7.21–7.09 (m, 2H, Ar), 4.21 (q, *J*_H-H_ = 7.2 Hz, 2H, OEt), 2.35 (s, 3H, *p*-Me), 1.91 (d, *J*_H-F_ = 22.4 Hz, 3H, 2-Me), 1.25 (t, *J*_H-H_ = 7.2 Hz, 3H, OEt); ^13^C-NMR (CDCl_3_): δ 171.0 (d, *J*_C-F_ = 27.2 Hz, 1), 138.4 (*J*_C-F_ = 1.4 Hz), 136.4 (*J*_C-F_ = 22.7 Hz), 129.1, 124.6 (*J*_C-F_ = 8.2 Hz), 94.6 (*J*_C-F_ = 185.7 Hz, 2), 61.9 (Et), 24.7 (*J*_C-F_ = 24.2 Hz, 3), 21.1 (*p*-Me), 14.0 (Et); HR MS: calcd for C12H15FO2Na (M+Na^+^) 233.0948, found 233.0937.

#### *Ethyl 2-(2-Chlorophenyl)-2-fluoropropanoate* (**2e**)


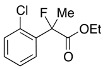


IR (neat): 1,747, 1,596, 1,474, 1,125, 750 cm^−1^; ^1^H-NMR (CDCl_3_): δ 7.63–7.58 (m, 1H, Ar), 7.41–7.27 (m, 3H, Ar), 4.33–4.20 (m, 2H, OEt), 1.96 (d, *J*_H-F_ = 23.6 Hz, 3H, 2-Me), 1.26 (t, *J*_H-H_ = 7.2 Hz, 3H, OEt); ^13^C-NMR (CDCl_3_): δ 69.5 (d, *J*_C-F_ = 2 5.0 Hz, 1), 137.4 (d, *J*_C-F_ = 22.0 Hz), 130.5, 131.5 (d, *J*_C-F_ = 3.7 Hz), 130.5, 129.9 (d, *J*_C-F_ = 1.5 Hz), 126.7 (d, *J*_C-F_ = 12.5 Hz), 94.4 (d, *J*_C-F_ = 180.4 Hz, 2), 62.2 (Et), 23.1 (d, *J*_C-F_ = 25.0 Hz, 3), 13.9 (Et); HR MS: calcd for C_11_H_12_ClFO_2_Na (M+Na^+^) 253.0402, found 253.0405.

#### *Ethyl 2-(3-Chlorophenyl)-2-fluoropropanoate* (**2f**)


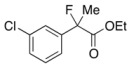


IR (neat): 1,756, 1,598, 1,477, 1,125, 792, 712 cm^−1^; ^1^H-NMR (CDCl_3_): δ 7.52–7.49 (m, 1H, Ar), 7.41–7.28 (m, 3H, Ar), 4.23 (q, *J*_H-H_ = 7.2 Hz, 2H, OEt), 1.91 (d, *J*_H-F_ = 22.4 Hz, 3H, 2-Me), 1.27 (t, *J*_H-H_ = 7.2 Hz, 3H, OEt); ^13^C-NMR (CDCl_3_): δ 70.3 (d, *J*_C-F_ = 26.4 Hz, 1), 141.3 (d, *J*_C-F_ = 22.7 Hz), 134.5 (d, *J*_C-F_ = 1.4 Hz), 129.8, 128.8, 125.0 (d, *J*_C-F_ = 9.5 Hz), 122.8 (d, *J*_C-F_ = 8.2 Hz), 94.1 (d, *J*_C-F_ = 188.5 Hz, 2), 62.2 (Et), 24.9 (d, *J*_C-F_ = 24.3 Hz, 3), 13.7 (Et); HR MS: calcd for C_11_H_12_ClFO_2_Na (M+Na^+^) 253.0402, found 253.0404.

#### *Ethyl 2-(4-Chlorophenyl)-2-fluoropropanoate* (**2g**) [4,10]


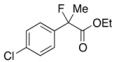


IR (neat): 1,755, 1,600, 1,492, 1,125, 1,092, 833, 761 cm^−1^; ^1^H-NMR (CDCl_3_): δ 7.47–7.41 (m, 2H, Ar), 7.39–7.32 (m, 2H, Ar), 4.22 (q, *J*_H-H_ = 7.2 Hz, 2H, OEt), 1.91 (d, *J*_H-F_ = 22.0 Hz, 3H, 2-Me), 1.26 (t, *J*_H-H_ = 7.2 Hz, 3H, OEt); ^13^C-NMR (CDCl_3_): δ 70.5 (d, *J*_C-F_ = 26.5 Hz, 1), 137.9 (d, *J*_C-F_ = 23.4 Hz), 134.7 (d, *J*_C-F_ = 2.2Hz), 128.6, 126.1 (d, *J*_C-F_ = 8.8 Hz), 94.2 (d, *J*_C-F_ = 187.1 Hz, 2), 62.1 (Et), 24.8 (d, *J*_C-F_ = 24.2 Hz, 3), 14.0 (Et); HR MS: calcd for C_11_H_12_ClFO_2_Na (M+Na^+^) 253.0402, found 253.0410.

#### *Ethyl 2-Fluoro-2-(2-fluorophenyl)propanoate* (**2h**)


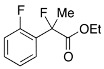


IR (neat): 1,747, 1,617, 1,586, 1,492, 1,121, 1,100, 754 cm^−1^; ^1^H-NMR (CDCl_3_): δ 7.56–7.49 (m, 1H, Ar), 7.39–7.32 (m, 1H, Ar), 7.22–7.15 (m, 1H, Ar), 7.11–7.01 (m, 1H, Ar), 4.19–4.32 (m, 2H, OEt), 1.96 (d, *J*_H-F_ = 22.8 Hz, 3H, 2-Me), 1.26 (t, *J*_H-H_ = 7.2 Hz, 3H, OEt); ^13^C-NMR (CDCl3): δ 169.9 (d, *J*_C-F_ = 26.4 Hz, 3), 159.6 (dd, *J*_C-F_ = 249.5, 4.4 Hz), 130.7 (dd, *J*_C-F_ = 8.1, 1.5 Hz), 126.9 (d, *J*_C-F_ = 12.5 Hz), 126.7 (dd, *J*_C-F_ = 9.9, 3.3 Hz), 124.1 (d, *J*_C-F_ = 3.0 Hz), 116.1 (d, *J*_C-F_ = 22.0 Hz), 92.5 (d, *J*_C-F_ = 182.6 Hz, 2), 62.1 (Et), 23.4 (dd, *J*_C-F_ = 24.6, 2.6 Hz, 1), 13.9 (Et); HR MS: calcd for C_11_H_12_F_2_O_2_Na (M+Na^+^) 237.0698, found 237.0706.

#### *Ethyl 2-(2-Bromophenyl)-2-fluoropropanoate* (**2i**)


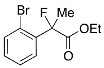


IR (neat): 1,746, 1,592, 1,471, 1,124, 748, 638 cm^−1^; ^1^H-NMR (CDCl_3_): δ 7.64–7.55 (m, 2H, Ar), 7.42–7.34 (m, 1H, Ar), 7.25–7.18 (m, 1H, Ar), 4.27 (q, *J*_H-H_ = 7.2 Hz, 2H, OEt), 1.98 (d, *J*_H-F_ = 23.6 Hz, 3H, 2-Me), 1.27 (t, *J*_H-H_ = 7.2 Hz, 3H, OEt); ^13^C-NMR (CDCl3): δ 169.3 (d, *J*_C-F_ = 24.9 Hz, 1), 139.0 (d, *J*_C-F_ = 21.2 Hz), 134.0, 130.0 (d, *J*_C-F_ = 1.5 Hz), 127.4 (d, *J*_C-F_ = 1.5 Hz), 127.0 (d, *J*_C-F_ = 13.2 Hz), 120.6 (d, *J*_C-F_ = 3.6 Hz), 95.2 (d, *J*_C-F_ = 180.5 Hz, 2), 62.2 (Et), 23.3 (d, *J*_C-F_ = 25.0 Hz, 3), 13.9 (Et); HR MS: calcd for C_11_H_12_BrFO_2_Na (M+Na^+^) 296.9897, found 296.9893.

#### *Ethyl 2-Fluoro-2-(2-methoxyphenyl)propanoate* (**2j**)


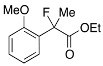


IR (neat): 1,746, 1,604, 1,589, 1,492, 1,116, 752 cm^−1^; ^1^H-NMR (CDCl_3_): δ7.52–7.46 (m, 1H, Ar), 7.38–7.30 (m, 1H, Ar), 7.05–6.96 (m, 1H, Ar), 6.92–6.86 (m, 1H, Ar), 4.28–4.18 (m, 2H, OEt), 3.79 (s, 3H, OMe), 1.89 (d, *J*_H-F_ = 22.8 Hz, 3H, 2-Me), 1.24 (t, *J*_H-H_ = 7.2 Hz, 3H, OEt); ^13^C-NMR (CDCl3): δ 170.8 (d, *J*_C-F_ = 25.0 Hz, 1), 156.1 (d, *J*_C-F_ = 4.4 Hz), 130.1 (d, *J*_C-F_ = 2.2 Hz), 128.1 (d, *J*_C-F_ = 21.2 Hz), 125.9 (d, *J*_C-F_ = 9.6 Hz), 120.6, 111.1, 93.1 (d, *J*_C-F_ = 179.7 Hz, 2), 61.4 (Et), 55.4 (OMe), 23.0 (d, *J*_C-F_ = 25.0 Hz, 3), 14.0 (Et); HR MS: calcd for C_12_H_15_FO_3_Na (M+Na^+^) 249.0897, found 249.0907.

#### *Ethyl 2-Fluoro-2-(4-isobutylphenyl)propanoate* (**2k**) [4,6,7,10]


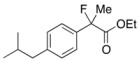


IR (neat): 1,756, 1,614, 1,512, 1,116, 849 cm^−1^; ^1^H-NMR (CDCl_3_): δ 7.45–7.35 (m, 2H, Ar), 7.17–7.10 (m, 2H, Ar), 4.22 (q, *J*_H-H_ = 7.2 Hz, 2H, OEt), 2.47 (d, *J*_H-H_ = 7.2 Hz, 2H, *i*-Bu), 2.00 (d, *J*_H-F_ = 22.4 Hz, 3H, 2-Me), 1.92-1.78 (m, 1H, *i*-Bu), 1.26 (t, *J*_H-H_ = 7.2 Hz, 3H, OEt), 0.90 (d, *J*_H-H_ = 6.8 Hz, 6H, *i*-Bu); ^13^C-NMR (CDCl3): δ 171.1 (d, *J*_C-F_ = 27.2 Hz, 1), 142.2 (d, *J*_C-F_ = 1.4 Hz), 136.6 (d, *J*_C-F_ = 22.7 Hz), 129.1, 124.4 (d, *J*_C-F_ = 8.1 Hz), 94.6 (d, *J*_C-F_ = 185.6 Hz, 2), 61.9 (Et), 45.0 (*i*-Bu), 30.1 (*i*-Bu), 24.7 (d, *J*_C-F_ = 24.3 Hz, 3), 22.3 (*i*-Bu), 14.0 (Et); HR MS: calcd for C_15_H_21_FO_2_Na (M+Na^+^) 275.1418, found 275.1410.

### 3.3. General Procedure for the Preparation of Racemic 2-Fluoro-2-arylpropanoic Acids ***3a–k***


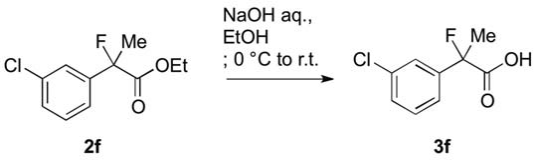


To a solution of ethyl 2-(3-chlorophenyl)-2-fluoropropanoate (**2f**, 868 mg, 3.76 mmol) in ethanol (10 mL) at 0 °C was added aqueous sodium hydroxide (4.2 M, 4 mL, 16.8 mmol). The reaction mixture was stirred at room temperature for 3 h, and then it was acidified with 1 M hydrochloric acid (30 mL). The mixture was extracted with ethyl acetate, and the organic layer was dried over sodium sulfate. After filtration of the mixture and evaporation of the solvent, the crude product was recrystallized from dichloromethane and hexane to afford **3f** (420 mg, 55% yield) as a white solid.

### 3.4. Procedure for Kinetic Resolution of Racemic 2-Fluoro-2-arylpropanoic Acid


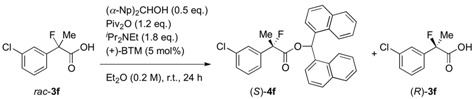


To a mixture of 2-(3-chlorophenyl)-2-fluoropropanoic acid (**3f**) (40.5 mg, 0.200 mmol), pivalic anhydride (48.7 μL, 0.240 mmol), and bis(*α*-naphthyl)methanol (28.4 mg, 0.100 mmol) in diethyl ether (1.0 mL) at room temperature were successively added diisopropylethylamine (62.7 μL, 0.360 μmol) and (+)-BTM (2.5 mg, 10 μmol). The reaction mixture was stirred for 24 h at room temperature, and then quenched with 1 M hydrochloric acid. The mixture was extracted with ethyl acetate, and the organic layer was dried over sodium sulfate. After filtration of the mixture and evaporation of the solvent, the crude product was purified by preparative thin layer chromatography on silica (toluene/hexane = 90/10) to afford the corresponding ester (*S*)-**4f** (38.6 mg, 40% yield, 76% ee) as a colorless oil. The polar fraction including **3f** was further purified by preparative thin layer chromatography on silica (ethyl acetate/hexane/formic acid = 10/40/1) to afford the recovered optically active (*R*)-**3f** (20.4 mg, 50% yield, 54% ee) as a white solid.

#### *2-Fluoro-2-phenylpropanoic Acid* ((*R*)-**3a**) [4,8,13] [Table 1, Entry 4, and Table 2, Entry 1, 70% ee]


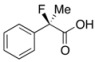


HPLC (CHIRALPAK OJ-H, *i*-PrOH/hexane/TFA = 1/10/0.01, flow rate = 1.0 mL/min): *t*_R_ = 11.7 min (15.0%), *t*_R_ = 13.9 min (85.0%); ^1^H-NMR (CDCl_3_): δ 10.47 (br s, 1H, CO_2_H), 7.62–7.28 (m, 5H, Ar), 1.96 (d, *J*_H-F_ = 22.4 Hz, 3H, Me); ^13^C-NMR (CDCl_3_): δ 176.6 (d, *J*_C-F_ = 28.7 Hz, 1), 138.3 (d, *J*_C-F_ = 21.9 Hz), 128.9 (d, *J*_C-F_ = 1.4 Hz), 128.6, 124.7 (d, *J*_C-F_ = 8.8 Hz), 94.1 (d, *J*_C-F_ = 186.4 Hz, 2), 24.4 (d, *J*_C-F_* = *23.4 Hz, 3); HR MS: calcd for C_9_H_9_FO_2_Na (M+Na^+^) 191.0479, found 191.0479. Analytical data on racemic compound: Mp: 54-56 °C (hexane); IR (KBr): 2,939, 1,716, 1,601, 1,496, 1,146, 727, 697 cm^−1^.

#### *2-Fluoro-2-(o-tolyl)propanoic Acid* ((*R*)-**3b/**) [Table 2, Entry 2, 68% ee]


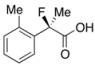


HPLC (CHIRALPAK OJ-H, *i*-PrOH/hexane/TFA = 1/10/0.01, flow rate = 0.75 mL/min): *t*_R_ = 14.4 min (83.9%), *t*_R_ = 18.2 min (16.1%); ^1^H-NMR (CDCl_3_): δ 9.56 (br s, 1H, CO_2_H), 7.46–7.16 (m, 4H, Ar), 2.42 (d, *J*_H-F_ = 3.2 Hz, 3H, *o*-Me), 2.04 (d, *J*_H-F_ = 22.4 Hz, 3H, 2-Me); ^13^C-NMR (CDCl_3_): δ 176.6 (d, *J*_C-F_ = 27.7 Hz, 1), 137.0, 135.6 (d, *J*_C-F_ = 20.6 Hz), 132.1 (d, *J*_C-F_ = 1.4 Hz), 129.4 (d, *J*_C-F_ = 1.4 Hz), 126.2 (d, *J*_C-F_ = 6.5 Hz), 125.9, 94.6 (d, *J*_C-F_ = 184.2 Hz, 2), 24.0 (d, *J*_C-F_ = 25.0 Hz, 3), 20.4 (d, *J*_C-F_ = 5.8 Hz, *o*-Me); HR MS: calcd for C_10_H_10_FO_2_ (M−H^+^) 181.0665, found 181.0663. Analytical data on racemic compound: Mp: 105–106 °C (CH_2_Cl_2_/hexane); IR (KBr): 2,926, 1,733, 1,605, 1,492, 1,126, 729 cm^−1^.

#### *2-Fluoro-2-(m-tolyl)propanoic Acid* ((*R*)-**3c**) [Table 2, Entry 3, 61% ee]


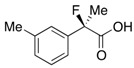


HPLC (CHIRALPAK AS-H, *i*-PrOH/hexane/TFA = 1/50/0.05, flow rate = 0.75 mL/min): *t*_R_ = 19.3 min (80.5%), *t*_R_ = 23.2 min (19.5%); ^1^H-NMR (CDCl_3_): δ 9.46 (br s, 1H, CO_2_H), 7.38–7.14 (m, 4H, Ar), 2.37 (s, 3H, *m*-Me) 1.95 (d, *J*_H-F_ = 22.4 Hz, 3H, 2-Me); ^13^C-NMR (CDCl_3_): δ 76.3 (d, *J*_C-F_ = 27.9 Hz, 1), 138.4, 138.2 (d, *J*_C-F_ = 22.7 Hz), 129.7 (d, *J*_C-F_ = 1.5 Hz), 128.5, 125.3 (d, *J*_C-F_ = 8.1 Hz), 121.8 (d, *J*_C-F_ = 8.1 Hz), 94.2 (d, *J*_C-F_ = 186.3 Hz, 2), 24.4 (d, *J*_C-F_ = 24.3 Hz, 3), 21.5 (*m*-Me); HR MS: calcd for C_10_H_10_FO_2_Na (M+Na^+^) 205.0635, found 205.0629. Analytical data on racemic compound: Mp: 36–39 °C (hexane); IR (KBr): 2,919, 1,713, 1,609, 1,488, 1,129, 827, 721, 697 cm^−1^.

#### 2*-Fluoro-2-(p-tolyl)propanoic Acid* ((*R*)-**3d**) [Table 2, Entry 5, 78% ee]


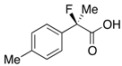


HPLC (CHIRALPAK OJ-H, *i*-PrOH/hexane/TFA = 1/10/0.01, flow rate = 0.75 mL/min): *t*_R_ = 16.3 min (10.9%), *t*_R_ = 21.2 min (89.1%); ^1^H-NMR (CDCl_3_): δ 8.42 (br s, 1H, CO_2_H), 7.47–7.32 (m, 2H, Ar), 7.23–7.13 (m, 2H, Ar), 2.35 (s, 3H, *p*-Me), 1.90 (d, *J*_H-F_ = 22.0 Hz, 3H, 2-Me); ^13^C-NMR (CDCl_3_): δ 176.2 (d, *J*_C-F_ = 28.0 Hz, 1), 138.9 (*J*_C-F_ = 1.4 Hz), 135.4 (*J*_C-F_ = 22.7 Hz), 129.3, 124.7 (*J*_C-F_ = 8.1 Hz), 94.2 (*J*_C-F_ = 186.4 Hz, 2), 24.3 (*J*_C-F_ = 23.4 Hz, 3), 21.1 (*p*-Me); HR MS: calcd for C_10_H_10_FO_2_Na (M+Na^+^) 205.0635, found 205.0628. Analytical data on racemic compound: Mp: 68–71 °C (hexane); IR (KBr): 2,997, 1,711, 1,613, 1,513, 1,114, 818, 731 cm^−1^.

#### *2-(2-Chlorophenyl)-2-fluoropropanoic Acid* ((*R*)-**3e**) [Table 2, Entry 6, 43% ee]


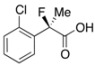


HPLC (CHIRALPAK OJ-H, *i*-PrOH/hexane/TFA = 1/10/0.01, flow rate = 0.75 mL/min): *t*_R_ = 15.8 min (71.4%), *t*_R_ = 17.5 min (28.6%); ^1^H-NMR (CDCl_3_): δ 9.65 (br s, 1H, CO_2_H), 7.64–7.56 (m, 1H, Ar), 7.45–7.29 (m, 3H, Ar), 2.02 (d, *J*_H-F_ = 23.2 Hz, 3H, 2-Me); ^13^C-NMR (CDCl_3_): δ 75.2 (d, *J*_C-F_ = 23.2 Hz, 1), 136.2 (d, *J*_C-F_ = 21.2 Hz), 132.1 (d, *J*_C-F_ = 3.7 Hz), 130.7, 130.4 (d, *J*_C-F_ = 1.4 Hz), 127.0 (d, *J*_C-F_ = 10.3 Hz), 127.0, 93.9 (d, *J*_C-F_ = 181.9 Hz, 2), 23.0 (d, *J*_C-F_ = 23.0 Hz, 3); HR MS: calcd for C_9_H_7_ClFO_2_ (M–H^+^) 201.0119, found 201.0114. Analytical data on racemic compound: Mp: 114–116 °C (CH_2_Cl_2_/hexane); IR (KBr): 2,998, 1,712, 1,594, 1,475, 1,137, 748 cm^−1^.

#### *2-(3-Chlorophenyl)-2-fluoropropanoic Acid* ((*R*)-**3f**) [Table 2, Entry 8, 54% ee]


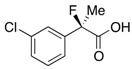


HPLC (CHIRALPAK OJ-H, *i*-PrOH/hexane/TFA = 1/10/0.01, flow rate = 0.75 mL/min): *t*_R_ = 8.7 min (22.9%), *t*_R_ = 10.1 min (77.1%); ^1^H-NMR (CDCl_3_): δ 8.84 (br s, 1H, CO_2_H), 7.58–7.49 (m, 1H, Ar), 7.48–7.30 (m, 3H, Ar), 1.95 (d, *J*_H-F_ = 22.0 Hz, 3H, 2-Me); ^13^C-NMR (CDCl_3_): δ 75.2 (d, *J*_C-F_ = 27.9 Hz, 1), 140.2 (d, *J*_C-F_ = 22.7 Hz), 134.7 (d, *J*_C-F_ = 1.4 Hz), 129.9, 129.2, 125.1 (d, *J*_C-F_ = 9.6 Hz), 122.9 (d, *J*_C-F_ = 8.8 Hz), 93.7 (d, *J*_C-F_ = 187.8 Hz, 2), 24.6 (d, *J*_C-F_ = 23.4 Hz, 3); HR MS: calcd for C_9_H_7_ClFO_2_ (M–H^+^) 201.0119, found 201.0116. Analytical data on racemic compound: Mp: 64–66 °C (CH_2_Cl_2_/hexane); IR (KBr): 2,986, 1,714, 1,597, 1,479, 1,146, 797, 702 cm^−1^.

#### *2-(4-Chlorophenyl)-2-fluoropropanoic Acid* ((*R*)-**3g**) [4] [Table 2, Entry 9, 74% ee]


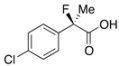


HPLC (CHIRALPAK OJ-H, *i*-PrOH/hexane/TFA = 1/50/0.05, flow rate = 0.75 mL/min): *t*_R_ = 33.6 min (13.0%), *t*_R_ = 37.1 min (87.0%); ^1^H-NMR (CDCl_3_): δ 9.16 (br s, 1H, CO_2_H), 7.50–7.43 (m, 2H, Ar), 7.42–7.34 (m, 2H, Ar), 1.95 (d, *J*_H-F_ = 22.4 Hz, 3H, 2-Me); ^13^C-NMR (CDCl_3_): δ 75.4 (d, *J*_C-F_ = 27.9 Hz, 1), 136.8 (d, *J*_C-F_ = 22.7 Hz), 135.2 (d, *J*_C-F_ = 1.5 Hz), 128.8, 126.2 (d, *J*_C-F_ = 8.9 Hz), 93.9 (d, *J*_C-F_ = 187.1 Hz, 2), 24.5 (d, *J*_C-F_ = 23.4 Hz, 3); HR MS: calcd for C_9_H_7_ClFO_2_ (M–H^+^) 201.0119, found 201.0117. Analytical data on racemic compound: Mp: 90–92 °C (CH_2_Cl_2_/hexane); IR (KBr): 2,995, 1,722, 1,599, 1,493, 1,127, 1,098, 830, 761 cm^−1^.

#### *2-Fluoro-2-(2-fluorophenyl)propanoic Acid* ((*R*)-**3h**) [Table 2, Entry 10, 37% ee]


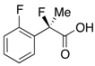


HPLC (CHIRALPAK OJ-H, *i*-PrOH/hexane/TFA = 1/50/0.05, flow rate = 0.75 mL/min): *t*_R_ = 64.9 min (68.5%), *t*_R_ = 81.5 min (31.5%); ^1^H-NMR (CDCl_3_): δ 9.48 (br s, 1H, CO_2_H), 7.59–7.49 (m, 1H, Ar), 7.43–7.34 (m, 1H, Ar), 7.23–7.16 (m, 1H, Ar), 7.15–7.05 (m, 1H, Ar), 2.02 (d, *J*_H-F_ = 22.8 Hz, 3H, 2-Me); ^13^C-NMR (CDCl3): δ 175.3 (d, *J*_C-F_ = 27.2 Hz, 1), 159.8 (dd, *J*_C-F_ = 249.5, 4.4 Hz), 131.2 (dd, *J*_C-F_ = 8.8, 1.5 Hz), 126.9 (dd, *J*_C-F_ = 9.2, 3.3 Hz), 125.6 (dd, *J*_C-F_ = 22.7, 12.5 Hz), 124.2 (d, *J*_C-F_ = 3.6 Hz), 116.3 (d, *J*_C-F_ = 21.9 Hz), 92.1 (d, *J*_C-F_ = 184.2 Hz, 2), 23.0 (dd, *J*_C-F_ = 24.2, 2.9 Hz, 3); HR MS: calcd for C_9_H_7_F_2_O_2_ (M−H+) 185.0414, found 185.0412. Analytical data on racemic compound: Mp: 116–117 °C (CH_2_Cl_2_/hexane); IR (KBr): 2,984, 1,734, 1,618, 1,587, 1,510, 1,493, 1,124, 1,101, 815, 762, 749 cm^−1^.

#### *2-(2-Bromophenyl)-2-fluoropropanoic Acid* ((*R*)-**3i**) [Table 2, Entry 11, 51% ee]


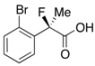


HPLC (CHIRALPAK OJ-H, *i*-PrOH/hexane/TFA = 1/10/0.01, flow rate = 0.75 mL/min): *t*_R_ = 18.6 min (75.6%), *t*_R_ = 22.3 min (24.4%); ^1^H-NMR (CDCl_3_): δ 9.73 (br s, 1H, CO_2_H), 7.67–7.55 (m, 2H, Ar), 7.43–7.34 (m, 1H, Ar), 7.30–7.19 (m, 1H, Ar), 2.04 (d, *J*_H-F_ = 23.6 Hz, 3H, 2-Me); ^13^C-NMR (CDCl3): δ 174.7 (d, *J*_C-F_ = 25.0 Hz, 1), 137.7 (d, *J*_C-F_ = 20.5 Hz), 134.3, 130.5 (d, *J*_C-F_ = 1.4 Hz), 127.5, 127.4 (d, *J*_C-F_ = 11.0 Hz), 121.2 (d, *J*_C-F_ = 2.9 Hz), 94.8 (d, *J*_C-F_ = 181.9 Hz, 2), 23.2 (d, *J*_C-F_ = 25.0 Hz, 3); HR MS: calcd for C_9_H_7_BrFO_2_ (M−H+) 244.9613, found 244.9612. Analytical data on racemic compound: Mp: 95–96 °C (CH_2_Cl_2_/hexane); IR (KBr): 3,000, 1,723, 1,591, 1,572, 1,474, 1,132, 760, 745, 641 cm^−1^.

#### *2-Fluoro-2-(2-methoxyphenyl)propanoic Acid* ((*R*)-**3j**) [Table 2, Entry 12, 20% ee]


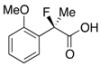


HPLC (CHIRALPAK OJ-H, *i*-PrOH/hexane/TFA = 1/50/0.05, flow rate = 0.75 mL/min): *t*_R_ = 48.9 min (59.8%), *t*_R_ = 54.5 min (40.2%); ^1^H-NMR (CDCl_3_): δ 9.23 (br s, 1H, CO_2_H), 7.53–7.47 (m, 1H, Ar), 7.45–7.31 (m, 1H, Ar), 7.11–6.98 (m, 1H, Ar), 7.98–6.84 (m, 1H, Ar), 3.82 (s, 3H, OMe), 1.96 (d, *J*_H-F_ = 22.4 Hz, 3H, 2-Me); ^13^C-NMR (CDCl3): δ 175.8 (d, *J*_C-F_ = 26.4 Hz, 1), 156.6 (d, *J*_C-F_ = 3.0 Hz), 130.8 (d, *J*_C-F_ = 2.2 Hz), 126.9 (d, *J*_C-F_ = 20.5 Hz), 120.8, 111.7, 92.8 (d, *J*_C-F_ = 181.2 Hz, 2), 55.6 (OMe), 22.7 (d, *J*_C-F_ = 25.0 Hz, 3); HR MS: calcd for C_10_H_10_FO_3_ (M−H^+^) 197.0614, found 197.0605. Analytical data on racemic compound: Mp: 83–84 °C (CH_2_Cl_2_/hexane); IR (KBr): 2,986, 1,721, 1,604, 1,587, 1,495, 1,125, 754 cm^−1^.

#### *2-Fluoro-2-(4-isobutylphenyl)propanoic Acid* ((*R*)-**3k**) [4,5,8,9,11,12,13] [Table 2, Entry 13, 63% ee]


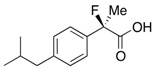


HPLC (CHIRALPAK AS-H, *i*-PrOH/hexane/TFA = 1/50/0.05, flow rate = 0.75 mL/min): *t*_R_ = 15.1 min (81.5%), *t*_R_ = 17.9 min (18.5%); ^1^H-NMR (CDCl_3_): δ 9.83 (br s, 1H, CO_2_H), 9.44–9.39 (m, 2H, Ar), 9.19–9.13 (m, 2H, Ar), 2.47 (d, *J*_H-H_ = 7.2 Hz, 2H, *i*-Bu), 1.95 (d, *J*_H-F_ = 22.0 Hz, 3H, 2-Me), 1.91–1.80 (m, 1H, *i*-Bu), 0.90 (d, *J*_H-H_ = 6.8 Hz, 6H, *i*-Bu); ^13^C-NMR (CDCl3): δ 176.0 (d, *J*_C-F_ = 27.9 Hz, 1), 142.7, 135.6 (d, *J*_C-F_ = 22.7 Hz), 129.3, 124.5 (d, *J*_C-F_ = 8.1 Hz), 94.4 (d, *J*_C-F_ = 185.7 Hz, 2), 45.0 (*i*-Bu), 30.1 (*i*-Bu), 24.4 (d, *J*_C-F_ = 23.4 Hz, 3), 22.3 (*i*-Bu); HR MS: calcd for C_13_H_17_FO_2_Na (M+Na^+^) 247.1105, found 247.1105. Analytical data on racemic compound: Mp: 71–72 °C (CH_2_Cl_2_/hexane); IR (KBr): 2,970, 1,717, 1,612, 1,509, 1,112, 849 cm^−1^.

#### *Di(naphthalen-1-yl)methyl 2-Fluoro-2-phenylpropanoate* ((*Di(naphthalen-1-yl)methyl 2-Fluoro-2-phenylpropanoate*)-**4a**) [Table 1, Entry 4, and Table 2, Entry 1, 87% ee]


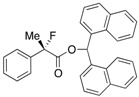


HPLC (CHIRALPAK AD-H, *i*-PrOH/hexane = 1/50, flow rate = 1.0 mL/min): *t*_R_ = 12.7 min (6.3%), *t*_R_ = 21.0 min (93.7%); ^1^H-NMR (CDCl_3_): δ 8.37 (s, 1H, 1'-H), 7.93–7.67 (m, 6H, Ar), 7.52–7.01 (m, 13H, Ar), 1.94 (d, *J*_H-F_ = 22.8 Hz, 3H, 2-Me); ^13^C-NMR (CDCl_3_): δ 169.9 (d, *J*_C-F_ = 27.9 Hz, 1), 138.6 (d, *J*_C-F_ = 22.7 Hz), 134.0, 133.9, 133.8, 133.7, 133.1, 130.9, 129.3, 129.1, 128.8, 128.75, 128.67, 128.4, 126.7, 126.6, 126.1, 125.9, 125.8, 125.6, 125.1, 125.1, 124.9 (d, *J*_C-F_ = 8.2 Hz), 123.3, 123.2, 94.7 (d, *J*_C-F_ = 186.4 Hz, 2), 72.6 (1'), 23.9 (d, *J*_C-F_ = 23.4 Hz, 3); HR MS: calcd for C_30_H_23_FO_2_Na (M+Na^+^) 457.1574, found 457.1584. Analytical data on racemic compound: Mp: 150–151 °C (CH_2_Cl_2_/hexane); IR (KBr): 3,065, 1,749, 1,600, 1,510, 1,123, 777, 698 cm^−1^.

#### *Di(naphthalen-1-yl)methyl 2-Fluoro-2-(o-tolyl)propanoate* ((*S*)-**4b**) [Table 2, Entry 2, 98% ee]


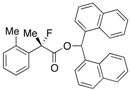


HPLC (CHIRALPAK AD-H, *i*-PrOH/hexane = 1/50, flow rate = 0.75 mL/min): *t*_R_ = 19.8 min (0.8%), *t*_R_ = 26.9 min (99.2%); ^1^H-NMR (CDCl_3_): δ 8.45 (s, 1H, 1’-H), 8.12–8.07 (m, 1H, Ar), 7.92–7.69 (m, 5H, Ar), 7.55–7.38 (m, 4H, Ar), 7.36–7.21 (m, 4H, Ar), 7.19–7.03 (m, 3H, Ar), 6.98-6.92 (m, 1H, Ar), 2.14 (s, 3H, *o*-Me), 1.95 (d, *J*_H-F_ = 22.8 Hz, 3H, 2-Me); ^13^C-NMR (CDCl_3_): δ 170.3 (d, *J*_C-F_ = 27.9 Hz, 1), 137.4, 136.0 (d, *J*_C-F_ = 20.5 Hz), 134.0, 134.0, 133.8, 133.7, 131.9 (d, *J*_C-F_ = 1.5 Hz), 131.3, 130.7, 129.4, 129.02, 128.97, 128.92, 128.7, 126.9, 126.8, 126.5, 126.4, 126.3, 126.0, 125.8, 125.7, 125.1 (d, *J*_C-F_ = 6.5 Hz), 125.0, 123.5, 123.2, 95.0 (d, *J*_C-F_ = 184.9 Hz, 2), 72.4 (1'), 23.8 (d, *J*_C-F_ = 25.0 Hz, 3), 20.3 (d, *J*_C-F_ = 5.9 Hz, *o*-Me); HR MS: calcd for C_31_H_25_FO_2_Na (M+Na^+^) 471.1736, found 471.1731. Analytical data on racemic compound: Mp: 142–144 °C (CH_2_Cl_2_/hexane); IR (KBr): 1,751, 1,600, 1,510, 1,124, 778, 733 cm^−1^.

#### *Di(naphthalen-1-yl)methyl 2-Fluoro-2-(m-tolyl)propanoate* ((*S*)-**4c**) [Table 2, Entry 3, 84% ee]


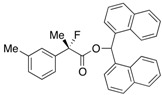


HPLC (CHIRALPAK AD-H, *i*-PrOH/hexane = 1/50, flow rate = 0.75 mL/min): *t*_R_ = 16.3 min (8.0%), *t*_R_ = 25.5 min (92.0%); ^1^H-NMR (CDCl_3_): δ 8.38 (s, 1H, 1'-H), 7.92–7.69 (m, 6H, Ar), 7.52–7.03 (m, 12H, Ar), 2.19 (s, 3H, *m*-Me), 1.93 (d, *J*_H-F_ = 22.4 Hz, 3H, 2-Me); ^13^C-NMR (CDCl_3_): δ 70.0 (d, *J*_C-F_ = 27.9 Hz, 1), 138.5 (d, *J*_C-F_ = 22.0 Hz), 138.2, 134.1, 134.0, 133.8, 133.7, 131.1, 130.9, 129.4 (d, *J*_C-F_ = 1.5 Hz), 129.2, 129.1, 128.8, 128.7, 128.3, 126.63, 126.57, 126.1, 125.85, 125.79, 125.7, 125.6 (d, *J*_C-F_ = 7.3 Hz), 125.1, 125.1, 123.3, 123.2, 121.9 (d, *J*_C-F_ = 8.1 Hz), 94.7 (d, *J*_C-F_ = 186.4 Hz, 2), 72.5 (1'), 23.9 (d, *J*_C-F_ = 23.5 Hz, 3), 21.3 (*m*-Me); HR MS: calcd for C_31_H_25_FO_2_Na (M+Na^+^) 471.1731, found 471.1713. Analytical data on racemic compound: Mp: 140–141 °C (CH_2_Cl_2_/hexane); IR (KBr): 1,744, 1,600, 1,510, 1,125, 800, 724, 700 cm^−1^.

#### *Di(naphthalen-1-yl)methyl 2-Fluoro-2-(p-tolyl)propanoate* ((*S*)-**4d**) [Table 2, Entry 5, 84% ee]


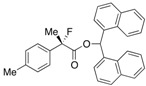


HPLC (CHIRALPAK AD-H, *i*-PrOH/hexane = 1/50, flow rate = 0.75 mL/min): *t*_R_ = 20.5 min (8.1%), *t*_R_ = 38.0 min (91.9%); ^1^H-NMR (CDCl_3_): δ 8.36 (s, 1H, 1'-H), 7.90–7.69 (m, 6H, Ar), 7.50–7.19 (m, 8H, Ar), 7.16–7.01 (m, 4H, Ar), 2.33 (s, 3H, *p*-Me), 1.92 (d, *J*_H-F_ = 22.4 Hz, 3H, 2-Me); ^13^C-NMR (CDCl_3_): δ 170.1 (d, *J*_C-F_ = 27.9 Hz, 1), 138.5 (d, *J*_C-F_ = 1.5 Hz), 135.7 (d, *J*_C-F_ = 22.7 Hz), 134.1, 134.0, 133.8, 133.7, 131.1, 130.9, 129.2, 129.1, 129.0, 129.0, 128.8, 128.7, 126.6, 126.5, 126.1, 125.8, 125.7, 125.6, 125.1, 125.1, 124.9 (d, *J*_C-F_ = 8.1 Hz), 123.3 (d, *J*_C-F_ = 7.3 Hz), 94.6 (d, *J*_C-F_ = 185.7 Hz, 2), 72.5 (1'), 23.8 (d, *J*_C-F_ = 23.5 Hz, 3), 21.1 (*p*-Me); HR MS: calcd for C_31_H_25_FO_2_Na (M+Na^+^) 471.1731, found 471.1720. Analytical data on racemic compound: Mp: 133–134 °C (CH_2_Cl_2_/hexane); IR (KBr): 1,749, 1,600, 1,511, 1,118, 804, 777, 734 cm^−1^.

#### *Di(naphthalen-1-yl)methyl 2-(2-Chlorophenyl)-2-fluoropropanoate* ((*S*)-**4e**) [Table 2, Entry 6, 91% ee]


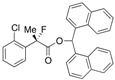


HPLC (CHIRALPAK AD-H, *i*-PrOH/hexane = 1/50, flow rate = 0.75 mL/min): *t*_R_ = 23.8 min (4.6%), *t*_R_ = 34.1 min (95.4%); ^1^H-NMR (CDCl_3_): δ 8.49 (s, 1H, 1'-H), 8.09–7.75 (m, 6H, Ar), 7.57–7.19 (m, 12H, Ar), 1.97 (d, *J*_H-F_ = 23.2 Hz, 3H, 2-Me); ^13^C-NMR (CDCl_3_): δ 68.6 (d, *J*_C-F_ = 25.0 Hz, 1), 136.7 (d, *J*_C-F_ = 22.0 Hz), 134.1, 133.9, 133.8, 133.8, 131.9 (d, *J*_C-F_ = 3.6 Hz), 131.1, 131.0, 130.6, 130.0, 129.24, 129.17, 128.78, 128.76, 127.0 (d, *J*_C-F_ = 11.8 Hz), 126.8, 126.59, 126.56, 126.54, 126.1, 125.8, 125.8, 125.08, 125.05, 123.6, 123.6, 94.5 (d, *J*_C-F_ = 183.4 Hz, 2), 72.9 (1'), 23.1 (d, *J*_C-F_ = 24.2 Hz, 3); HR MS: calcd for C_30_H_22_ClFO_2_Na (M+Na^+^) 491.1185.0402, found 491.1166. Analytical data on racemic compound: Mp: 171–175 °C (CH_2_Cl_2_/hexane); IR (KBr): 1,745, 1,599, 1,511, 1,257, 1,122, 804, 775, 756 cm^−1^.

#### *Di(naphthalen-1-yl)methyl 2-(3-Chlorophenyl)-2-fluoropropanoate* ((*S*)-**4f**) [Table 2, Entry 8, 76% ee]


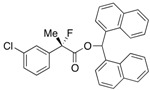


HPLC (CHIRALPAK AD-H, *i*-PrOH/hexane = 1/50, flow rate = 0.75 mL/min): *t*_R_ = 15.2 min (12.0%), *t*_R_ = 23.4 min (88.0%); ^1^H-NMR (CDCl_3_): δ 8.37 (s, 1H, 1'-H), 7.95-7.71 (m, 6H, Ar), 7.54–7.08 (m, 12H, Ar), 1.92 (d, *J*_H-F_ = 22.0 Hz, 3H, 2-Me); ^13^C-NMR (CDCl_3_): δ 69.3 (d, *J*_C-F_ = 27.2 Hz, 1), 140.6 (d, *J*_C-F_ = 23.4 Hz), 134.5 (d, *J*_C-F_ = 1.4 Hz), 133.81, 133.79, 133.74, 131.0, 130.9, 129.7, 129.4, 129.3, 128.86, 128.85, 128.84, 128.82, 126.7, 126.6, 126.04, 125.91, 125.87, 125.7, 125.2 (d, *J*_C-F_ = 8.8 Hz), 125.12, 125.10, 123.12, 123.10, 123.08 (d, *J*_C-F_ = 8.1 Hz), 94.2 (d, *J*_C-F_ = 188.6 Hz, 2), 73.0 (1'), 24.1 (d, *J*_C-F_ = 23.4 Hz, 3); HR MS: calcd for C_30_H_22_ClFO_2_Na (M+Na^+^) 491.1185, found 491.1194. Analytical data on racemic compound: Mp: 113–115 °C (CH_2_Cl_2_/hexane); IR (KBr): 1,748, 1,598, 1,510, 1,127, 798, 777, 712 cm^−1^.

#### *Di(naphthalen-1-yl)methyl 2-(3-Chlorophenyl)-2-fluoropropanoate* ((*S*)-**4g**) [Table 2, Entry 9, 75% ee]


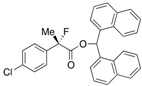


HPLC (CHIRALPAK AD-H, *i*-PrOH/hexane = 1/50, flow rate = 0.75 mL/min): *t*_R_ = 19.3 min (12.3%), *t*_R_ = 40.0 min (87.7%); ^1^H-NMR (CDCl_3_): δ 8.34 (s, 1H, 1'-H), 7.93–7.74 (m, 5H, Ar), 7.75–7.64 (m, 1H, Ar), 7.52–7.06 (m, 12H, Ar), 1.95 (d, *J*_H-F_ = 22.0 Hz, 3H, 2-Me); ^13^C-NMR (CDCl3): δ 69.5 (d, *J*_C-F_ = 27.9 Hz, 1), 137.1 (d, *J*_C-F_ = 22.7 Hz), 134.8 (d, *J*_C-F_ = 2.2 Hz), 133.81, 133.76, 131.0, 130.9, 129.4, 129.3, 129.0, 128.9, 128.8, 128.5, 128.5, 126.7, 126.6, 126.4 (d, *J*_C-F_ = 8.1 Hz), 126.0, 125.91, 125.86, 125.6, 125.1, 125.1, 123.13, 123.12, 94.3 (d, *J*_C-F_ = 187.8 Hz, 2), 73.0 (1'), 23.0 (d, *J*_C-F_ = 23.4 Hz, 3); HR MS: calcd for C_30_H_22_ClFO_2_K (M+K^+^) 507.0929, found 507.0940. Analytical data on racemic compound: Mp: 129–131 °C (CH_2_Cl_2_/hexane); IR (KBr): 1,749, 1,510, 1,490, 1,121, 1,093, 838, 778 cm^−^^1^.

#### *Di(naphthalen-1-yl)methyl 2-Fluoro-2-(2-fluorophenyl)propanoate* ((*S*)-**4h**) [Table 2, Entry 10, 60% ee]


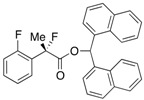


HPLC (CHIRALPAK AD-H, *i*-PrOH/hexane = 1/50, flow rate = 0.75 mL/min): *t*_R_ = 20.2 min (20.2%), *t*_R_ = 24.9 min (79.8%); ^1^H-NMR (CDCl_3_): δ 8.45 (s, 1H, 1'-H), 8.10–7.78 (m, 6H, Ar), 7.53–7.19 (m, 10H, Ar), 7.12–7.04 (m, 1H, Ar), 6.99–6.89 (m, 1H, Ar), 1.96 (d, *J*_H-F_ = 22.8 Hz, 3H, 2-Me); ^13^C-NMR (CDCl3): δ 169.0 (d, *J*_C-F_ = 26.5 Hz, 1), 158.4 (dd, *J*_C-F_ = 254.5, 4.3 Hz), 134.0 (dd, *J*_C-F_ = 8.9, 1.5 Hz), 133.80, 133.78, 131.1, 131.0, 130.8, 130.7, 129.3, 129.3, 129.2, 129.2, 128.8, 128.8, 126.9 (dd, *J*_C-F_ = 10.0, 3.3 Hz), 126.64, 126.59, 126.42 (dd, *J*_C-F_ = 19.1, 14.0 Hz), 126.2, 125.84, 125.80, 125.1, 125.1, 124.1 (d, *J*_C-F_ = 3.7 Hz), 123.3, 116.1 (d, *J*_C-F_ = 22.0 Hz), 92.8 (d, *J*_C-F_ = 185.6 Hz, 2), 72.8 (1'), 23.1 (dd, *J*_C-F_ = 24.3, 2.9 Hz, 3); HR MS: calcd for C_30_H_22_F_2_O_2_Na (M+Na^+^) 475.1480, found 475.1485. Analytical data on racemic compound: Mp: 144–146 °C (CH_2_Cl_2_/hexane); IR (KBr): 1,738, 1,618, 1,599, 1,585, 1,120, 1,096, 774, 759 cm^−1^.

#### *Di(naphthalen-1-yl)methyl 2-(2-Bromophenyl)-2-fluoropropanoate* ((*S*)-**4i**) [Table 2, Entry 11, 94% ee]


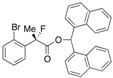


HPLC (CHIRALPAK AD-H, *i*-PrOH/hexane = 1/50, flow rate = 0.75 mL/min): *t*_R_ = 27.4 min (2.8%), *t*_R_ = 38.4 min (97.2%); ^1^H-NMR (CDCl_3_): δ 8.50 (s, 1H, 1'-H), 8.10–8.03 (m, 1H, Ar), 8.01–7.77 (m, 5H, Ar), 7.55–7.22 (m, 11H, Ar), 7.18–7.11 (m, 1H, Ar), 1.98 (d, *J*_H-F_ = 23.2 Hz, 3H, 2-Me); ^13^C-NMR (CDCl3): δ 168.5 (d, *J*_C-F_ = 25.7 Hz, 1), 138.2 (d, *J*_C-F_ = 21.2 Hz), 134.14, 134.08, 133.9, 133.8, 133.8, 131.1, 131.0, 130.1, 129.24, 129.16, 128.8, 128.8, 127.4, 127.31, 127.30, 126.6, 126.6 (d, *J*_C-F_ = 10.2 Hz), 126.2, 125.81, 125.79, 125.09, 125.07, 123.6, 123.6, 120.9 (d, *J*_C-F_ = 3.6 Hz), 95.4 (d, *J*_C-F_ = 183.3 Hz, 2), 72.9 (1'), 23.3 (d, *J*_C-F_ = 24.9 Hz, 3); HR MS: calcd for C_30_H_22_BrFO_2_Na (M+Na^+^) 535.0679, found 535.0684. Analytical data on racemic compound: Mp: 181–183 °C (CH_2_Cl_2_/hexane); IR (KBr): 1,744, 1,598, 1,510, 1,121, 796, 774, 756, 639 cm^−1^.

#### *Di(naphthalen-1-yl)methyl 2-Fluoro-2-(2-methoxyphenyl)propanoate* ((*S*)-**4j**) [Table 2, Entry 12, 89% ee]


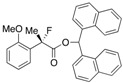


HPLC (CHIRALPAK AD-H, *i*-PrOH/hexane = 1/9, flow rate = 0.75 mL/min): *t*_R_ = 14.3 min (5.6%), *t*_R_ = 16.9 min (94.4%); ^1^H-NMR (CDCl_3_): δ 8.52 (s, 1H, 1'-H), 8.13–8.06 (m, 1H, Ar), 8.00–7.92 (m, 1H, Ar), 7.88–7.72 (m, 4H, Ar), 7.50–7.19 (m, 10H, Ar), 6.96–6.86 (m, 1H, Ar), 6.65-6.57 (m, 1H, Ar), 3.04 (s, 3H, OMe), 1.95 (d, *J*_H-F_ = 22.8 Hz, 3H, 2-Me); ^13^C-NMR (CDCl3): δ 169.8 (d, *J*_C-F_ = 26.4 Hz, 1), 156.1 (d, *J*_C-F_ = 3.6 Hz), 134.6, 134.5, 133.8, 133.8, 131.1, 131.0, 130.1 (d, *J*_C-F_ = 1.4 Hz), 129.0, 128.8, 128.7, 126.6, 126.5, 126.5, 126.1, 126.0 (d, *J*_C-F_ = 9.6 Hz), 125.74, 125.69, 125.1, 125.1, 125.0, 125.0, 123.7, 123.6, 120.2, 110.6, 93.3 (d, *J*_C-F_ = 180.4 Hz, 2), 72.0 (1'), 54.5 (OMe), 22.8 (d, *J*_C-F_ = 25.0 Hz, 3); HR MS: calcd for C_31_H_25_FO_3_Na (M+Na^+^) 487.1680, found 487.1685. Analytical data on racemic compound: Mp: 152–154 °C (CH_2_Cl_2_/hexane); IR (KBr): 1,734, 1,601, 1,589, 1,510, 1,495, 1,119, 801, 788, 776, 749 cm^−1^.

#### *Di(naphthalen-1-yl)methyl 2-Fluoro-2-(4-isobutylphenyl)propanoate* ((*S*)-**4k**) [Table 2, Entry 13, 88% ee]


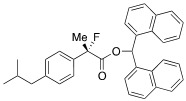


HPLC (CHIRALPAK AD-H, *i*-PrOH/hexane = 1/50, flow rate = 0.75 mL/min): *t*_R_ = 20.9 min (5.8%), *t*_R_ = 29.4 min (94.2%); ^1^H-NMR (CDCl_3_): δ 8.38 (s, 1H, 1'-H), 7.95–7.68 (m, 6H, Ar), 7.51–7.19 (m, 8H, Ar), 7.14–7.00 (m, 4H, Ar), 2.47 (d, *J*_H-H_ = 7.2 Hz, 2H, *i*-Bu), 1.92 (d, *J*_H-F_ = 22.4 Hz, 3H, 2-Me), 1.92–1.79 (m, 1H, *i*-Bu), 0.91 (d, *J*_H-H_ = 6.4 Hz, 6H, *i*-Bu); ^13^C-NMR (CDCl3): δ 170.1 (d, *J*_C-F_ = 28.6 Hz, 1), 142.3 (d, *J*_C-F_ = 1.5 Hz), 135.9 (d, *J*_C-F_ = 22.7 Hz), 134.1, 134.0, 133.8, 133.7, 131.1, 130.9, 129.3, 129.10, 129.06, 128.8, 128.7, 126.7, 126.5, 126.2, 125.9, 125.8, 125.5, 125.09, 125.07, 124.8 (d, *J*_C-F_ = 7.3 Hz), 123.3, 123.2, 94.6 (d, *J*_C-F_ = 185.7 Hz, 2), 72.4 (1'), 45.0 (*i*-Bu), 30.1 (*i*-Bu), 23.9 (d, *J*_C-F_ = 23.5 Hz, 3), 22.4 (*i*-Bu); HR MS: calcd for C_34_H_31_FO_2_K (M+K+) 529.1945, found 529.1949. Analytical data on racemic compound: IR (KBr): 1,755, 1,599, 1,511, 1,118, 849, 793, 777 cm^−1^. 

### 3.5. Identification of the Absolute Configuration by Hydrolysis of the Chiral Esters (R)-***4k***


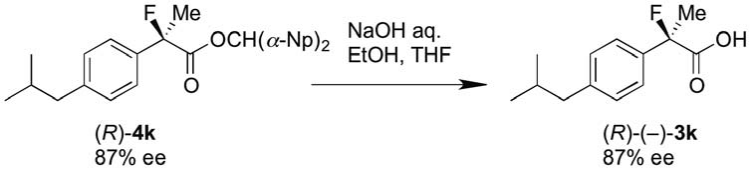


To a solution of the resolved di(naphthalen-1-yl)methyl 2-fluoro-2-(4-isobutylphenyl)-propanoate [(*R*)-**4k**, obtained in analogy with procedure 3.4. by using (–)-BTM, 83.9 mg, 0.171 mmol, 87% ee] in ethanol (3 mL) and THF (2 mL) was added aqueous sodium hydroxide (1 M, 0.86 mL, 0.86 mmol). The reaction mixture was stirred at room temperature for 1 h. The reaction mixture was acidified with 1 M hydrochloric acid (10 mL) and extracted with ethyl acetate. The organic layer was dried over sodium sulfate. After filtration of the mixture and evaporation of the solvent, the crude product was purified by preparative thin layer chromatography on silica (ethyl acetate/hexane/formic acid = 25/75/2) to afford the optically active 2-fluoroibuprofen (**3k**) (30.0 mg, 76% yield, 87% ee) as a white solid. Absolute configuration of this product ([α]_D_^27^ −26.9° (*c* 1.2, EtOH)) was determined as *R* by reference to the data of (*R*)-(–)-**3k** ([α]_D_^24^ −22.6° (*c* 1.2, EtOH)) [4].

## 4. Conclusions

In summary, we have developed an effective kinetic resolution system to provide optically active 2-aryl-2-fluoropropanoic acids and the corresponding esters. By screening the reaction media, it was found that diethyl ether was a most suitable solvent for the conversion of carboxylic acids into the corresponding esters. Investigation of a series of substituents on the 2-phenyl groups showed the preference of *ortho*-substitution, except for the fluoro and methoxy groups. This procedure was successfully applied for the kinetic resolution of racemic 2-fluoroibuprofen to provide the corresponding chiral ester of biologically important (*R*)-(–)-2-fluoroibuprofen with high enantiomeric excess. In addition, we successfully disclose the reaction mechanism resulting in the high enantioselectivity using theoretical calculations. This protocol presents a practical approach to afford the medicinally important fluorinated chiral derivatives in terms of experimental convenience and cost. Further investigation will be focused on the application for the syntheses of the chiral *α*-fluorinated drugs having quaternary carbons at the *α*-positions in the 2-aryl-2-fluoropropanoic acid structure.

## Supplementary Materials

Supplementary materials can be accessed at: http://www.mdpi.com/1420-3049/17/6/7356/s1.
