# Supplementary material for: A New Method for Production of Chiral 2-Aryl-2-fluoropropanoic Acids Using an Effective Kinetic Resolution of Racemic 2-Aryl-2-fluoropropanoic Acids
Source: Molecules. 2012 Jun 14;17(6):7356–78. doi: 10.3390/molecules17067356 (PMC6268090; doi:10.3390/molecules17067356)
Supplement: Supplementary file 1 [file molecules-17-07356-s001.pdf]

Molecules **2012**, *17*, 7356-7378; doi:10.3390/molecules17067356

OPEN ACCESS

molecules

ISSN 1420-3049

www.mdpi.com/journal/molecules

Supporting Information

A New Method for Production of Chiral 2-Aryl-2-fluoropropanoic Acids Using an Effective Kinetic Resolution of Racemic 2-Aryl-2-fluoropropanoic Acids

Atsushi Tengeiji and Isamu Shiina *

Department of Applied Chemistry, Faculty of Science, Tokyo University of Science, 1-3 Kagurazaka, Shinjuku-ku, Tokyo 162-8601, Japan

* Author to whom correspondence should be addressed; E-Mail: shiina@rs.kagu.tus.ac.jp; Fax: +81-3-3260-5609.

Received: 2 May 2012; in revised form: 4 June 2012 / Accepted: 8 June 2012 / Published: 14 June 2012

Supplementary Material

S2-7 Cartesian Coordinates of (*S*)-**ts** and (*R*)-**ts**

S8-73 ^{1}H and ^{13}C-NMR Spectroscopic Data of Compounds

All calculations were performed with the program package <u>*Spartan '10* 1.1.0</u> of Wavefunction Inc. All structures were optimized and subjected to frequency analysis with the B3LYP/6-31G* method, followed by single point B3LYP/6-31G* calculation.

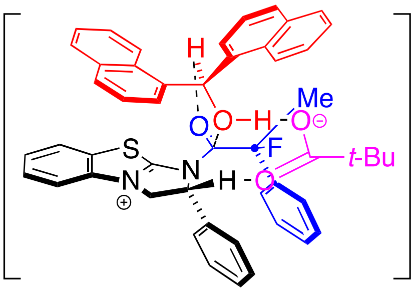

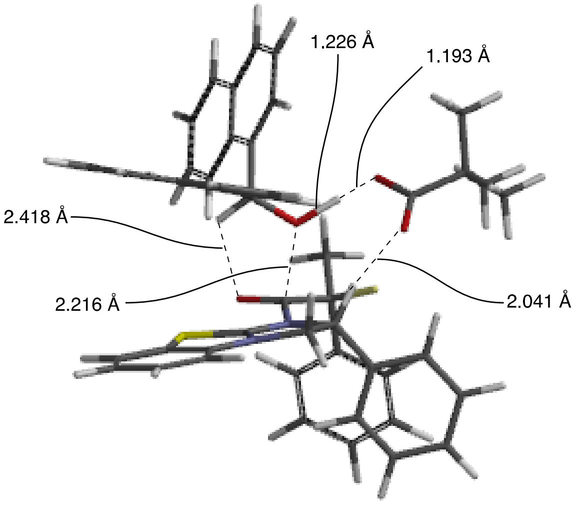

Transition Structure (*S*)-**ts**

E(B3LYP/6-31G*) = −2840.88644 au

v_{ts} = 593*i* cm^{-1}

Cartesian Coordinates (Angstroms)

Atomic Number	X	Y	Z
6	-0.955845692	0.042678344	-1.344920874
6	-2.043558984	-1.044714559	-1.378096355
8	-0.224209604	0.230984102	-2.311526818
7	-1.110299970	1.163161400	-0.439600623
6	-0.269140419	2.191191250	-0.579184484
7	-0.215307135	2.988052744	0.492811473
6	-0.988443005	2.427409235	1.607086796
6	-1.726854186	1.215230178	0.941511935
6	-3.227572910	1.419982487	0.937703733
6	-3.995033255	0.706434573	1.866621776
6	-5.368827286	0.933756912	1.961478814
6	-5.987042590	1.866574025	1.126882313
6	-5.224785075	2.577814842	0.197546044
6	-3.849951874	2.360238463	0.108186716
1	-3.267694677	2.911615630	-0.626822728
1	-5.700062799	3.300037907	-0.460671936
1	-7.057905561	2.038184141	1.197886072
1	-5.956717588	0.375439459	2.685315591
1	-3.503082734	-0.021976670	2.506159966
1	-1.459366925	0.295431827	1.466240357
1	-0.306175097	2.110519512	2.400322135
1	-1.692161013	3.168932688	1.991728573

8	0.313928719	-1.000223625	0.141401688
6	1.686705734	-0.863308801	-0.114828066
6	2.377186846	0.050858781	0.909789730
6	3.610452527	0.717119128	0.599633960
6	4.189952810	1.605864132	1.568756504
6	3.551348660	1.782028681	2.825600851
6	2.386623344	1.110090342	3.110116768
6	1.794738098	0.255941990	2.146717840
1	0.863152415	-0.248490862	2.385097539
1	1.909307845	1.225230557	4.080895458
1	4.007628664	2.444848818	3.557715047
1	1.775658445	-0.355946033	-1.085339947
1	-0.029838422	-1.840122721	0.965898826
8	-0.433182784	-2.691152855	1.698113845
6	-1.171580944	-2.272134409	2.674979202
6	-1.837674374	-3.406438486	3.486009184
8	-1.354453378	-1.077125936	2.973293417
16	0.762728499	2.688499321	-1.875513533
6	0.641322461	4.079445281	0.373525556
6	1.256335512	4.093488551	-0.890690990
6	2.155317694	5.100586027	-1.228484061
1	2.639573277	5.112649546	-2.199999788
6	0.907763235	5.064524193	1.323207783
1	0.434470551	5.040750267	2.299434766
6	1.806835358	6.071449836	0.975865989
1	2.034299266	6.850314276	1.697080947
6	2.421737537	6.092262996	-0.283285971
1	3.119237683	6.887027579	-0.528516037
6	2.381969329	-2.231735077	-0.232713277
6	3.609757439	-4.784752485	-0.339440766
6	2.839505867	-2.864789734	0.907105821
6	2.521895944	-2.895126917	-1.499689673
6	3.159358345	-4.183946330	-1.543843495
6	3.445352532	-4.139990838	0.862268181
1	2.735143241	-2.368305700	1.866361999
1	3.787324786	-4.600971006	1.785265842
1	4.085653096	-5.761641099	-0.386716672
6	-1.492376774	-2.374760514	-1.883330203
1	-0.687707335	-2.706780906	-1.228456411
1	-1.113156785	-2.272860785	-2.902115994
6	-3.217255062	-0.563298489	-2.244556247
6	-5.391212378	0.167109293	-3.862281554
6	-3.005190480	-0.094510867	-3.548321915
6	-4.525889724	-0.668291616	-1.760923966
6	-5.605186208	-0.303864130	-2.567103516
6	-4.086848961	0.270221036	-4.349455200
1	-1.993423890	-0.010386886	-3.931645677
1	-4.696632235	-1.025749295	-0.752755178
1	-6.615604965	-0.388297149	-2.175839723
1	-3.907519059	0.633043470	-5.358295978
1	-6.232924432	0.450223952	-4.488889393

6	-0.729222683	-4.324870580	4.042262420
1	-1.169219577	-5.167899636	4.590277561
1	-0.077597514	-3.779021821	4.736434874
1	-0.109985452	-4.718736267	3.231635453
6	-2.742926878	-4.208025533	2.525814873
1	-3.238482934	-5.028683430	3.059939193
1	-2.156494719	-4.625122981	1.702707123
1	-3.521855915	-3.566333866	2.095499696
6	-2.670592238	-2.828374236	4.639695876
1	-3.118493889	-3.639297553	5.227889432
1	-3.481555543	-2.193940440	4.265906219
1	-2.053428527	-2.216716730	5.305446309
6	2.067539192	-2.342103104	-2.732345257
1	1.543902526	-1.393562948	-2.736146022
6	3.328230595	-4.839477731	-2.793257327
1	3.816435072	-5.811671024	-2.803530077
6	2.886714454	-4.267190322	-3.963435532
1	3.022046753	-4.780478394	-4.911934182
6	2.245605341	-3.007463084	-3.926136006
1	1.883113043	-2.560492388	-4.848657471
6	4.295700021	0.542004358	-0.637279288
1	3.901281010	-0.148265829	-1.374965113
6	5.396913657	2.286364872	1.254796950
1	5.822254097	2.956509767	1.998935376
6	6.024296074	2.100650694	0.044417168
1	6.950232258	2.624275573	-0.179283983
6	5.467197684	1.215201211	-0.907717294
1	5.971855826	1.058689544	-1.857674534
1	-2.306359717	-3.105319692	-1.881333244
9	-2.517325816	-1.248063568	-0.077379298

Requested basis set is 6-31G(d)

There are 323 shells and 964 basis functions

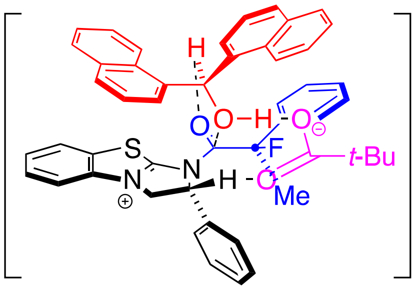

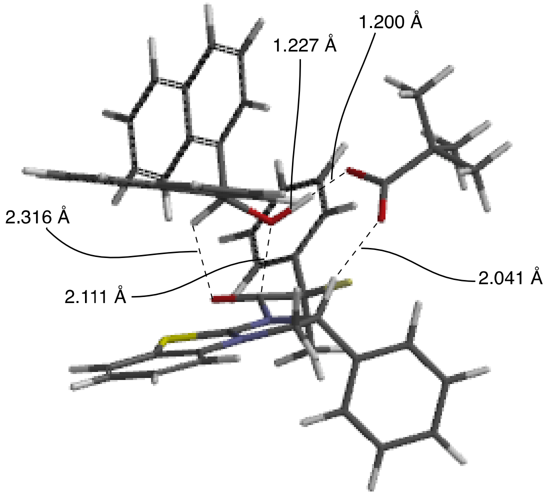

Transition Structure (*R*)-**ts**
E(B3LYP/6-31G*) = −2840.88100 au
◘ ν_{ts} = 702i cm^{-1}

Cartesian Coordinates (Angstroms)

Atomic Number X Y Z

6	-0.029549745	0.790721707	1.699671893
6	1.034018959	1.887444950	1.913879897
8	-0.104673710	-0.173542061	2.455924204
7	-1.297711426	1.192433515	1.083599521
6	-2.320279460	0.341497234	1.163998559
7	-3.291659157	0.584702163	0.274944072
6	-2.892072644	1.637100646	-0.666127473
6	-1.566341700	2.180146305	-0.030476379
6	-1.726834139	3.612267754	0.441248315
6	-1.075579678	4.627622853	-0.268062601
6	-1.268059222	5.964263470	0.084811649
6	-2.105597465	6.298525857	1.149924257
6	-2.758402154	5.289034880	1.861461312
6	-2.574017849	3.953001711	1.504417305
1	-3.084644541	3.174079936	2.067785013
1	-3.410611056	5.540888513	2.693551262
1	-2.249403577	7.339561447	1.426395881
1	-0.756001329	6.745084335	-0.471193195
1	-0.425389310	4.356089151	-1.094796013
1	-0.754318641	2.102077411	-0.756454348
1	-2.728305957	1.198585637	-1.653819107
1	-3.661528725	2.410093297	-0.723757419
8	0.739344487	0.095661768	-0.139782009
6	0.608751562	-1.301046219	-0.261152629
6	-0.485413960	-1.704759679	-1.265500114
6	-1.104146083	-3.000239348	-1.199832586
6	-2.144836667	-3.329760818	-2.134572494
6	-2.524208508	-2.380748262	-3.121154417
6	-1.905872688	-1.155024825	-3.175236478
6	-0.897155839	-0.814801848	-2.239860519
1	-0.441721886	0.168742860	-2.293718303
1	-2.181986607	-0.434613096	-3.942314183
1	-3.300826384	-2.646837831	-3.834965390
1	0.270589805	-1.658101509	0.718916266
1	1.406752057	0.681394492	-0.986471233
8	2.094741019	1.249495437	-1.788830631
6	1.509376456	2.145799042	-2.519995928
6	2.449379343	2.846949195	-3.528240219
8	0.299166926	2.429206554	-2.473268538
16	-2.635923276	-1.018939096	2.187928740
6	-4.379499232	-0.281826649	0.336614789
6	-4.205765733	-1.228458590	1.361016179
6	-5.181744656	-2.189760231	1.606762713
1	-5.051355367	-2.927736028	2.392159195
6	-5.521676367	-0.273681176	-0.462060153
1	-5.641805619	0.455987870	-1.256260734
6	-6.495473243	-1.238384280	-0.207894724
1	-7.394581883	-1.256749194	-0.816029949
6	-6.330718822	-2.182952186	0.813915804
1	-7.103908620	-2.923974351	0.991047803

6	1.946393517	-1.991243241	-0.583952989
6	4.373409386	-3.263584637	-1.307425691
6	2.409481363	-1.983831842	-1.885851556
6	2.729302833	-2.643142868	0.429056122
6	3.946855789	-3.306293626	0.045234383
6	3.624347732	-2.603850343	-2.251617775
1	1.821189638	-1.492463370	-2.653485914
1	3.951697880	-2.567578470	-3.287490907
1	5.300451798	-3.763113054	-1.580185836
6	2.939364545	1.782190631	-4.534294294
1	3.611853274	2.236735716	-5.272562859
1	2.097295726	1.336738474	-5.078288139
1	3.476400788	0.979234612	-4.021204764
6	3.658989598	3.431654456	-2.771518511
1	4.336017593	3.937992045	-3.471295795
1	4.215818989	2.643684163	-2.257510586
1	3.342257428	4.167712674	-2.021943087
6	1.700912613	3.964047382	-4.270040255
1	2.359954065	4.431938383	-5.011921087
1	1.359537043	4.742270103	-3.578638861
1	0.817169827	3.575023019	-4.784147555
6	0.586241340	2.785023236	3.079525934
1	0.531125647	2.210580281	4.006791125
1	-0.388249140	3.235367201	2.879493055
6	2.427150840	1.323570975	2.162205992
6	5.037289192	0.426665879	2.671746504
6	3.438180153	1.517199641	1.215210080
6	2.731703080	0.667636640	3.362477656
6	4.028477071	0.220453952	3.613207498
6	4.734879398	1.071513856	1.472679430
1	3.205582838	1.992705732	0.271506805
1	1.954235343	0.484754705	4.096752410
1	4.246927422	-0.293706045	4.545476833
1	5.508254326	1.224174470	0.724578793
1	6.047729323	0.077555189	2.867211211
6	-0.737835840	-3.987619136	-0.240639693
1	0.062732142	-3.785157797	0.461485706
6	-2.772267332	-4.602408090	-2.060946088
1	-3.557004047	-4.834825603	-2.777967324
6	-1.366048095	-5.213364029	-0.197740684
1	-1.060415401	-5.948373501	0.542471455
6	-2.396338973	-5.527177395	-1.114044991
1	-2.882394313	-6.498499527	-1.072100098
6	4.707417781	-3.996146609	1.027464733
1	5.621957021	-4.496155617	0.715300713
6	2.362896509	-2.682776006	1.805803470
1	1.489198988	-2.139006538	2.145430093
6	3.123960098	-3.363058813	2.731265386
1	2.819428210	-3.369563802	3.774854000
6	4.304679068	-4.035677034	2.341902688
1	4.894510902	-4.571460681	3.081363473
1	1.324392308	3.582148118	3.203074039
9	1.063918673	2.697151233	0.772207398

Requested basis set is 6-31G(d)

There are 323 shells and 964 basis functions

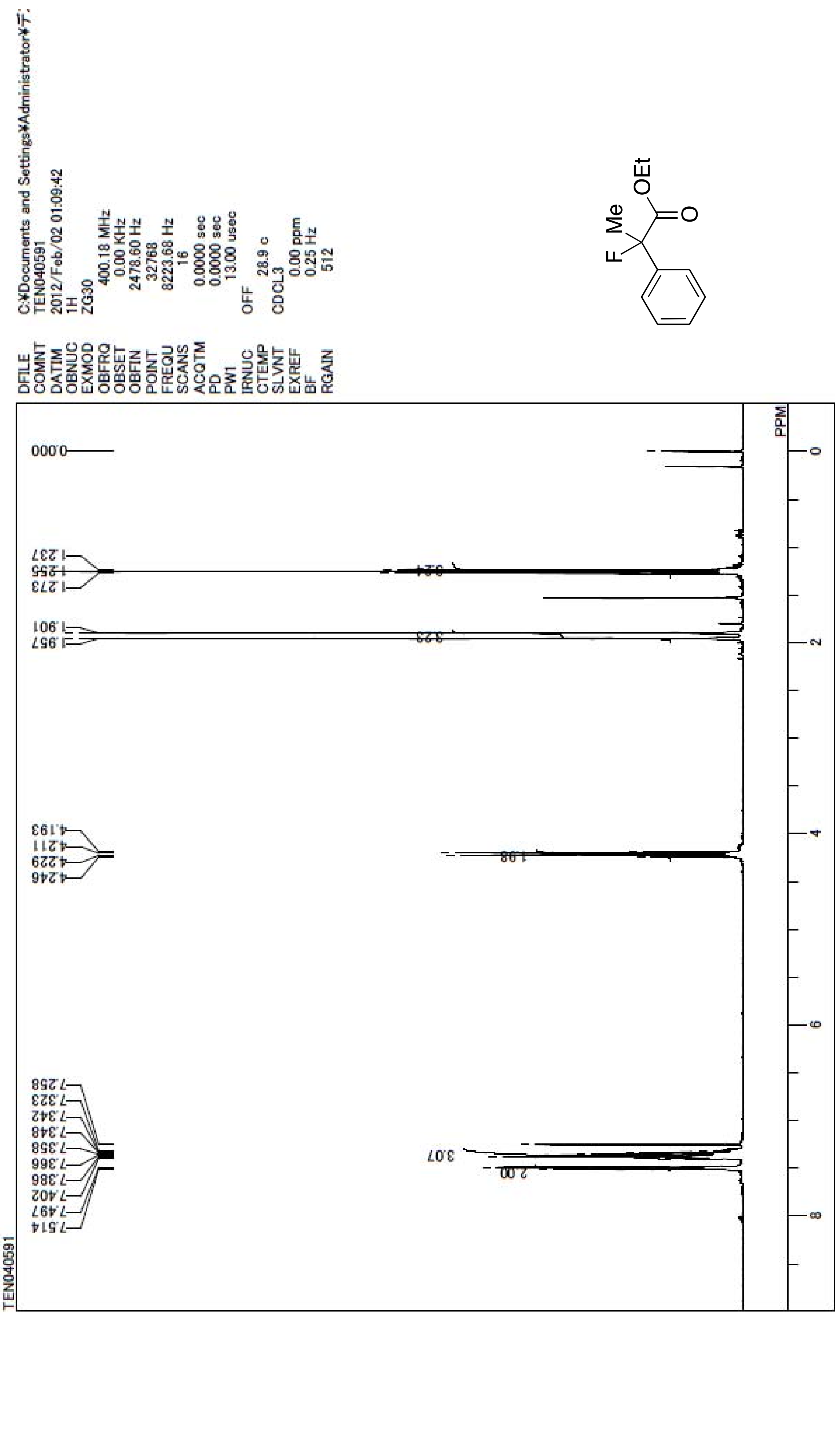

DFILE C:¥Documents and Settings¥Administrator¥デ:
COMNT TEN040591
DATIM 2012/Feb/02 01:09:42
OBNUC 1H
EXMOD ZG30
OBFRQ 400.18 MHz
OBSET 0.00 KHz
OBFIN 2478.60 Hz
POINT 32768
FREQU 8223.68 Hz
SCANS 16
ACQTM 0.0000 sec
PD 0.0000 sec
PW1 13.00 usec
IRNUC OFF
CTEMP 28.9 c
SLVNT CDCL3
EXREF 0.00 ppm
BF 0.25 Hz
RGAIN 512
F
Me
OEt
O
TEN040591
0.000
1.237
1.255
1.273
1.901
1.957
4.193
4.211
4.229
4.246
7.258
7.323
7.342
7.348
7.358
7.366
7.386
7.402
7.497
7.514
3.24
3.23
1.98
3.07
2.00
PPM
0
2
4
6
8

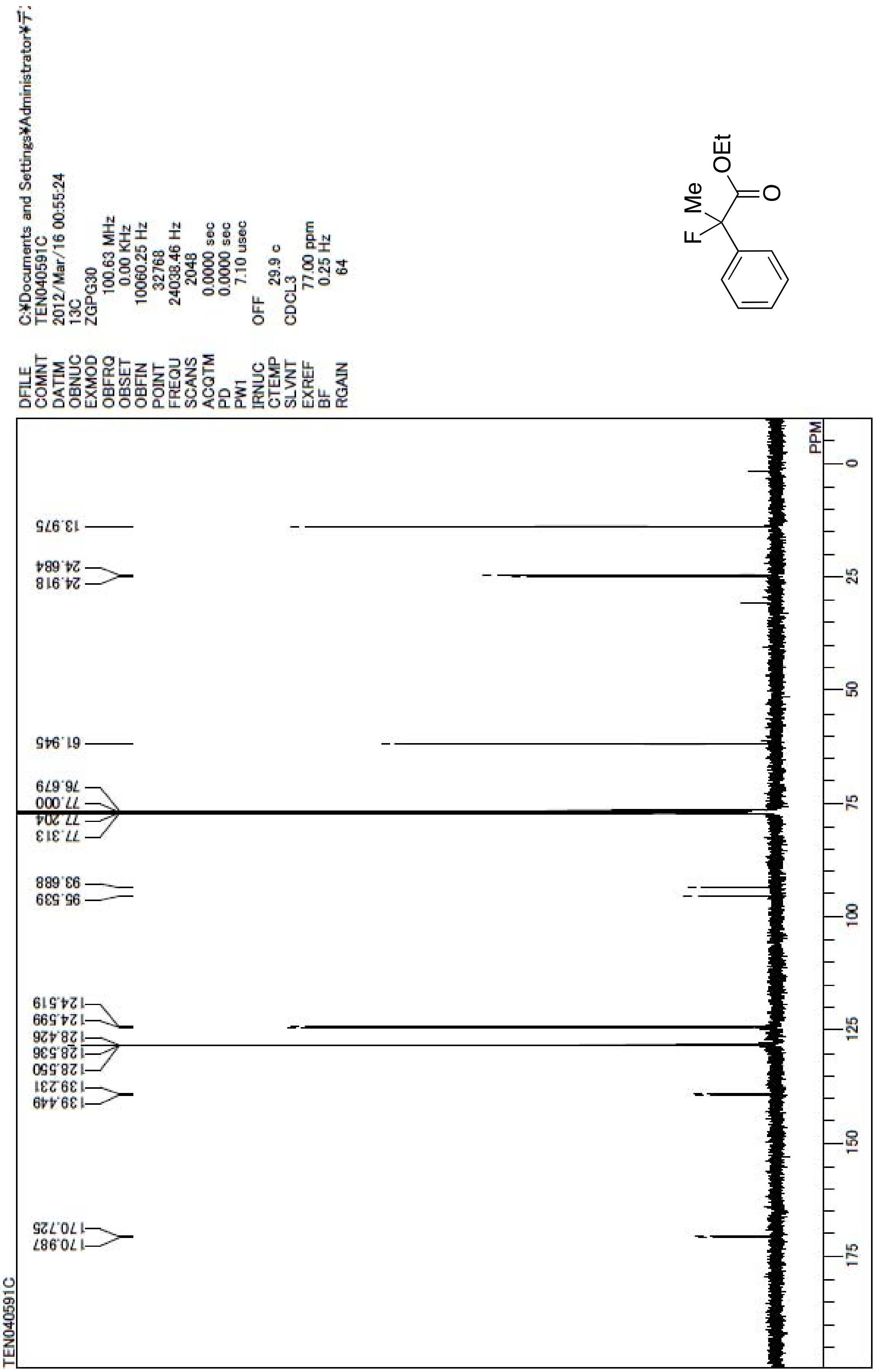
DFILE C:¥Documents and Settings¥Administrator¥デ:
COMNT TEN040591C
DATIM 2012/Mar/16 00:55:24
OBNUC 13C
EXMOD ZGPG30
OBFRQ 100.63 MHz
OBSET 0.00 KHz
OBFIN 10060.25 Hz
POINT 32768
FREQU 24038.46 Hz
SCANS 2048
ACQTM 0.0000 sec
PD 0.0000 sec
PW1 7.10 usec
IRNUC OFF
CTEMP 29.9 c
SLVNT CDCL3
EXREF 77.00 ppm
BF 0.25 Hz
RGAIN 64
TEN040591C
13.975
24.684
24.918
61.945
76.679
77.000
77.204
77.313
93.688
95.539
124.519
124.599
128.426
128.536
128.550
139.231
139.449
170.725
170.987
PPM
F
Me
OEt
O

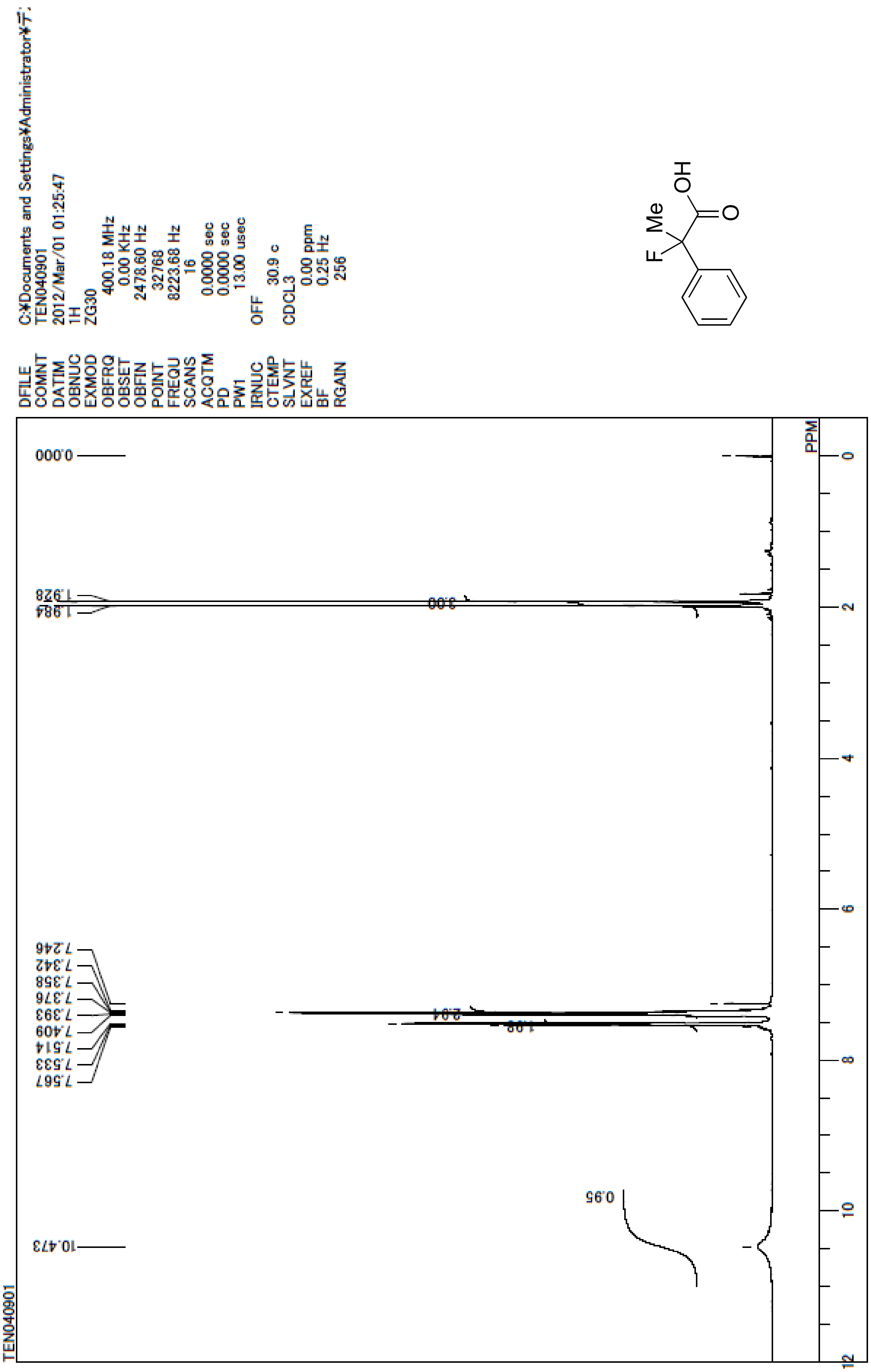
C:¥Documents and Settings¥Administrator¥デ:
DFILE
COMNT TEN040901
DATIM 2012/Mar/01 01:25:47
OBNUC 1H
EXMOD ZG30
OBFRQ 400.18 MHz
OBSET 0.00 KHz
OBFIN 2478.60 Hz
POINT 32768
FREQU 8223.68 Hz
SCANS 16
ACQTM 0.0000 sec
PD 0.0000 sec
PW1 13.00 usec
IRNUC OFF
CTEMP 30.9 c
SLVNT CDCL3
EXREF 0.00 ppm
BF 0.25 Hz
RGAIN 256
F
Me
OH
O
0.000
1.928
1.984
7.246
7.342
7.358
7.376
7.393
7.409
7.514
7.533
7.567
10.473
3.00
2.04
1.08
0.95
TEN040901
PPM
0
2
4
6
8
10
12

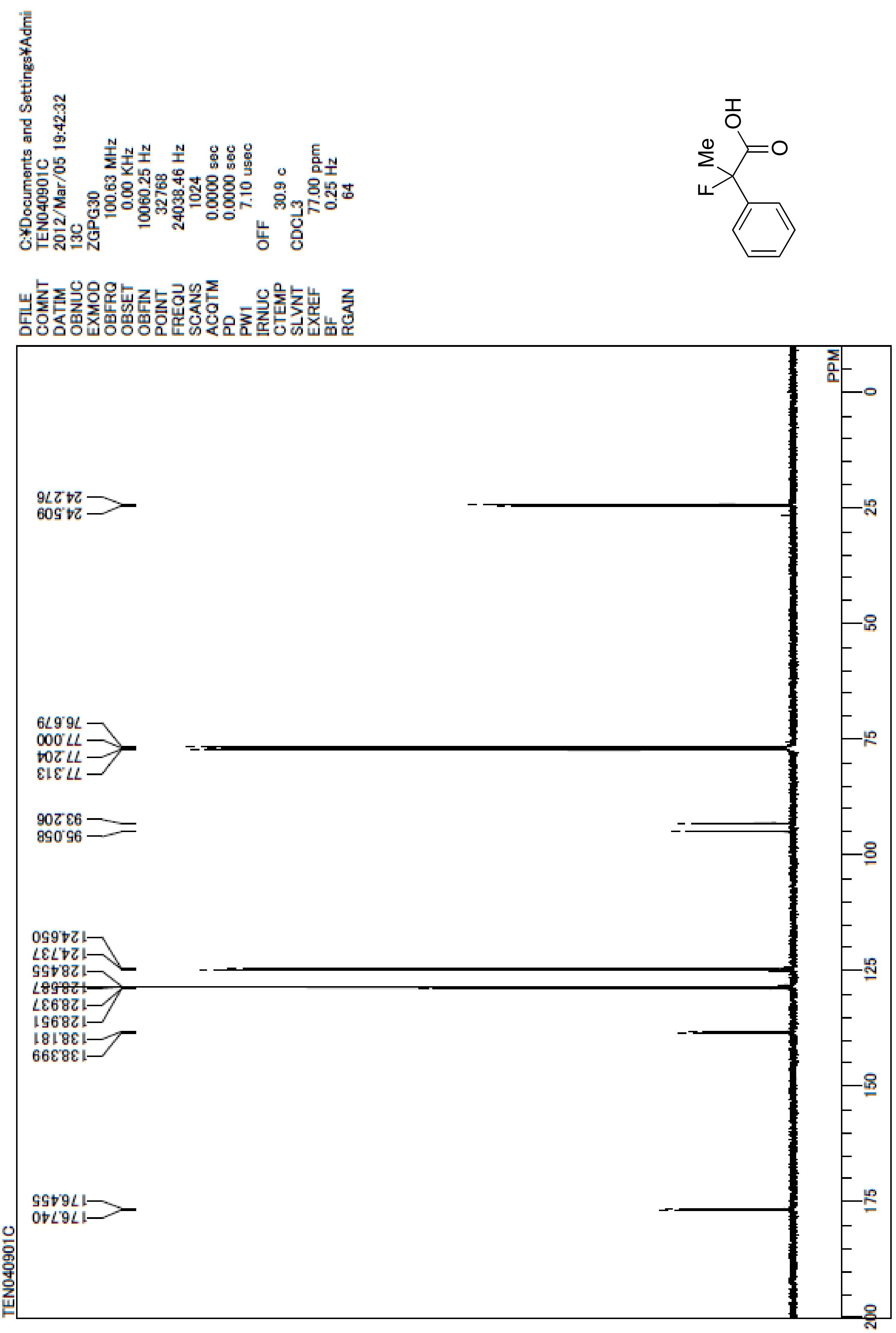
DFILE C:¥Documents and Settings¥Admi
COMNT TEN040901C
DATIM 2012/Mar/05 19:42:32
OBNUC 13C
EXMOD ZGPG30
OBFRQ 100.63 MHz
OBSET 0.00 KHz
OBFIN 10060.25 Hz
POINT 32768
FREQU 24038.46 Hz
SCANS 1024
ACQTM 0.0000 sec
PD 0.0000 sec
PW1 7.10 usec
IRNUC OFF
CTEMP 30.9 c
SLVNT CDCL3
EXREF 77.00 ppm
BF 0.25 Hz
RGAIN 64
F
Me
OH
O
24.276
24.509
76.679
77.000
77.204
77.313
93.206
95.058
124.650
124.737
128.455
128.587
128.937
128.951
138.181
138.399
176.455
176.740
TEN040901C
PPM
0
25
50
75
100
125
150
175
200

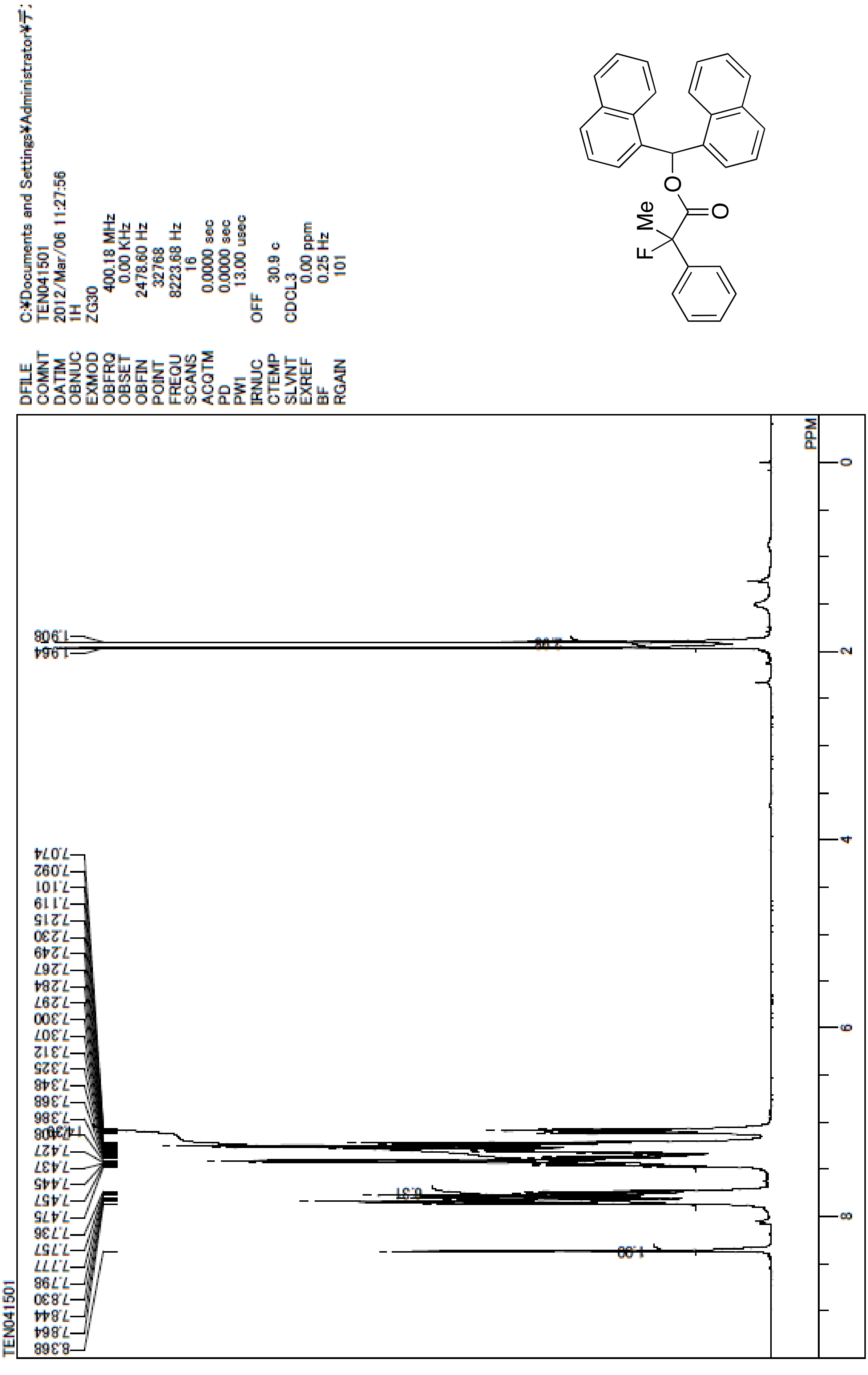
DFILE C:¥Documents and Settings¥Administrator¥デ;
COMNT TEN041501
DATIM 2012/Mar/06 11:27:56
OBNUC 1H
EXMOD ZG30
OBFRQ 400.18 MHz
OBSET 0.00 KHz
OBFIN 2478.60 Hz
POINT 32768
FREQU 8223.68 Hz
SCANS 16
ACQTM 0.0000 sec
PD 0.0000 sec
PW1 13.00 usec
IRNUC OFF
CTEMP 30.9 c
SLVNT CDCL3
EXREF 0.00 ppm
BF 0.25 Hz
RGAIN 101
F
Me
O
O
TEN041501
PPM

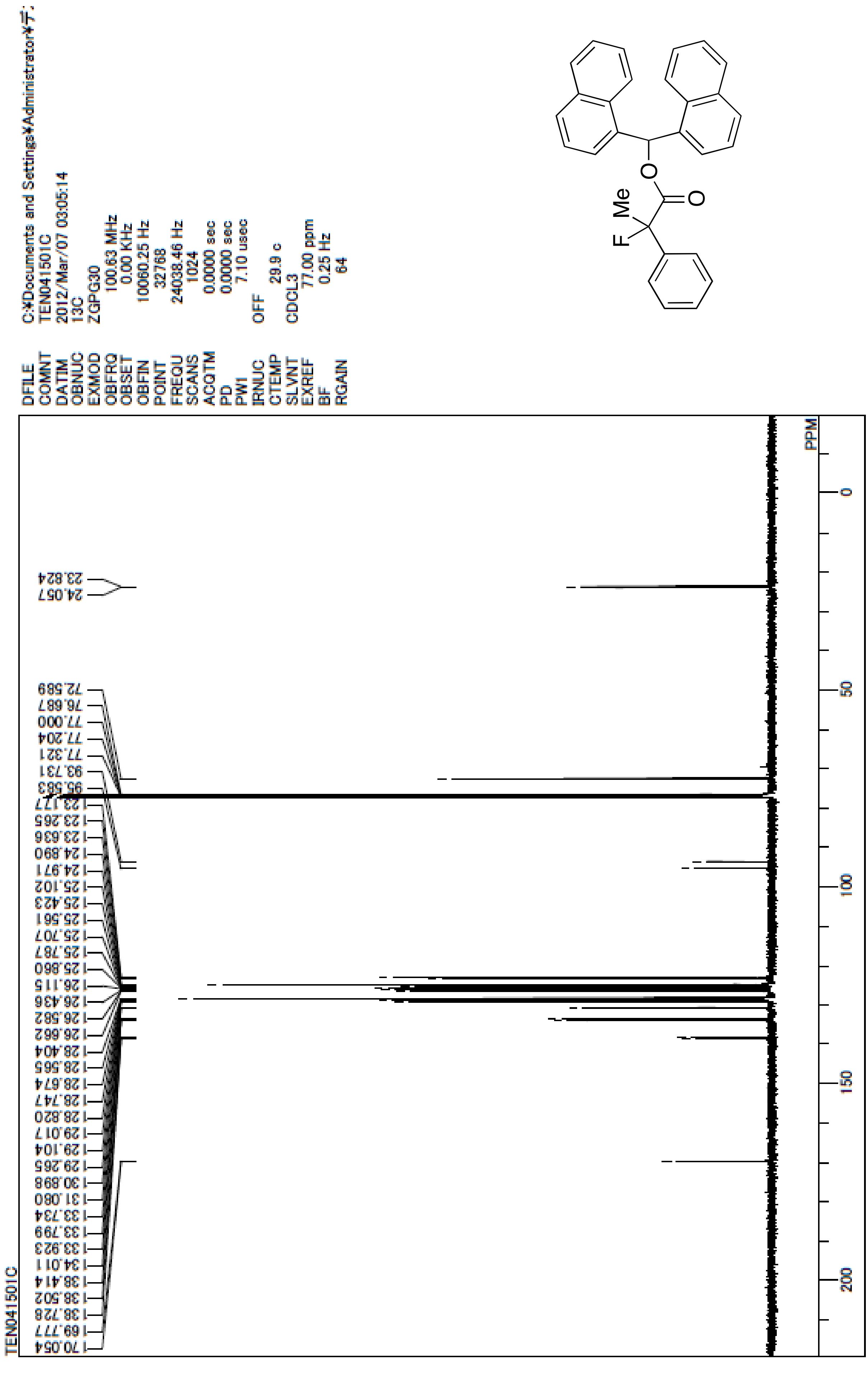
DFILE C:¥Documents and Settings¥Administrator¥デ
COMNT TEN041501C
DATIM 2012/Mar/07 03:05:14
OBNUC 13C
EXMOD ZGPG30
OBFRQ 100.63 MHz
OBSET 0.00 KHz
OBFIN 10060.25 Hz
POINT 32768
FREQU 24038.46 Hz
SCANS 1024
ACQTM 0.0000 sec
PD 0.0000 sec
PW1 7.10 usec
IRNUC OFF
CTEMP 29.9 c
SLVNT CDCL3
EXREF 77.00 ppm
BF 0.25 Hz
RGAIN 64
TEN041501C
23.824
24.057
72.589
76.687
77.000
77.204
77.321
93.731
95.583
123.177
123.265
123.636
124.890
124.971
125.102
125.423
125.561
125.707
125.787
125.860
126.115
126.436
126.582
126.662
128.404
128.565
128.674
128.747
128.820
129.017
129.104
129.265
130.898
131.080
133.734
133.799
133.923
134.011
138.414
138.502
138.728
169.777
170.054
PPM
0
50
100
150
200
F
Me
O
O

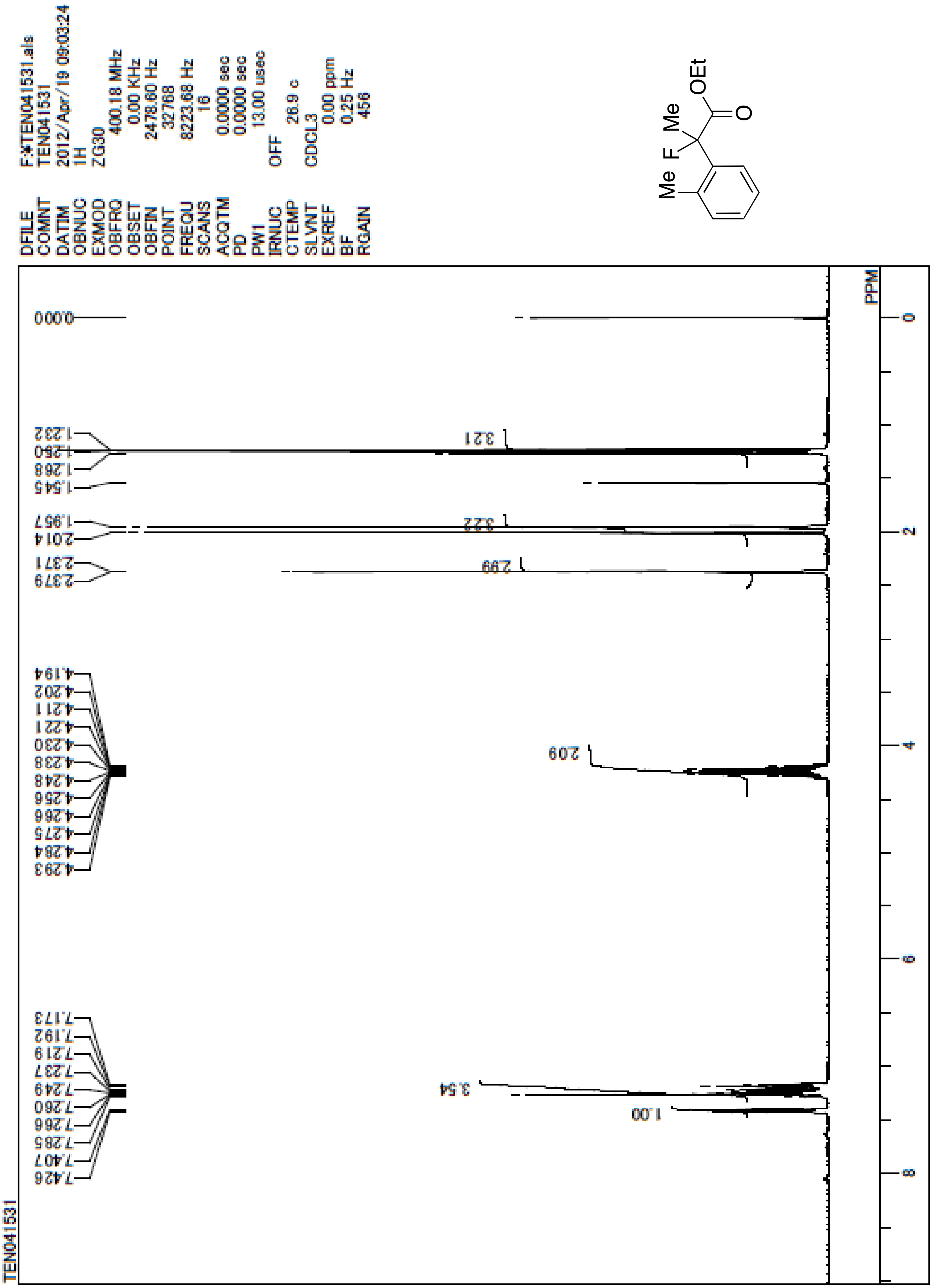

DFILE F:¥TEN041531.als
COMNT TEN041531
DATIM 2012/Apr/19 09:03:24
OBNUC 1H
EXMOD ZG30
OBFRQ 400.18 MHz
OBSET 0.00 KHz
OBFIN 2478.60 Hz
POINT 32768
FREQU 8223.68 Hz
SCANS 16
ACQTM 0.0000 sec
PD 0.0000 sec
PW1 13.00 usec
IRNUC OFF
CTEMP 26.9 c
SLVNT CDCL3
EXREF 0.00 ppm
BF 0.25 Hz
RGAIN 456
Me F Me OEt O
TEN041531
PPM

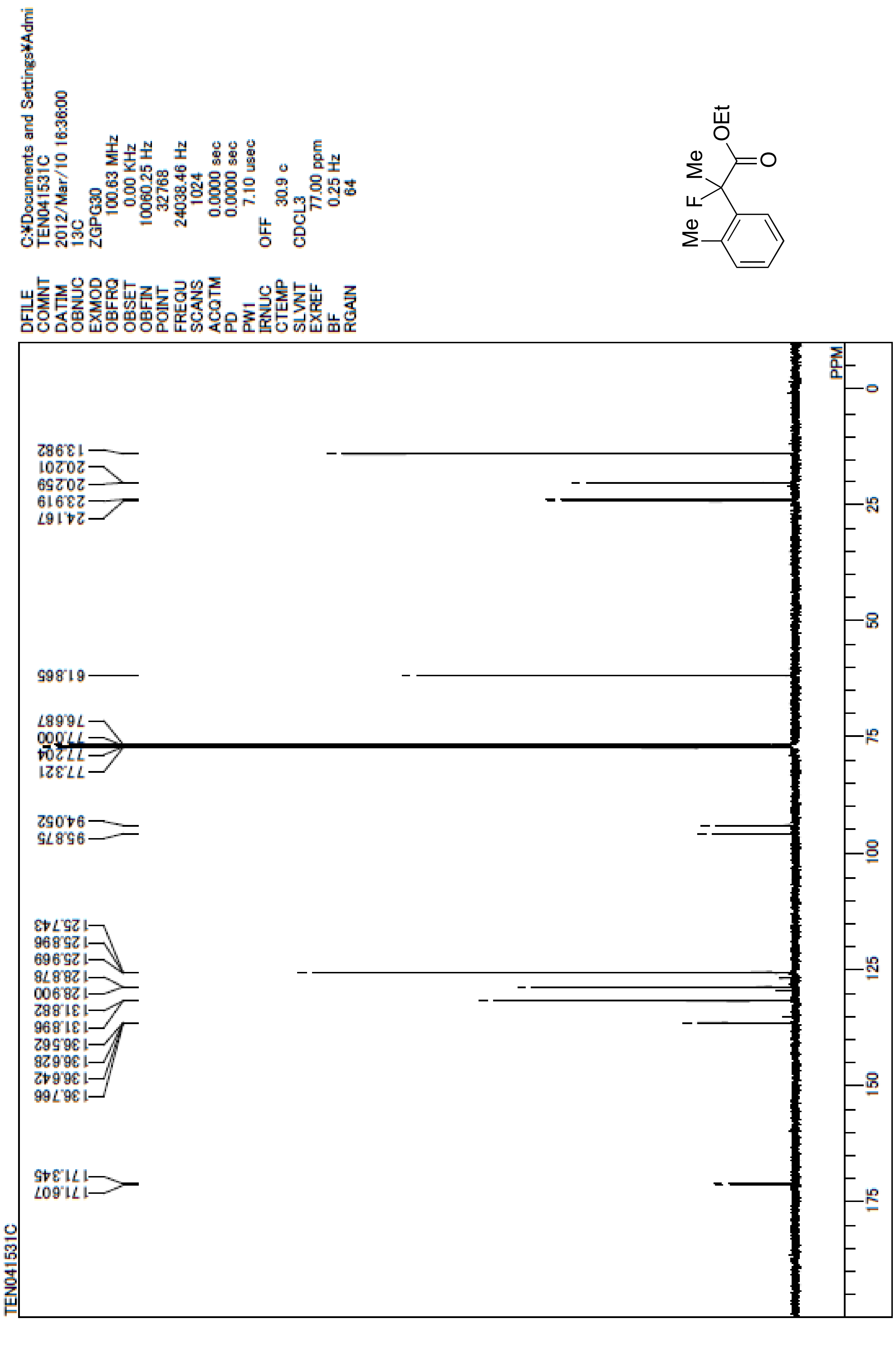
DFILE C:¥Documents and Settings¥Admi
COMNT TEN041531C
DATIM 2012/Mar/10 16:36:00
OBNUC 13C
EXMOD ZGPG30
OBFRQ 100.63 MHz
OBSET 0.00 KHz
OBFIN 10060.25 Hz
POINT 32768
FREQU 24038.46 Hz
SCANS 1024
ACQTM 0.0000 sec
PD 0.0000 sec
PW1 7.10 usec
IRNUC OFF
CTEMP 30.9 c
SLVNT CDCL3
EXREF 77.00 ppm
BF 0.25 Hz
RGAIN 64
Me
F
Me
OEt
O
TEN041531C
13.982
20.201
20.259
23.919
24.167
61.865
76.687
77.000
77.204
77.321
94.052
95.875
125.743
125.896
125.969
128.878
128.900
131.882
131.896
136.562
136.628
136.642
136.766
171.345
171.607
PPM
0
25
50
75
100
125
150
175

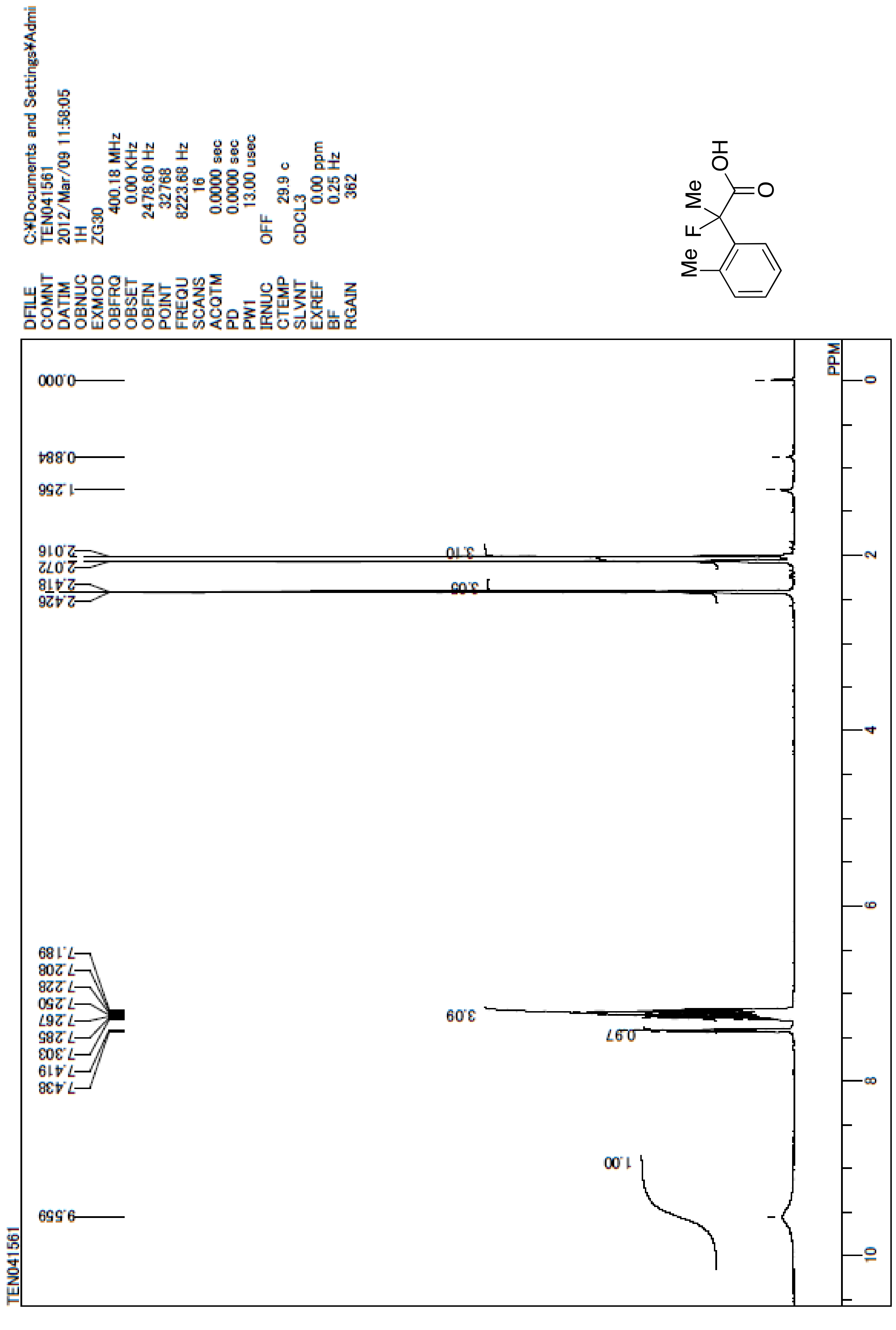
DFILE C:¥Documents and Settings¥Admi
COMNT TEN041561
DATIM 2012/Mar/09 11:58:05
OBNUC 1H
EXMOD ZG30
OBFRQ 400.18 MHz
OBSET 0.00 KHz
OBFIN 2478.60 Hz
POINT 32768
FREQU 8223.68 Hz
SCANS 16
ACQTM 0.0000 sec
PD 0.0000 sec
PW1 13.00 usec
IRNUC OFF
CTEMP 29.9 c
SLVNT CDCL3
EXREF 0.00 ppm
BF 0.25 Hz
RGAIN 362
TEN041561
PPM
Me
F
Me
OH
O

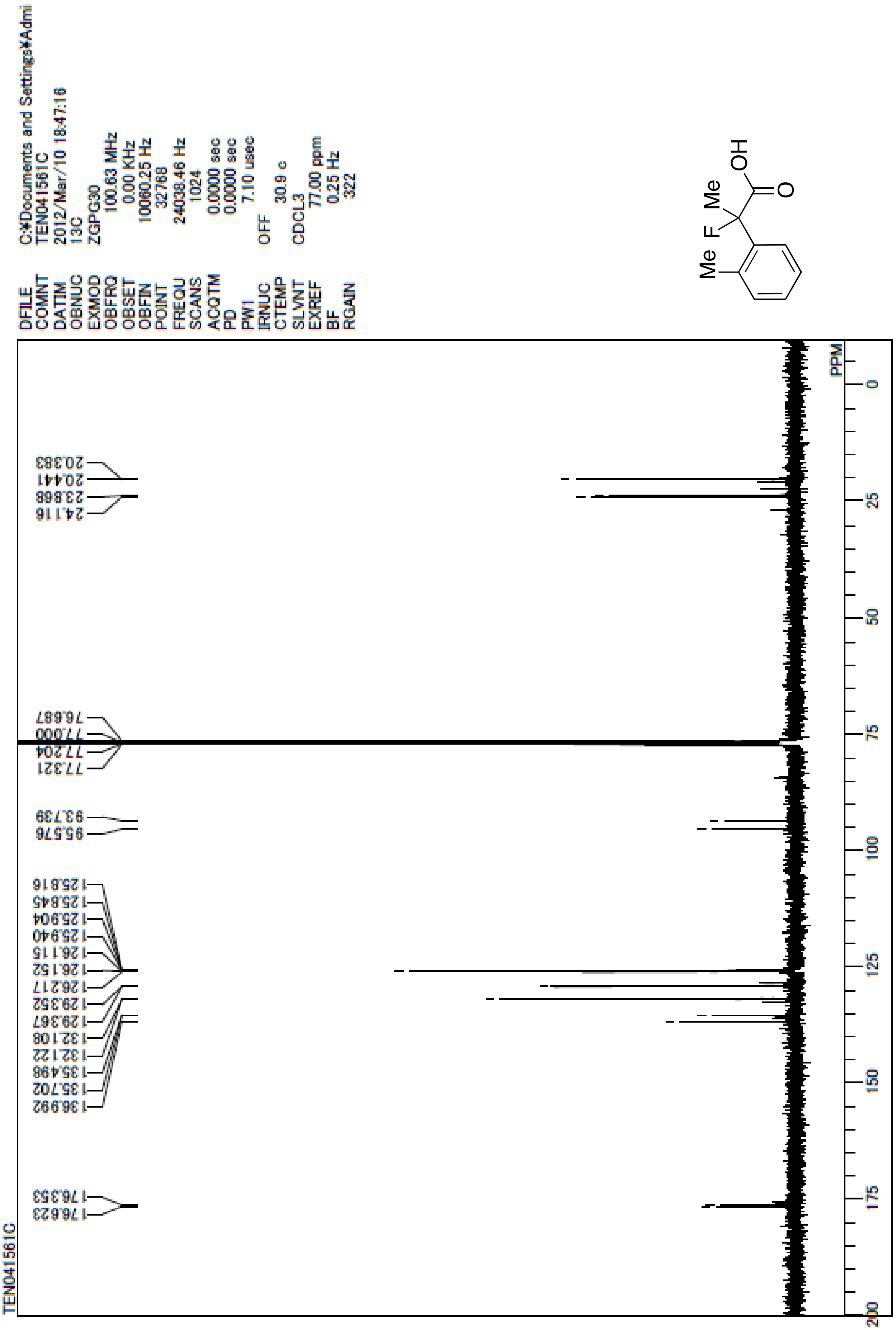
DFILE C:¥Documents and Settings¥Admi
COMNT TEN041561C
DATIM 2012/Mar/10 18:47:16
OBNUC 13C
EXMOD ZGPG30
OBFRQ 100.63 MHz
OBSET 0.00 KHz
OBFIN 10060.25 Hz
POINT 32768
FREQU 24038.46 Hz
SCANS 1024
ACQTM 0.0000 sec
PD 0.0000 sec
PW1 7.10 usec
IRNUC OFF
CTEMP 30.9 c
SLVNT CDCL3
EXREF 77.00 ppm
BF 0.25 Hz
RGAIN 322
Me
F
Me
OH
O
20.383
20.441
23.868
24.116
76.687
77.000
77.204
77.321
93.739
95.576
125.816
125.845
125.904
125.940
126.115
126.152
126.217
129.352
129.367
132.108
132.122
135.498
135.702
136.992
176.353
176.623
TEN041561C
PPM
0
25
50
75
100
125
150
175
200

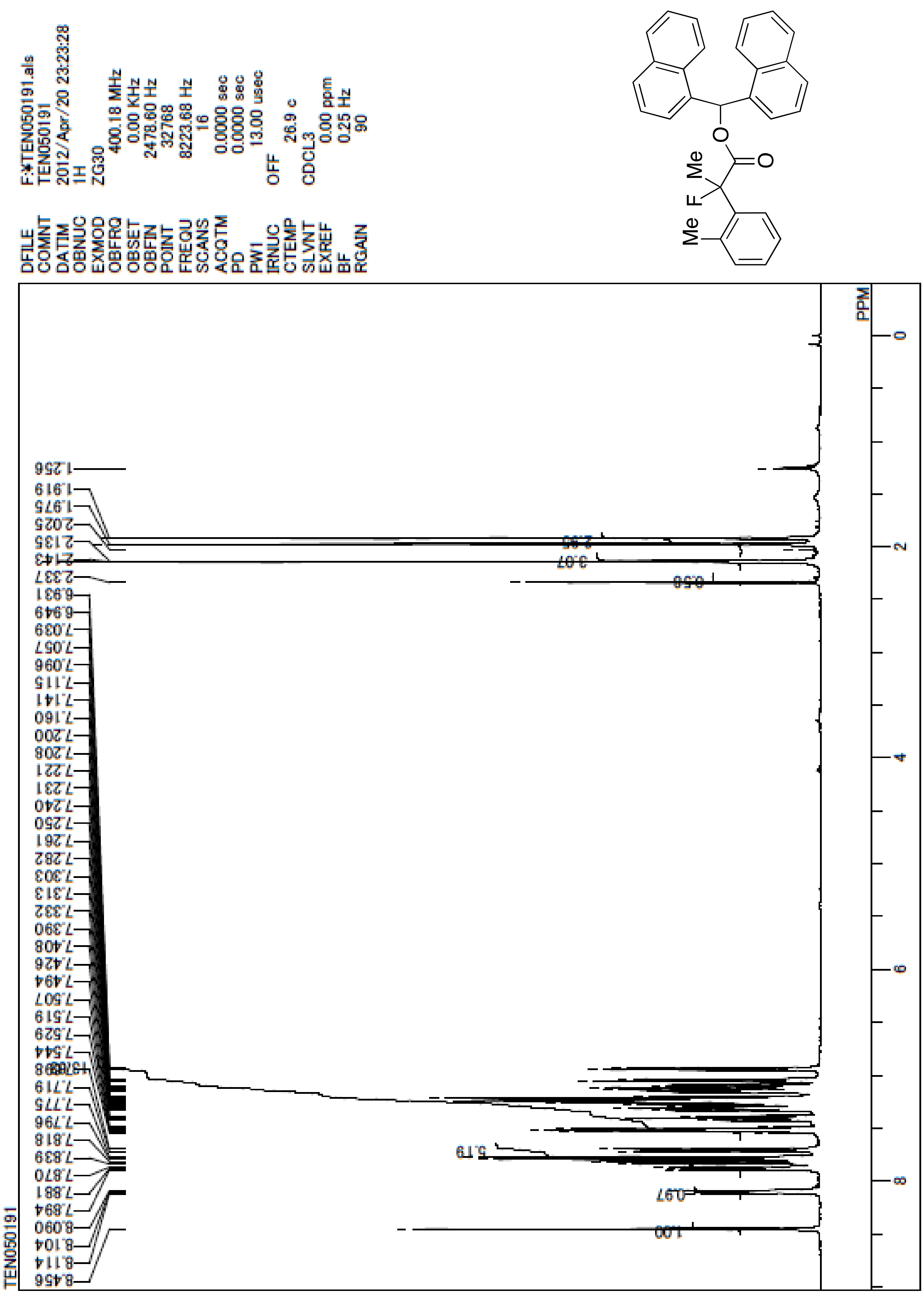
DFILE F:¥TEN050191.als
COMNT TEN050191
DATIM 2012/Apr/20 23:23:28
OBNUC 1H
EXMOD ZG30
OBFRQ 400.18 MHz
OBSET 0.00 KHz
OBFIN 2478.60 Hz
POINT 32768
FREQU 8223.68 Hz
SCANS 16
ACQTM 0.0000 sec
PD 0.0000 sec
PW1 13.00 usec
IRNUC OFF
CTEMP 26.9 c
SLVNT CDCL3
EXREF 0.00 ppm
BF 0.25 Hz
RGAIN 90
Me
F
Me
O
O
TEN050191
PPM

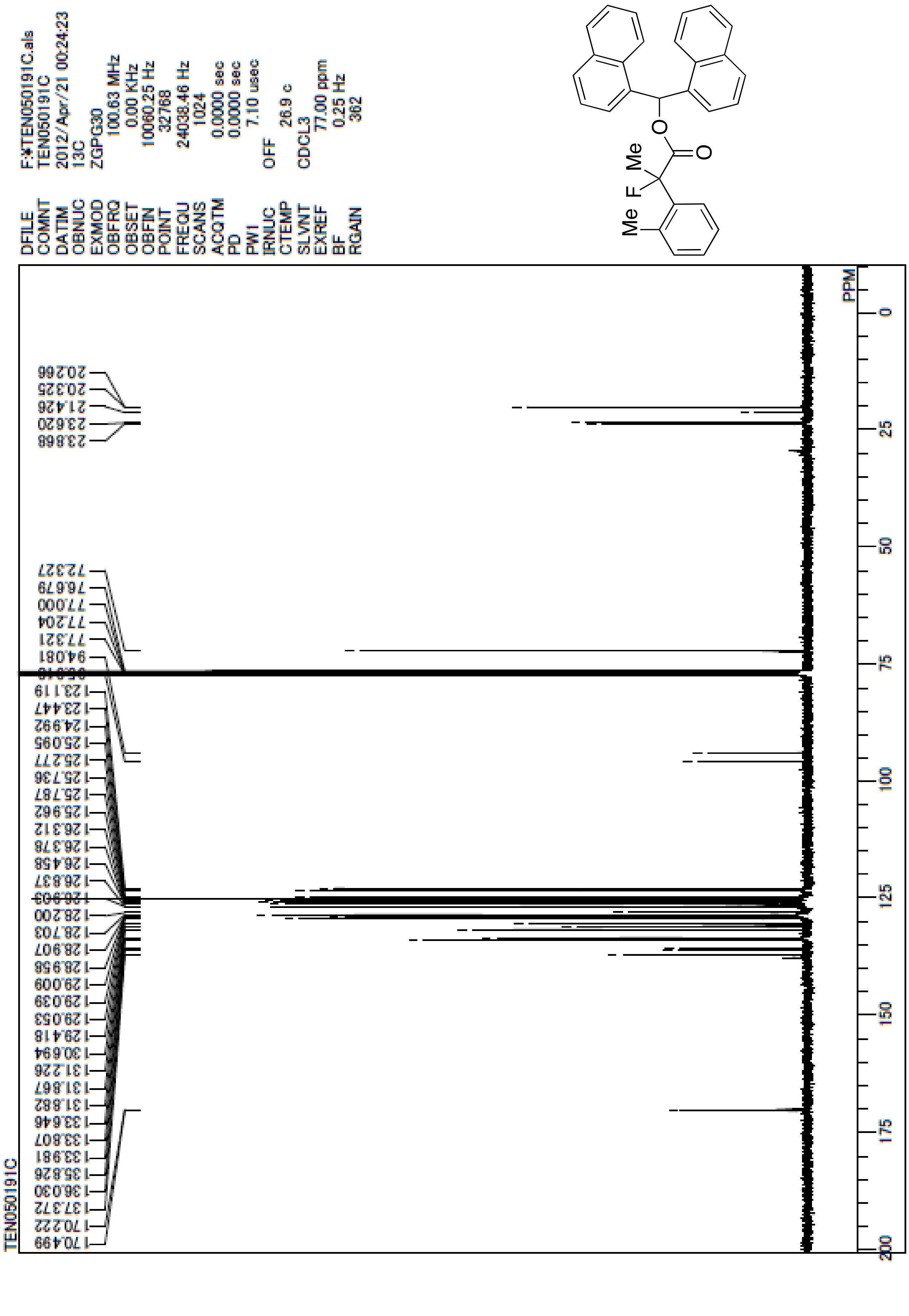
TEN050191C
DFILE F:¥TEN050191C.als
COMNT TEN050191C
DATIM 2012/Apr/21 00:24:23
OBNUC 13C
EXMOD ZGPG30
OBFRQ 100.63 MHz
OBSET 0.00 KHz
OBFIN 10060.25 Hz
POINT 32768
FREQU 24038.46 Hz
SCANS 1024
ACQTM 0.0000 sec
PD 0.0000 sec
PW1 7.10 usec
IRNUC OFF
CTEMP 26.9 c
SLVNT CDCL3
EXREF 77.00 ppm
BF 0.25 Hz
RGAIN 362
Me
F
Me
O
O
20.266
20.325
21.426
23.620
23.666
72.327
76.679
77.000
77.204
77.321
94.081
123.119
123.447
124.992
125.095
125.277
125.736
125.787
125.962
126.312
126.378
126.458
126.837
126.903
128.200
128.703
128.907
128.958
129.009
129.039
129.053
129.418
130.694
131.226
131.867
131.882
133.646
133.807
133.981
135.826
136.030
137.372
170.222
170.499
PPM
0
25
50
75
100
125
150
175
200

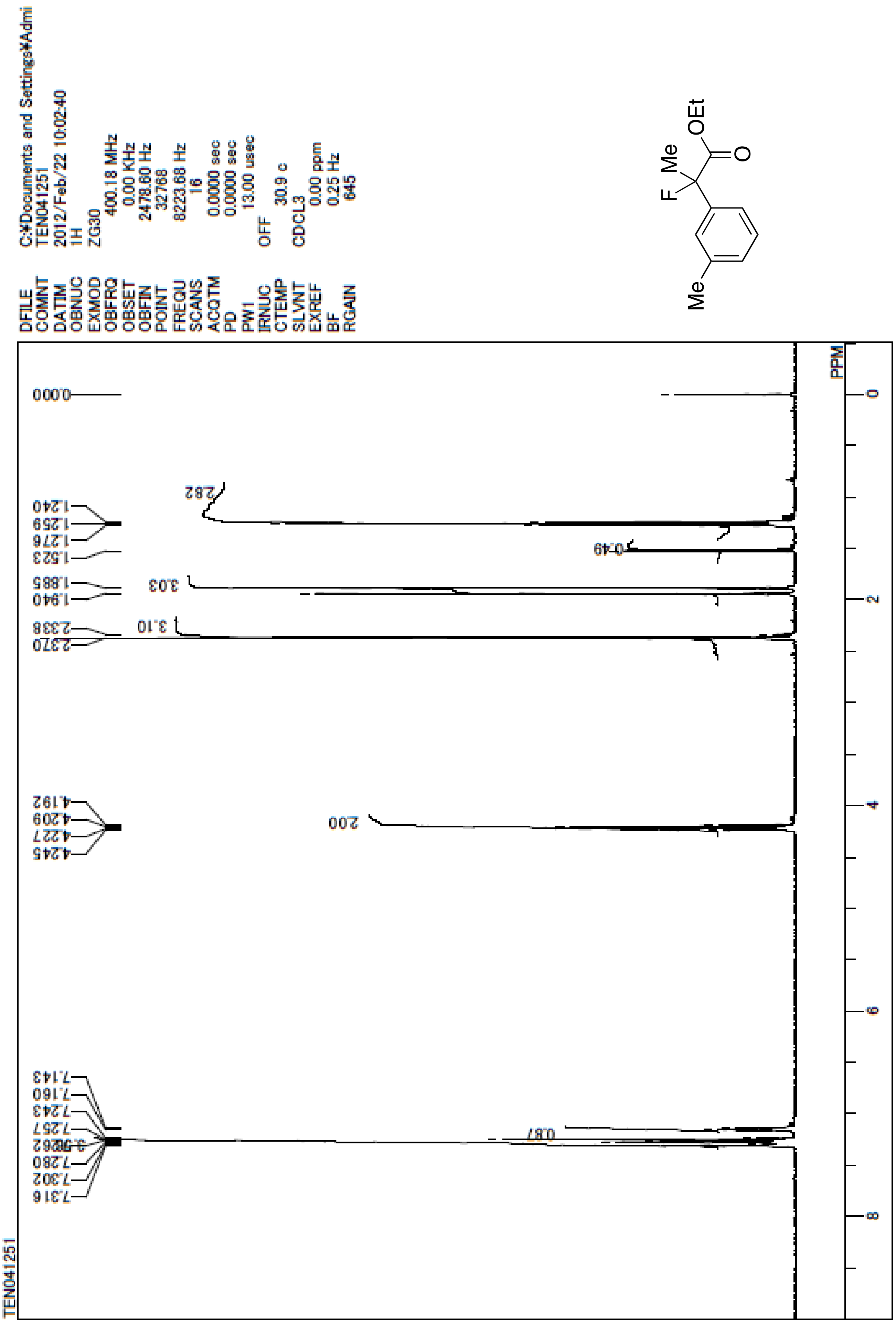
DFILE C:¥Documents and Settings¥Admi
COMNT TEN041251
DATIM 2012/Feb/22 10:02:40
OBNUC 1H
EXMOD ZG30
OBFRQ 400.18 MHz
OBSET 0.00 KHz
OBFIN 2478.60 Hz
POINT 32768
FREQU 8223.68 Hz
SCANS 16
ACQTM 0.0000 sec
PD 0.0000 sec
PW1 13.00 usec
IRNUC OFF
CTEMP 30.9 c
SLVNT CDCL3
EXREF 0.00 ppm
BF 0.25 Hz
RGAIN 645
TEN041251
F
Me
OEt
O
Me
PPM

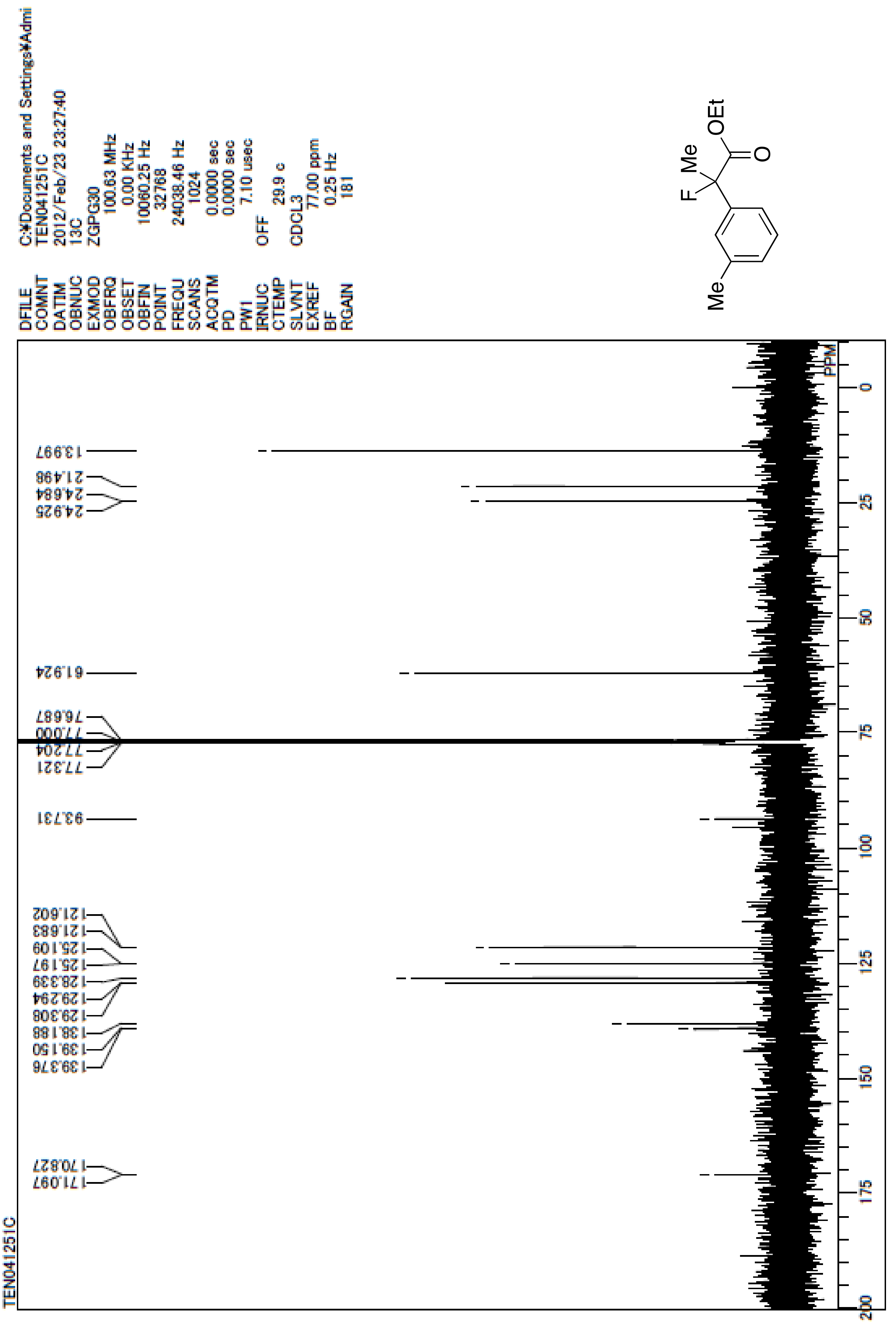
DFILE C:¥Documents and Settings¥Admi
COMNT TEN041251C
DATIM 2012/Feb/23 23:27:40
OBNUC 13C
EXMOD ZGPG30
OBFRQ 100.63 MHz
OBSET 0.00 KHz
OBFIN 10060.25 Hz
POINT 32768
FREQU 24038.46 Hz
SCANS 1024
ACQTM 0.0000 sec
PD 0.0000 sec
PW1 7.10 usec
IRNUC OFF
CTEMP 29.9 c
SLVNT CDCL3
EXREF 77.00 ppm
BF 0.25 Hz
RGAIN 181
Me
F
Me
O
OEt
TEN041251C
13.997
21.498
24.684
24.925
61.924
76.687
77.000
77.204
77.321
93.731
121.602
121.683
125.109
125.197
128.339
129.294
129.308
138.188
139.150
139.376
170.827
171.097
PPM
0
25
50
75
100
125
150
175
200

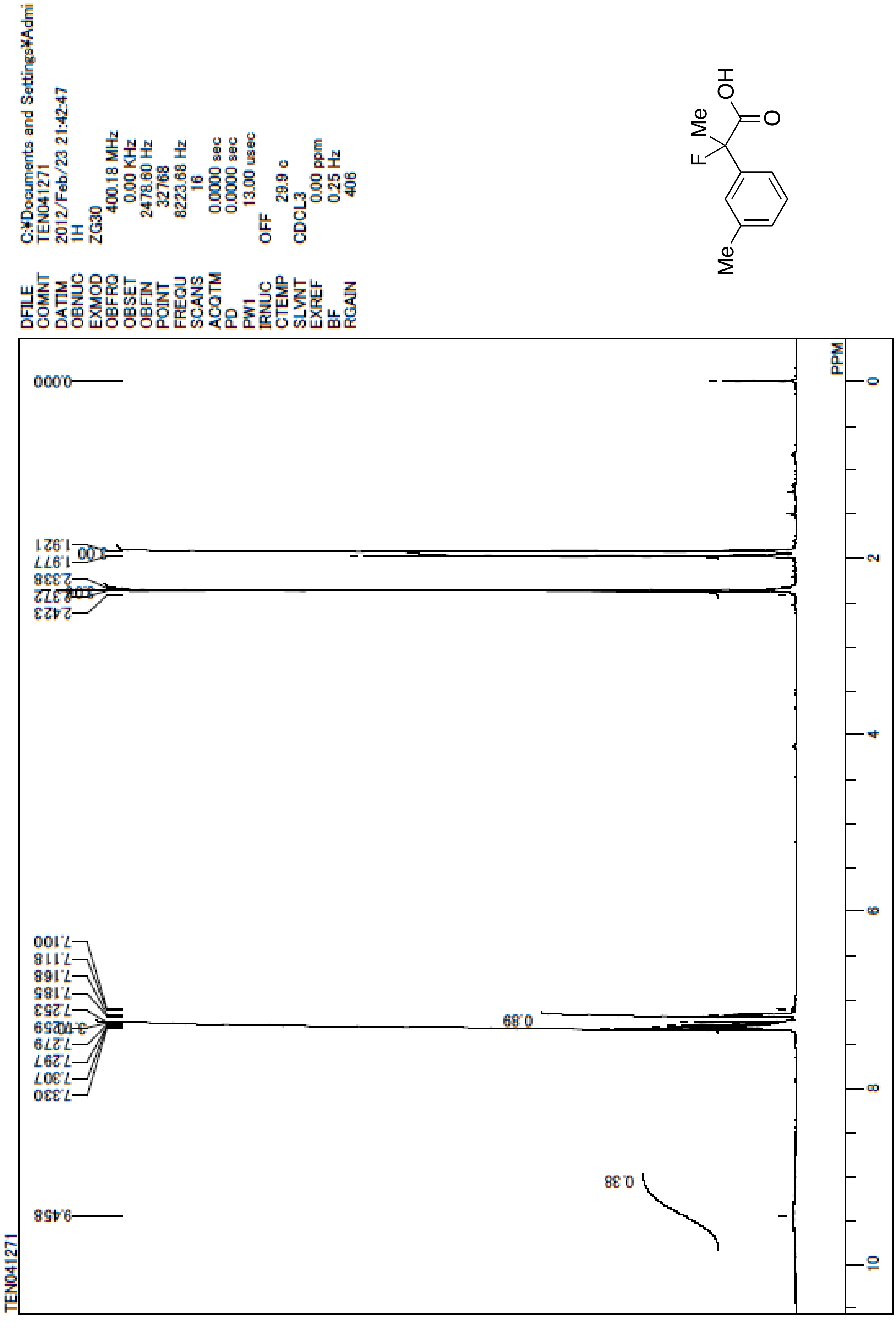
DFILE C:¥Documents and Settings¥Admi
COMNT TEN041271
DATIM 2012/Feb/23 21:42:47
OBNUC 1H
EXMOD ZG30
OBFRQ 400.18 MHz
OBSET 0.00 KHz
OBFIN 2478.60 Hz
POINT 32768
FREQU 8223.68 Hz
SCANS 16
ACQTM 0.0000 sec
PD 0.0000 sec
PW1 13.00 usec
IRNUC OFF
CTEMP 29.9 c
SLVNT CDCL3
EXREF 0.00 ppm
BF 0.25 Hz
RGAIN 406
F
Me
OH
O
Me
TEN041271
0.000
1.921
1.977
2.338
2.372
2.423
7.100
7.118
7.168
7.185
7.253
7.259
7.279
7.297
7.307
7.330
9.458
0.89
0.38
PPM
0
2
4
6
8
10

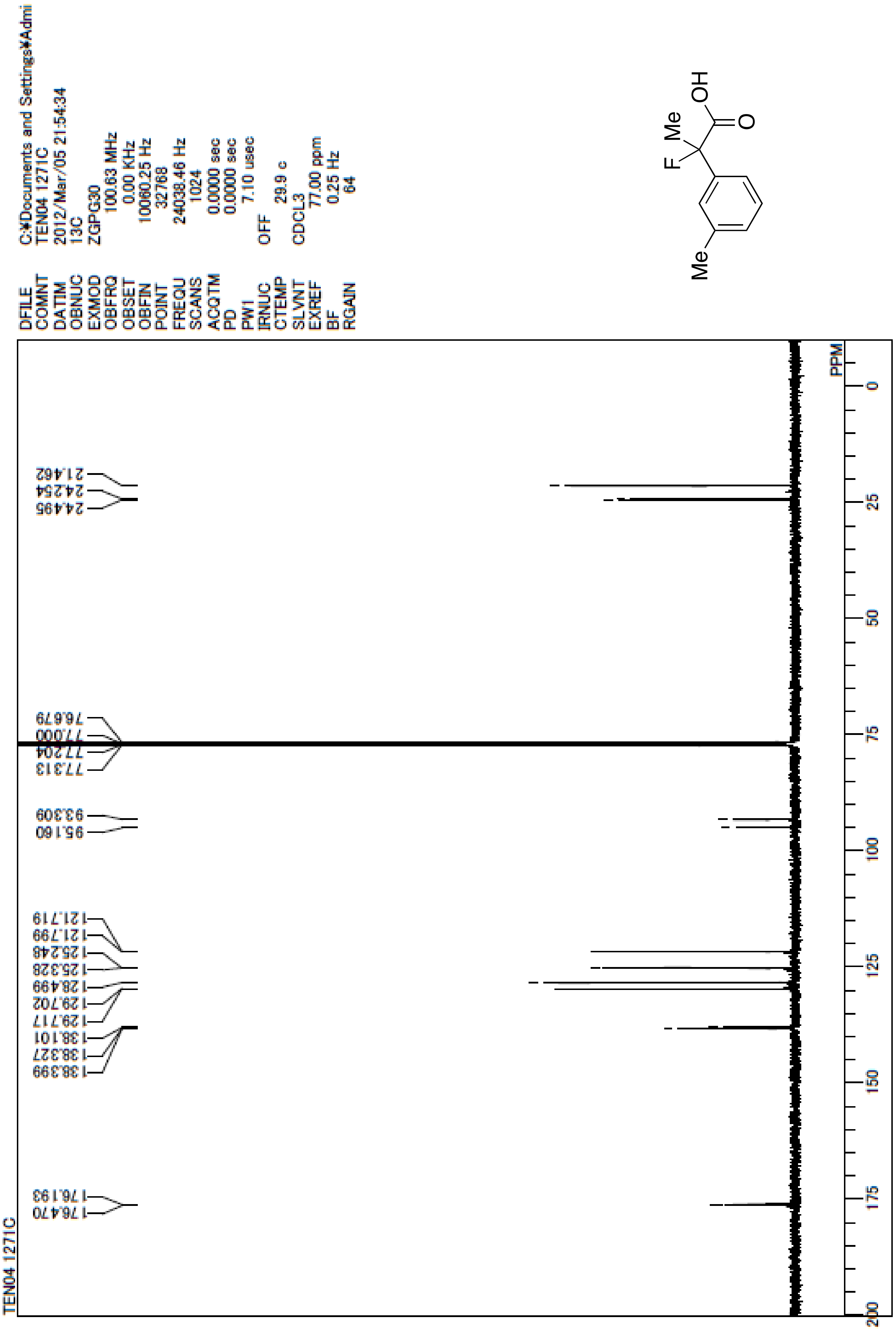
DFILE C:¥Documents and Settings¥Admi
COMNT TEN04 1271C
DATIM 2012/Mar/05 21:54:34
OBNUC 13C
EXMOD ZGPG30
OBFRQ 100.63 MHz
OBSET 0.00 KHz
OBFIN 10060.25 Hz
POINT 32768
FREQU 24038.46 Hz
SCANS 1024
ACQTM 0.0000 sec
PD 0.0000 sec
PW1 7.10 usec
IRNUC OFF
CTEMP 29.9 c
SLVNT CDCL3
EXREF 77.00 ppm
BF 0.25 Hz
RGAIN 64
TEN04 1271C
F
Me
OH
O
Me
21.462
24.254
24.495
76.679
77.000
77.204
77.313
93.309
95.160
121.719
121.799
125.248
125.328
128.499
129.702
129.717
138.101
138.327
138.399
176.193
176.470
PPM
0
25
50
75
100
125
150
175
200

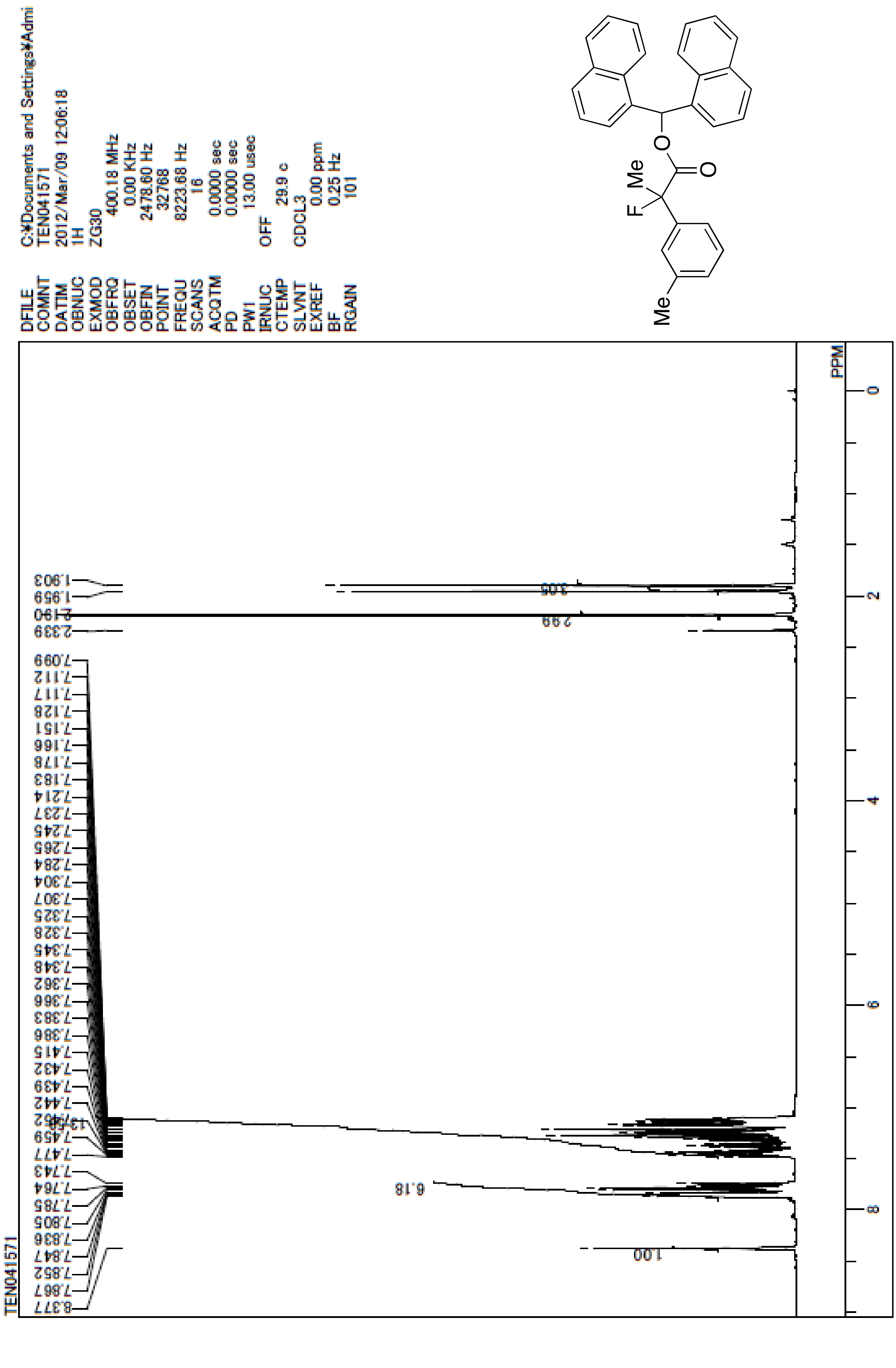

DFILE C:¥Documents and Settings¥Admi
COMNT TEN041571
DATIM 2012/Mar/09 12:06:18
OBNUC 1H
EXMOD ZG30
OBFRQ 400.18 MHz
OBSET 0.00 KHz
OBFIN 2478.60 Hz
POINT 32768
FREQU 8223.68 Hz
SCANS 16
ACQTM 0.0000 sec
PD 0.0000 sec
PW1 13.00 usec
IRNUC OFF
CTEMP 29.9 c
SLVNT CDCL3
EXREF 0.00 ppm
BF 0.25 Hz
RGAIN 101
TEN041571
PPM

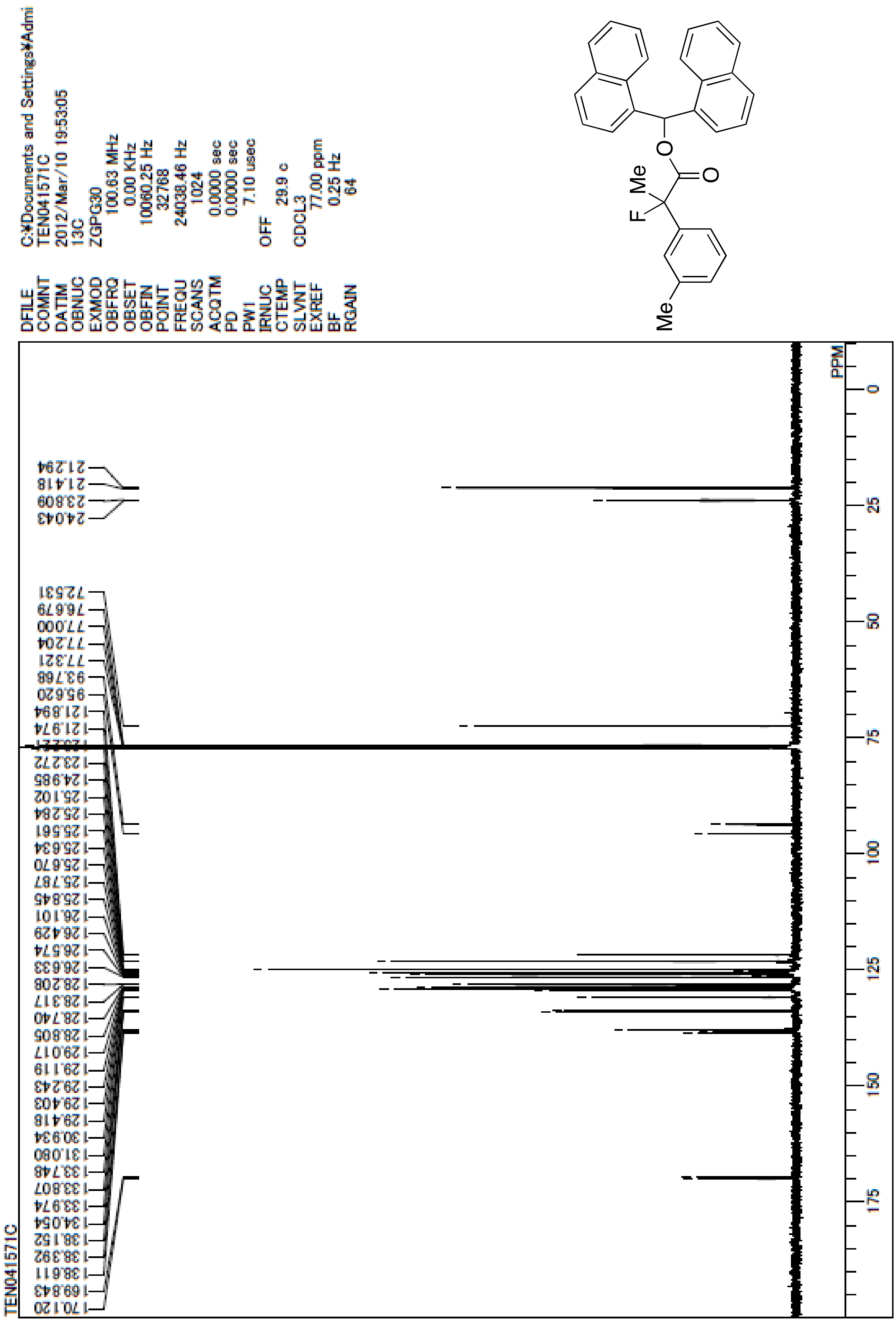
DFILE C:¥Documents and Settings¥Admi
COMNT TEN041571C
DATIM 2012/Mar/10 19:53:05
OBNUC 13C
EXMOD ZGPG30
OBFRQ 100.63 MHz
OBSET 0.00 KHz
OBFIN 10060.25 Hz
POINT 32768
FREQU 24038.46 Hz
SCANS 1024
ACQTM 0.0000 sec
PD 0.0000 sec
PW1 7.10 usec
IRNUC OFF
CTEMP 29.9 c
SLVNT CDCL3
EXREF 77.00 ppm
BF 0.25 Hz
RGAIN 64
TEN041571C
21.294
21.418
23.809
24.043
72.531
76.679
77.000
77.204
77.321
93.768
95.620
121.894
121.974
123.272
124.985
125.102
125.284
125.561
125.634
125.670
125.787
125.845
126.101
126.429
126.574
126.633
128.208
128.317
128.740
128.805
129.017
129.119
129.243
129.403
129.418
130.934
131.080
133.748
133.807
133.974
134.054
138.152
138.392
138.611
169.843
170.120
PPM
0
25
50
75
100
125
150
175
F
Me
O
O
Me

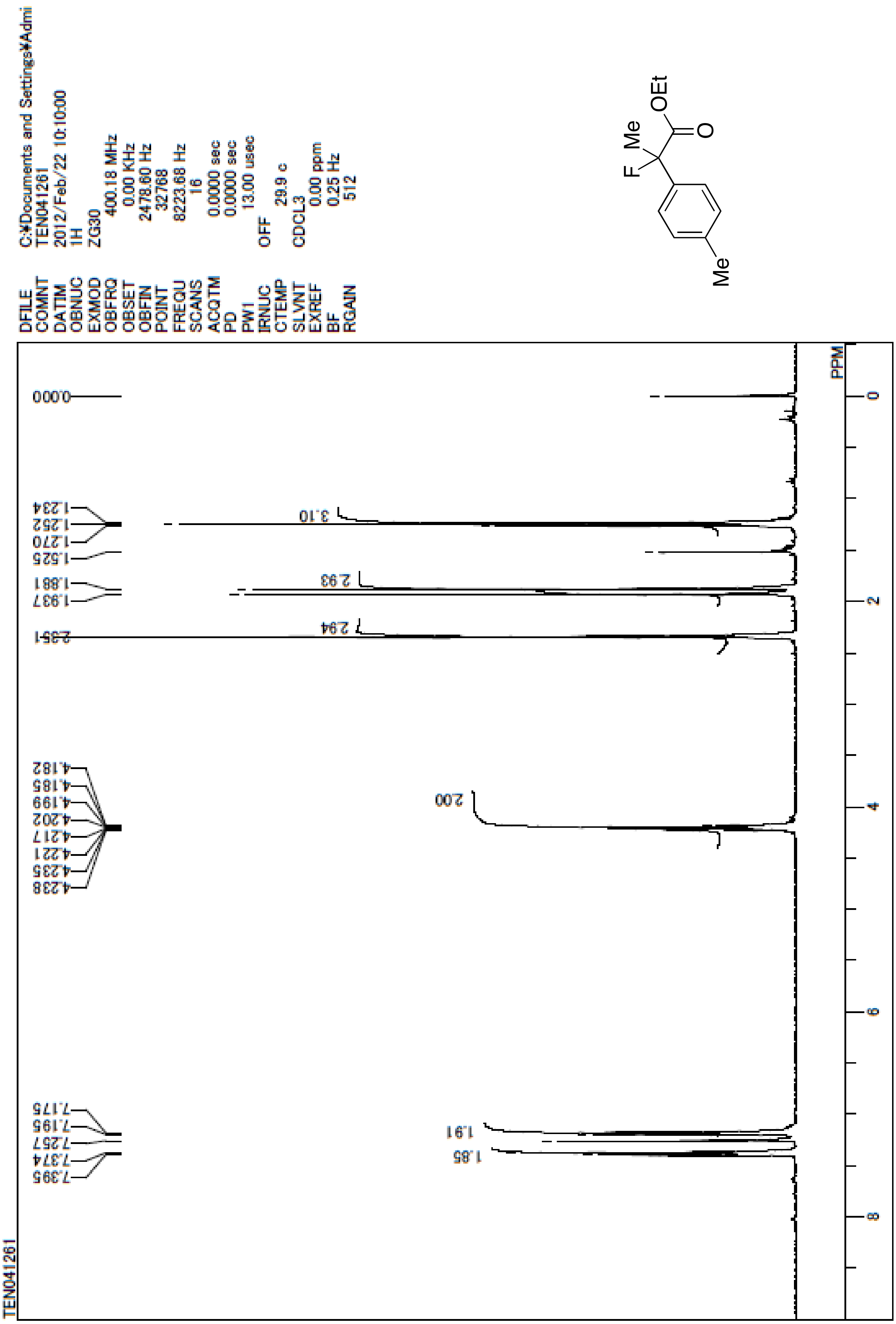
DFILE C:¥Documents and Settings¥Admi
COMNT TEN041261
DATIM 2012/Feb/22 10:10:00
OBNUC 1H
EXMOD ZG30
OBFRQ 400.18 MHz
OBSET 0.00 KHz
OBFIN 2478.60 Hz
POINT 32768
FREQU 8223.68 Hz
SCANS 16
ACQTM 0.0000 sec
PD 0.0000 sec
PW1 13.00 usec
IRNUC OFF
CTEMP 29.9 c
SLVNT CDCL3
EXREF 0.00 ppm
BF 0.25 Hz
RGAIN 512
F
Me
OEt
O
Me
0.000
1.234
1.252
1.270
1.525
1.881
1.937
2.351
4.182
4.185
4.199
4.202
4.217
4.221
4.235
4.238
7.175
7.195
7.257
7.374
7.395
3.10
2.93
2.94
2.00
1.91
1.85
PPM
0
2
4
6
8
TEN041261

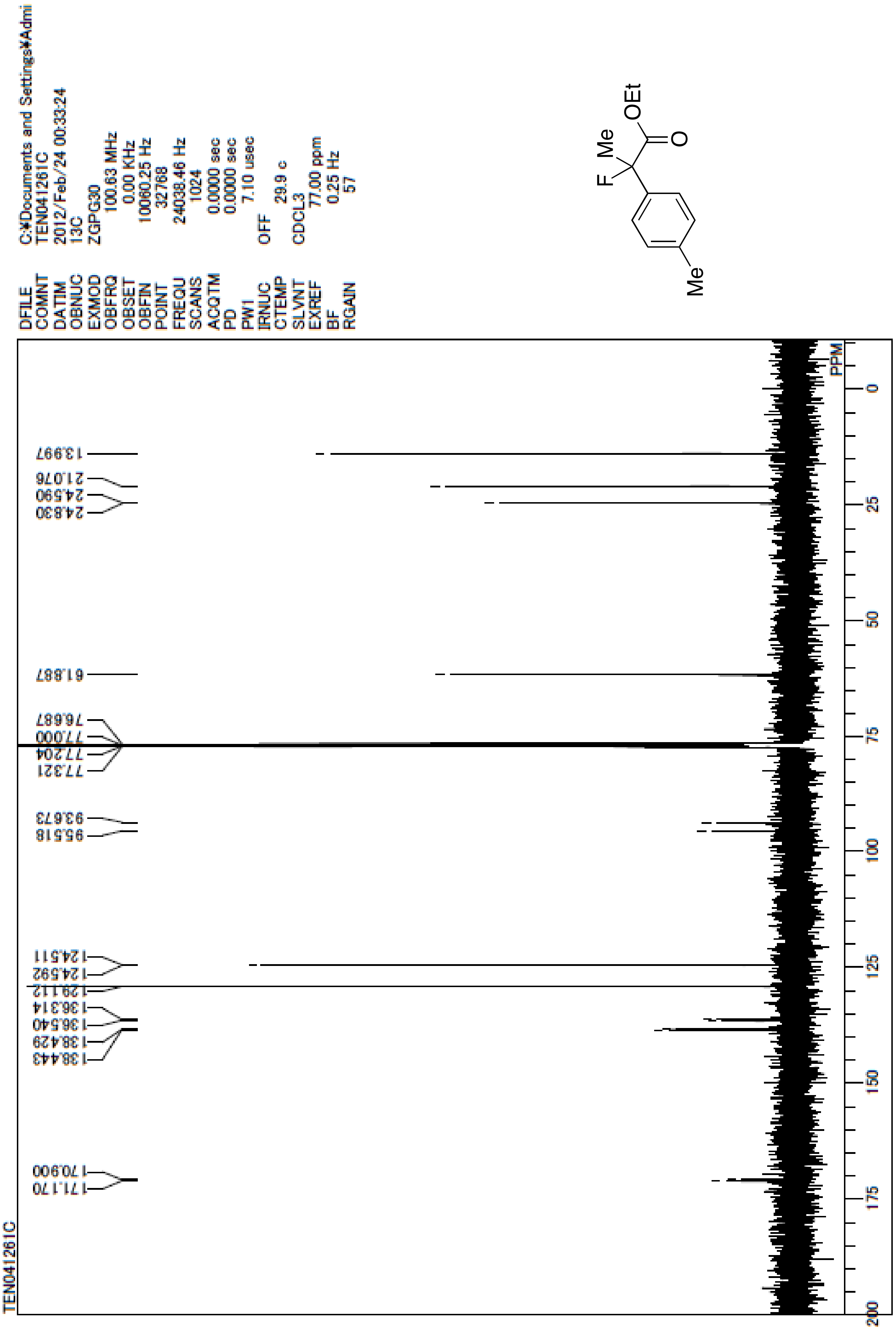
DFILE C:¥Documents and Settings¥Admi
COMNT TEN041261C
DATIM 2012/Feb/24 00:33:24
OBNUC 13C
EXMOD ZGPG30
OBFRQ 100.63 MHz
OBSET 0.00 KHz
OBFIN 10060.25 Hz
POINT 32768
FREQU 24038.46 Hz
SCANS 1024
ACQTM 0.0000 sec
PD 0.0000 sec
PW1 7.10 usec
IRNUC OFF
CTEMP 29.9 c
SLVNT CDCL3
EXREF 77.00 ppm
BF 0.25 Hz
RGAIN 57
F
Me
OEt
O
Me
13.997
21.076
24.590
24.830
61.887
76.687
77.000
77.204
77.321
93.673
95.518
124.511
124.592
129.112
136.314
136.540
138.429
138.443
170.900
171.170
TEN041261C
PPM
0
25
50
75
100
125
150
175
200

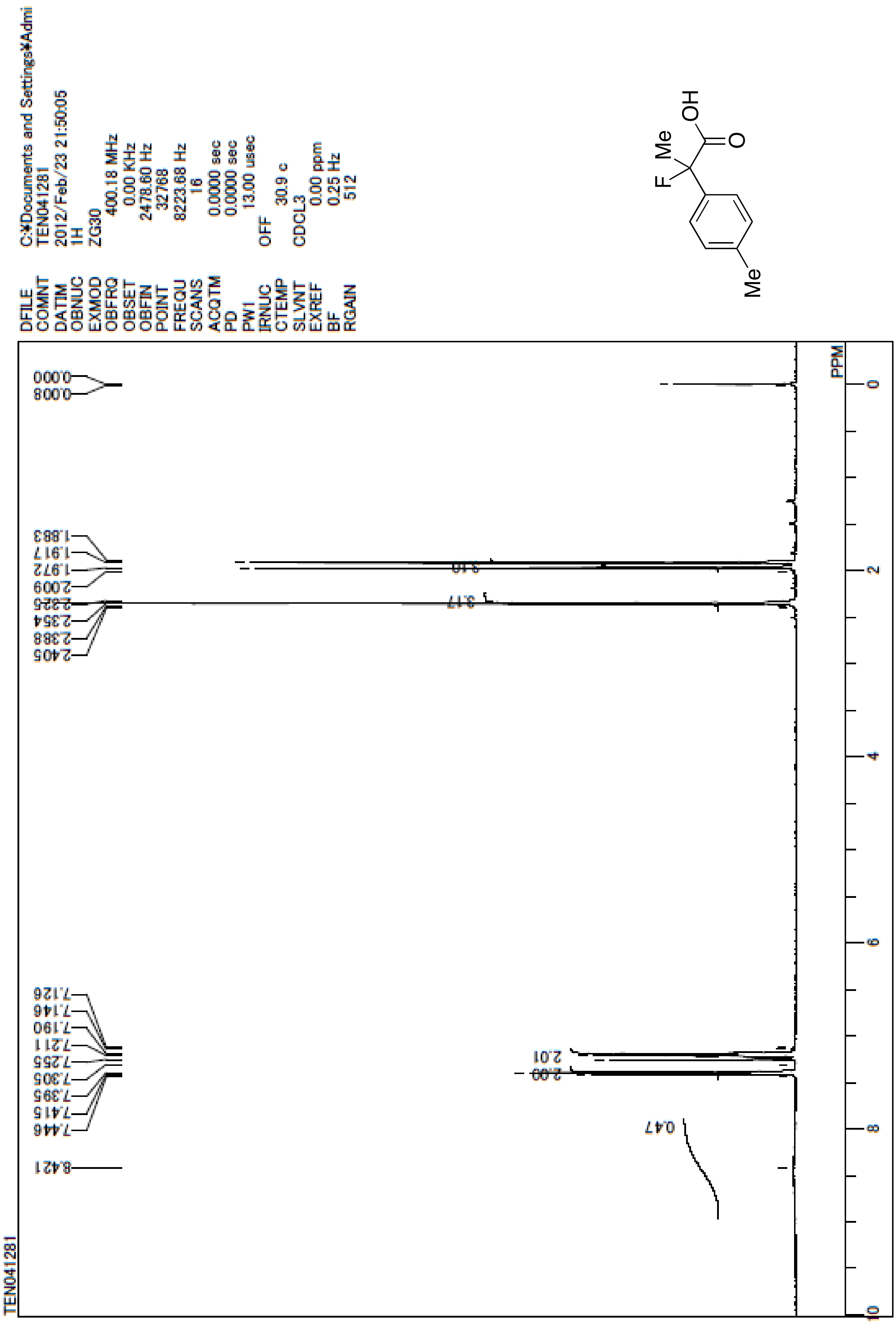
DFILE C:¥Documents and Settings¥Admi
COMNT TEN041281
DATIM 2012/Feb/23 21:50:05
OBNUC 1H
EXMOD ZG30
OBFRQ 400.18 MHz
OBSET 0.00 KHz
OBFIN 2478.60 Hz
POINT 32768
FREQU 8223.68 Hz
SCANS 16
ACQTM 0.0000 sec
PD 0.0000 sec
PW1 13.00 usec
IRNUC OFF
CTEMP 30.9 c
SLVNT CDCL3
EXREF 0.00 ppm
BF 0.25 Hz
RGAIN 512
TEN041281
PPM

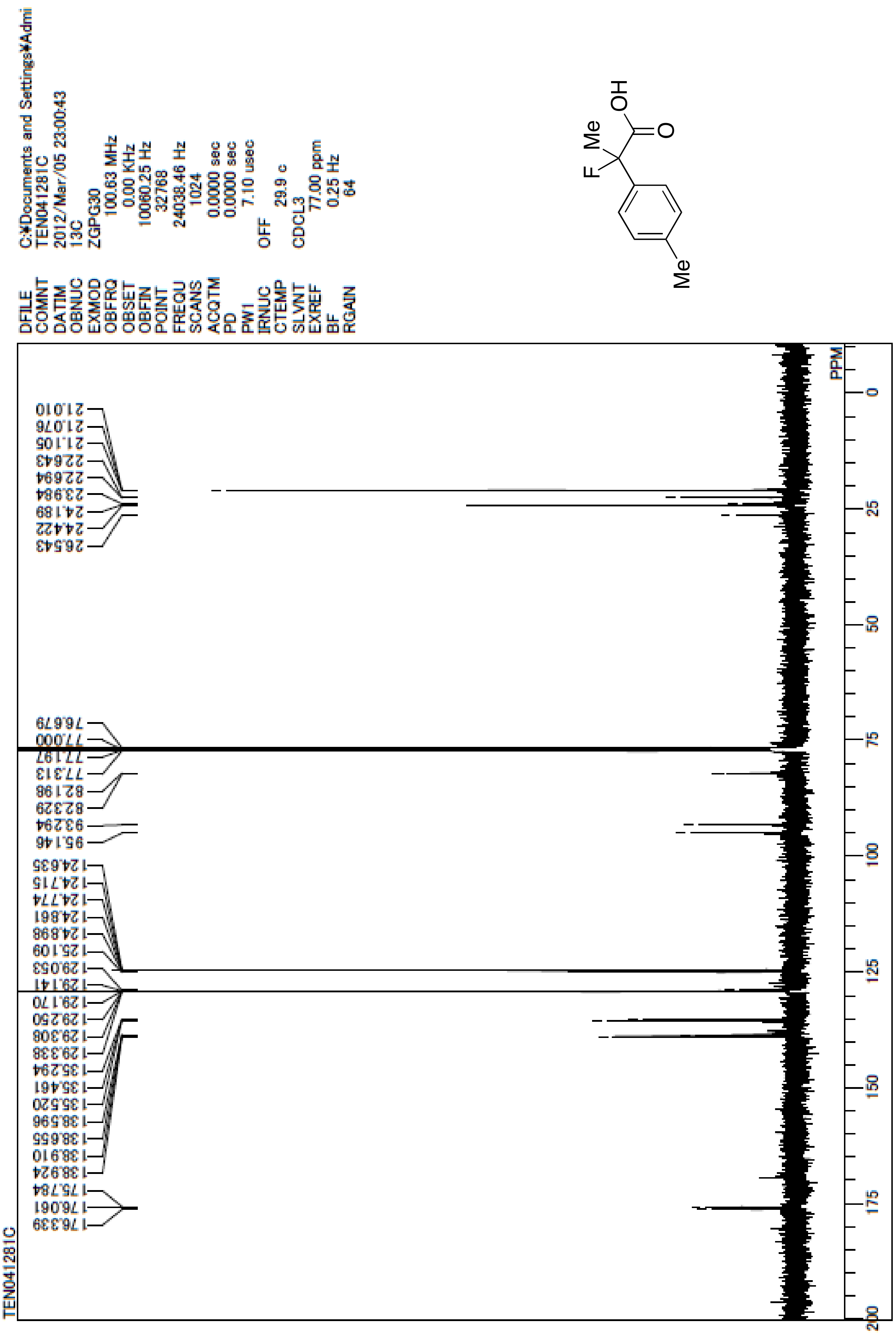

DFILE C:¥Documents and Settings¥Admi
COMNT TEN041281C
DATIM 2012/Mar/05 23:00:43
OBNUC 13C
EXMOD ZGPG30
OBFRQ 100.63 MHz
OBSET 0.00 KHz
OBFIN 10060.25 Hz
POINT 32768
FREQU 24038.46 Hz
SCANS 1024
ACQTM 0.0000 sec
PD 0.0000 sec
PW1 7.10 usec
IRNUC OFF
CTEMP 29.9 c
SLVNT CDCL3
EXREF 77.00 ppm
BF 0.25 Hz
RGAIN 64
TEN041281C
F
Me
OH
O
Me
PPM

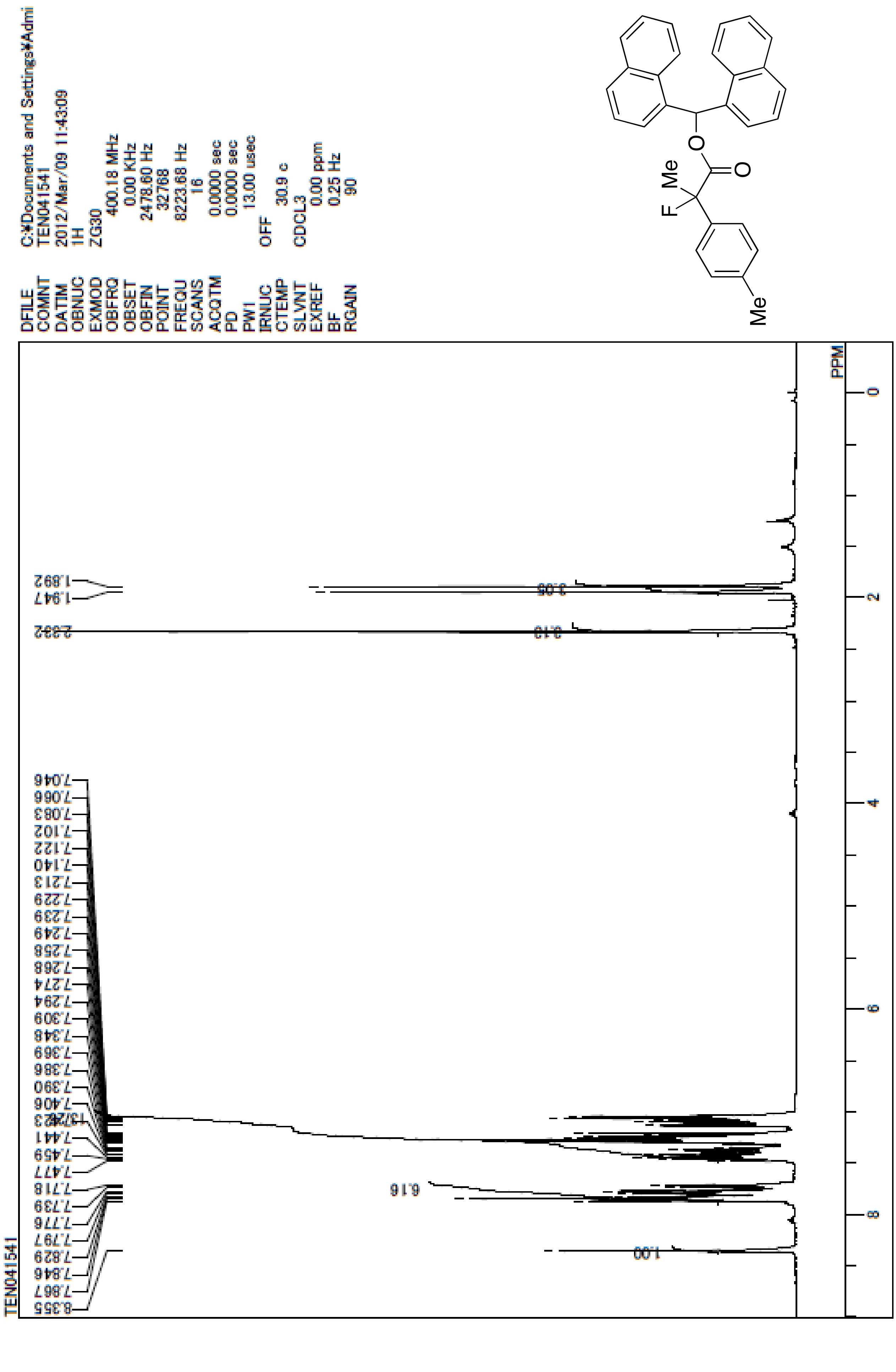

DFILE C:¥Documents and Settings¥Admi
COMNT TEN041541
DATIM 2012/Mar/09 11:43:09
OBNUC 1H
EXMOD ZG30
OBFRQ 400.18 MHz
OBSET 0.00 KHz
OBFIN 2478.60 Hz
POINT 32768
FREQU 8223.68 Hz
SCANS 16
ACQTM 0.0000 sec
PD 0.0000 sec
PW1 13.00 usec
IRNUC OFF
CTEMP 30.9 c
SLVNT CDCL3
EXREF 0.00 ppm
BF 0.25 Hz
RGAIN 90
TEN041541
PPM
F
Me
O
O
Me

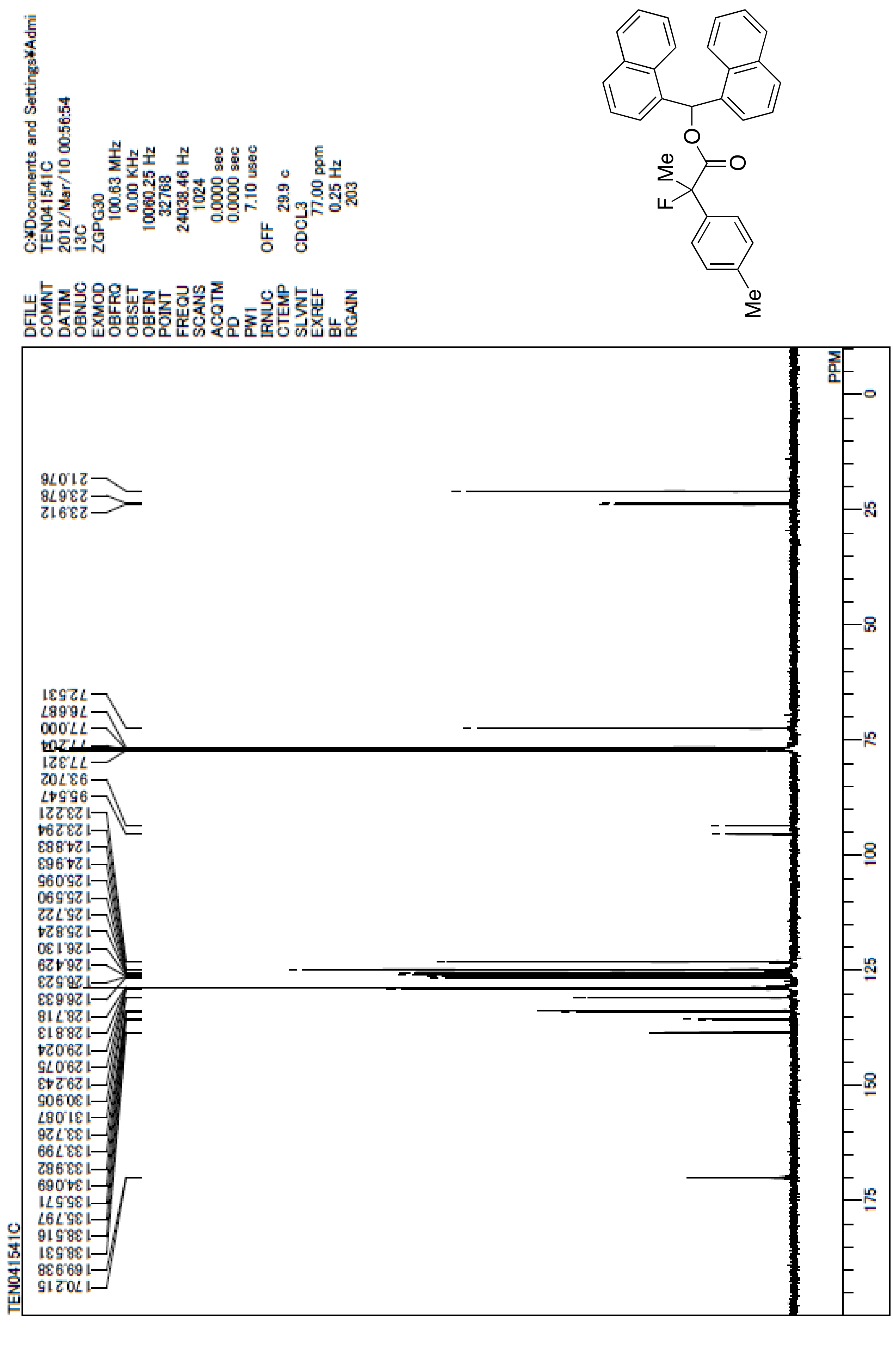
DFILE C:¥Documents and Settings¥Admi
COMNT TEN041541C
DATIM 2012/Mar/10 00:56:54
OBNUC 13C
EXMOD ZGPG30
OBFRQ 100.63 MHz
OBSET 0.00 KHz
OBFIN 10060.25 Hz
POINT 32768
FREQU 24038.46 Hz
SCANS 1024
ACQTM 0.0000 sec
PD 0.0000 sec
PW1 7.10 usec
IRNUC OFF
CTEMP 29.9 c
SLVNT CDCL3
EXREF 77.00 ppm
BF 0.25 Hz
RGAIN 203
TEN041541C
PPM
F
Me
O
O
Me

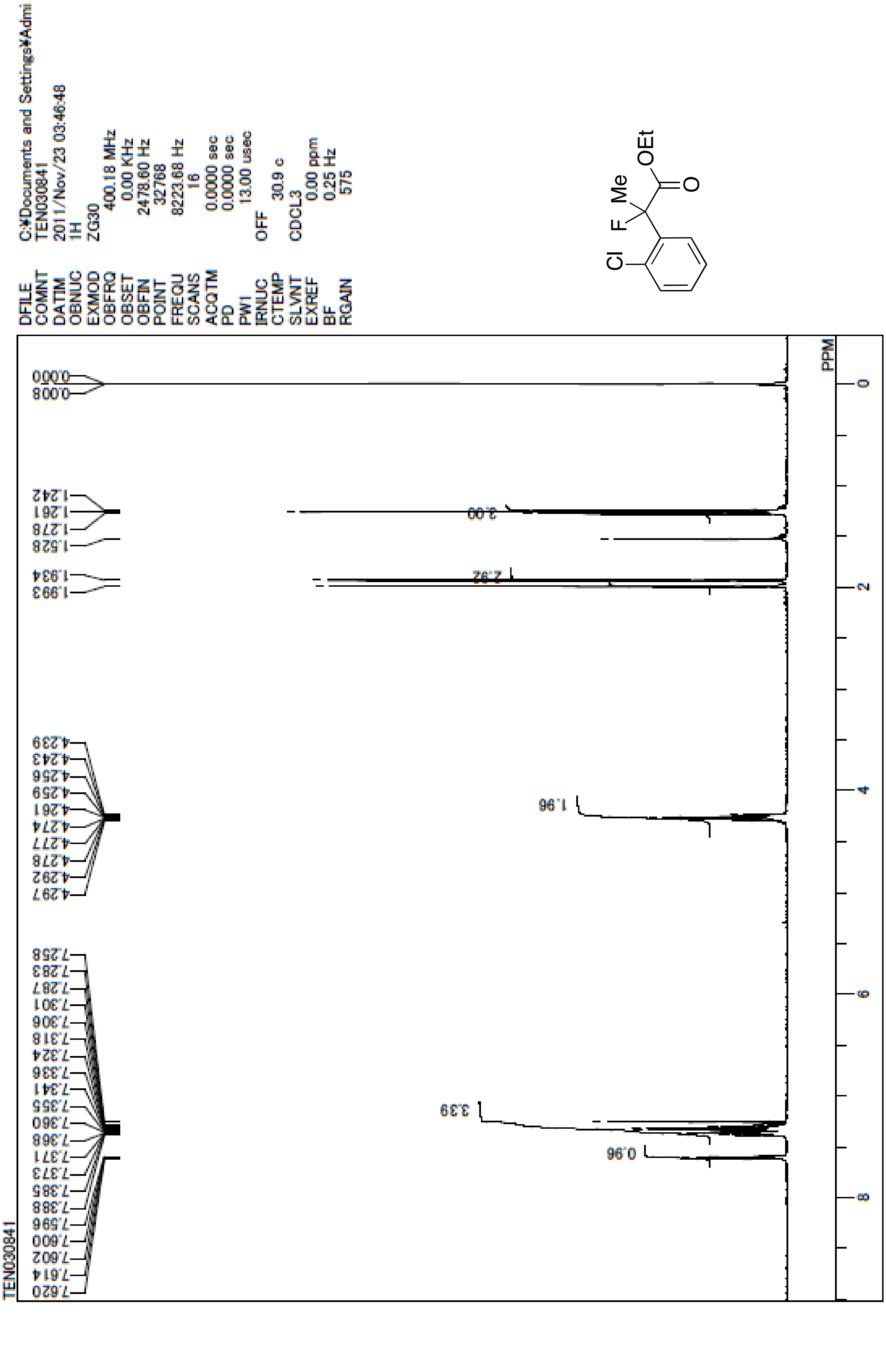
DFILE C:¥Documents and Settings¥Admi
COMNT TEN030841
DATIM 2011/Nov/23 03:46:48
OBNUC 1H
EXMOD ZG30
OBFRQ 400.18 MHz
OBSET 0.00 KHz
OBFIN 2478.60 Hz
POINT 32768
FREQU 8223.68 Hz
SCANS 16
ACQTM 0.0000 sec
PD 0.0000 sec
PW1 13.00 usec
IRNUC OFF
CTEMP 30.9 c
SLVNT CDCL3
EXREF 0.00 ppm
BF 0.25 Hz
RGAIN 575
Cl
F
Me
OEt
O
TEN030841
PPM

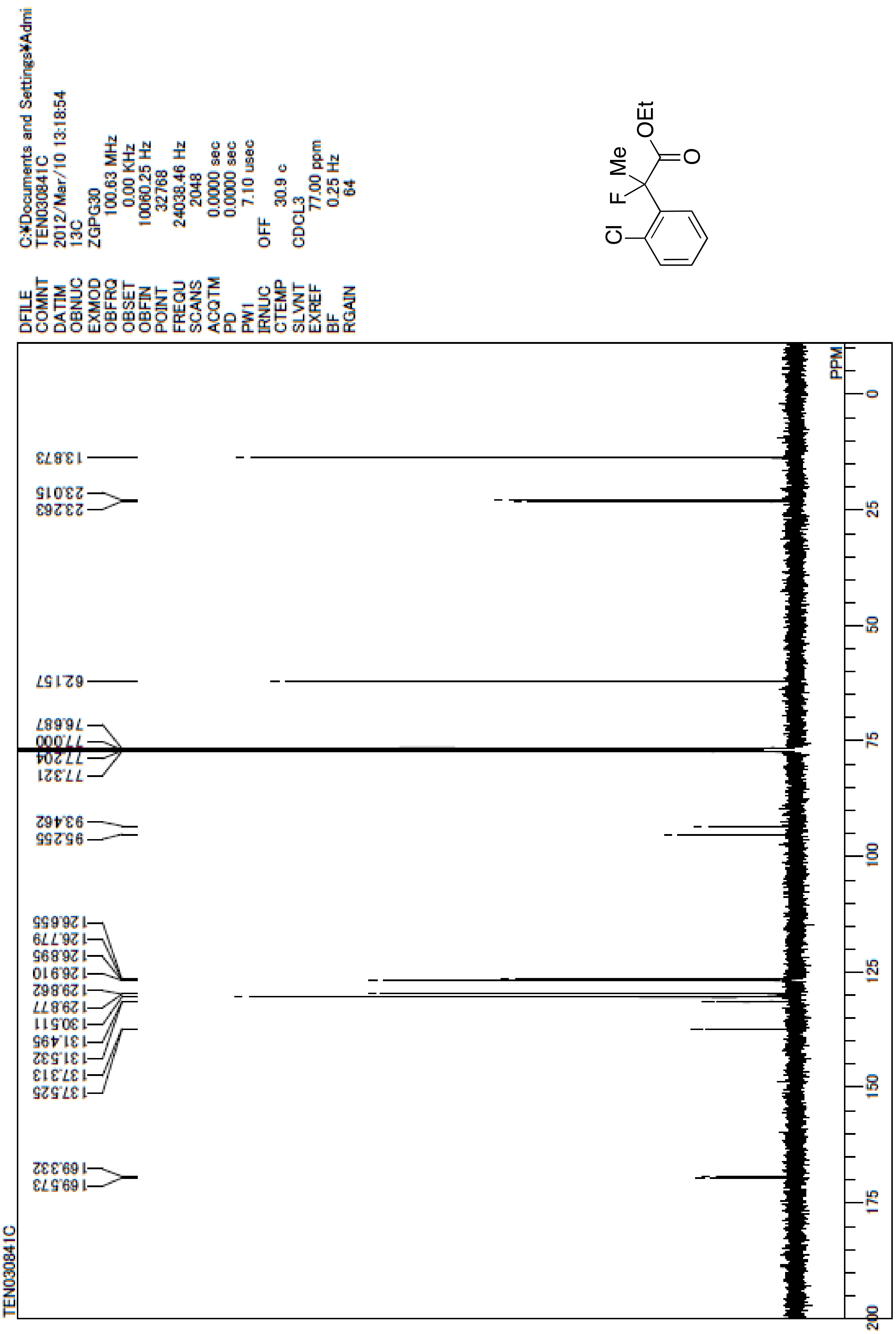
DFILE C:¥Documents and Settings¥Admi
COMNT TEN030841C
DATIM 2012/Mar/10 13:18:54
OBNUC 13C
EXMOD ZGPG30
OBFRQ 100.63 MHz
OBSET 0.00 KHz
OBFIN 10060.25 Hz
POINT 32768
FREQU 24038.46 Hz
SCANS 2048
ACQTM 0.0000 sec
PD 0.0000 sec
PW1 7.10 usec
IRNUC OFF
CTEMP 30.9 c
SLVNT CDCL3
EXREF 77.00 ppm
BF 0.25 Hz
RGAIN 64
Cl
F
Me
OEt
O
TEN030841C
13.873
23.015
23.263
62.157
76.687
77.000
77.204
77.321
93.462
95.255
126.655
126.779
126.895
126.910
129.862
129.877
130.511
131.495
131.532
137.313
137.525
169.332
169.573
PPM
0
25
50
75
100
125
150
175
200

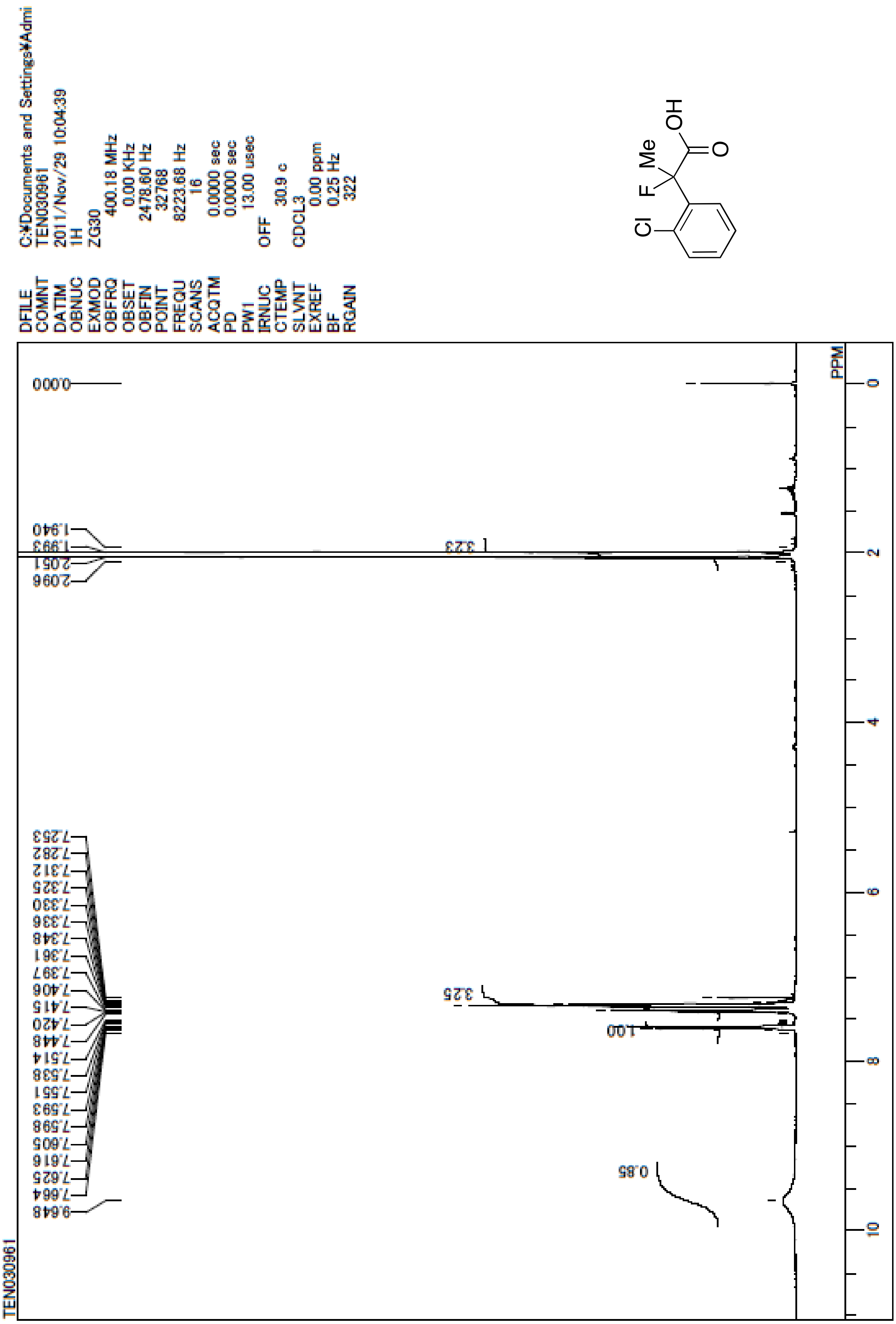
DFILE C:¥Documents and Settings¥Admi
COMNT TEN030961
DATIM 2011/Nov/29 10:04:39
OBNUC 1H
EXMOD ZG30
OBFRQ 400.18 MHz
OBSET 0.00 KHz
OBFIN 2478.60 Hz
POINT 32768
FREQU 8223.68 Hz
SCANS 16
ACQTM 0.0000 sec
PD 0.0000 sec
PW1 13.00 usec
IRNUC OFF
CTEMP 30.9 c
SLVNT CDCL3
EXREF 0.00 ppm
BF 0.25 Hz
RGAIN 322
Cl
F
Me
OH
O
TEN030961
0.000
1.940
1.993
2.051
2.096
7.253
7.282
7.312
7.325
7.330
7.336
7.348
7.361
7.397
7.406
7.415
7.420
7.448
7.514
7.538
7.551
7.593
7.598
7.605
7.616
7.625
7.664
9.648
3.23
3.25
1.00
0.85
PPM
0
2
4
6
8
10

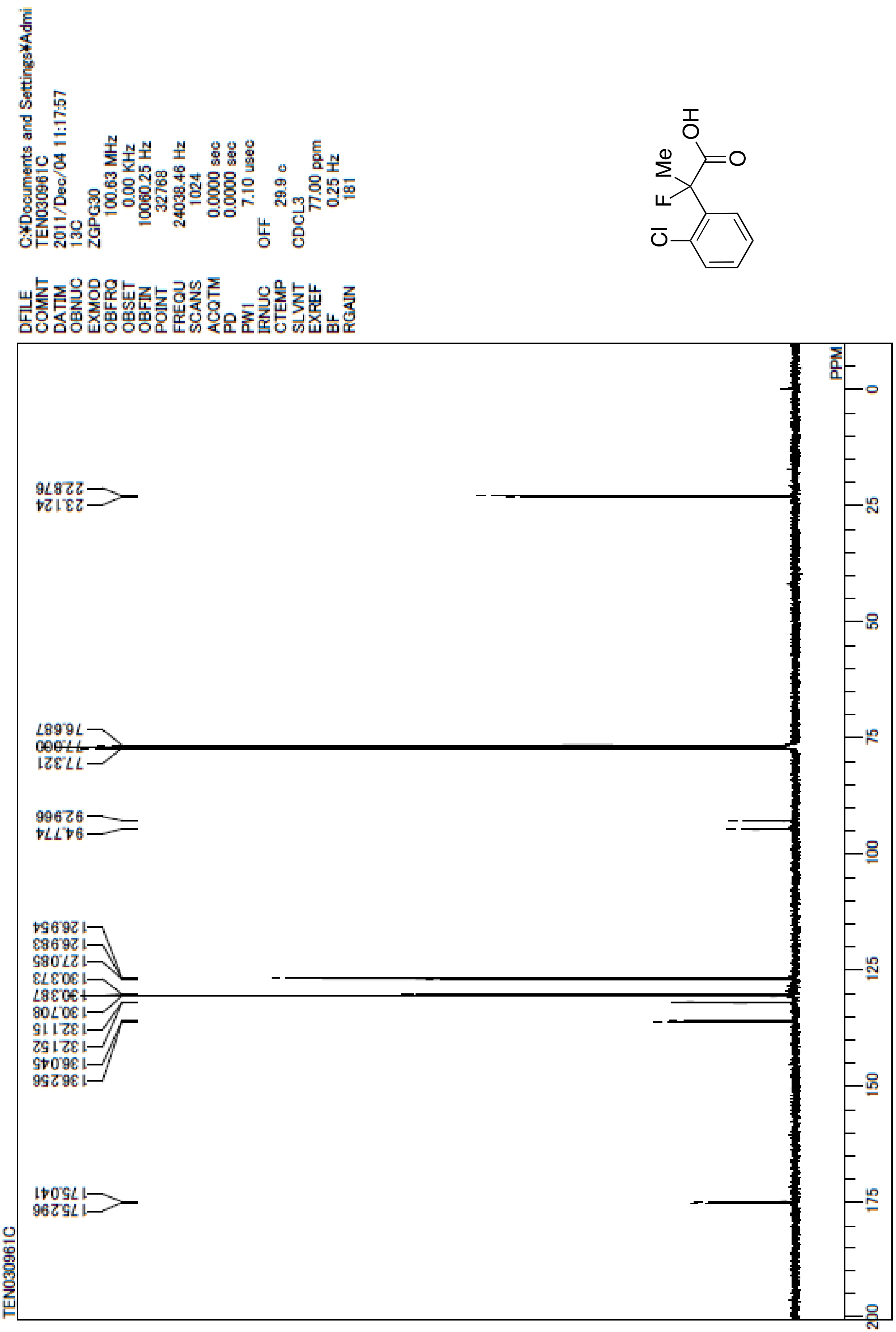
DFILE C:¥Documents and Settings¥Admi
COMNT TEN030961C
DATIM 2011/Dec/04 11:17:57
OBNUC 13C
EXMOD ZGPG30
OBFRQ 100.63 MHz
OBSET 0.00 KHz
OBFIN 10060.25 Hz
POINT 32768
FREQU 24038.46 Hz
SCANS 1024
ACQTM 0.0000 sec
PD 0.0000 sec
PW1 7.10 usec
IRNUC OFF
CTEMP 29.9 c
SLVNT CDCL3
EXREF 77.00 ppm
BF 0.25 Hz
RGAIN 181
Cl
F
Me
OH
O
TEN030961C
22.876
23.124
76.687
77.000
77.321
92.966
94.774
126.954
126.983
127.085
130.373
130.387
130.708
132.115
132.152
136.045
136.256
175.041
175.296
PPM
0
25
50
75
100
125
150
175
200

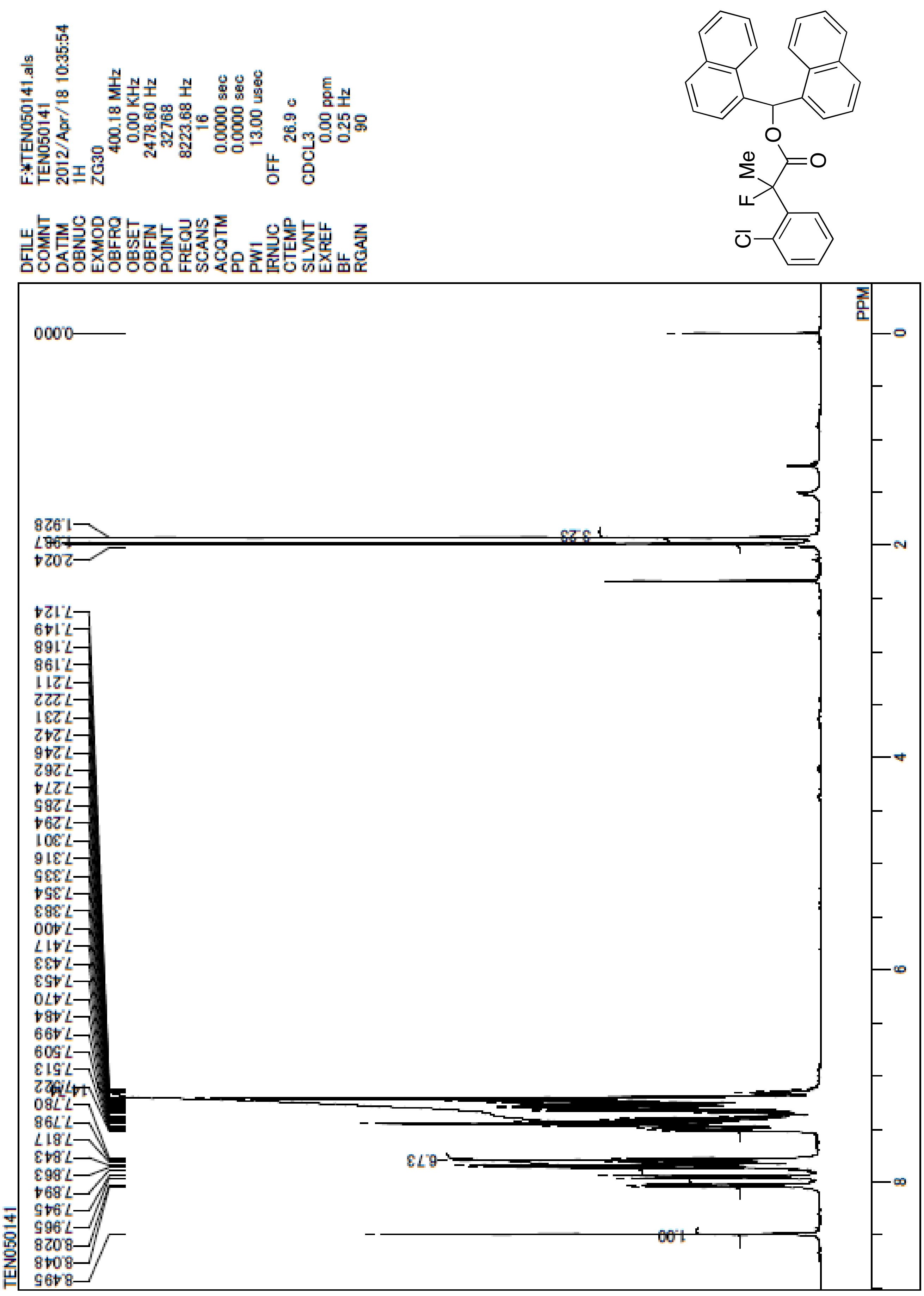
DFILE F:¥TEN050141.als
COMNT TEN050141
DATIM 2012/Apr/18 10:35:54
OBNUC 1H
EXMOD ZG30
OBFRQ 400.18 MHz
OBSET 0.00 KHz
OBFIN 2478.60 Hz
POINT 32768
FREQU 8223.68 Hz
SCANS 16
ACQTM 0.0000 sec
PD 0.0000 sec
PW1 13.00 usec
IRNUC OFF
CTEMP 26.9 c
SLVNT CDCL3
EXREF 0.00 ppm
BF 0.25 Hz
RGAIN 90
Cl
F
Me
O
O
TEN050141
0.000
1.928
1.967
2.024
7.124
7.149
7.168
7.198
7.211
7.222
7.231
7.242
7.246
7.262
7.274
7.285
7.294
7.301
7.316
7.335
7.354
7.383
7.400
7.417
7.433
7.453
7.470
7.484
7.499
7.509
7.513
7.780
7.798
7.817
7.843
7.863
7.894
7.945
7.965
8.028
8.048
8.495
3.23
6.73
1.00
PPM
0
2
4
6
8

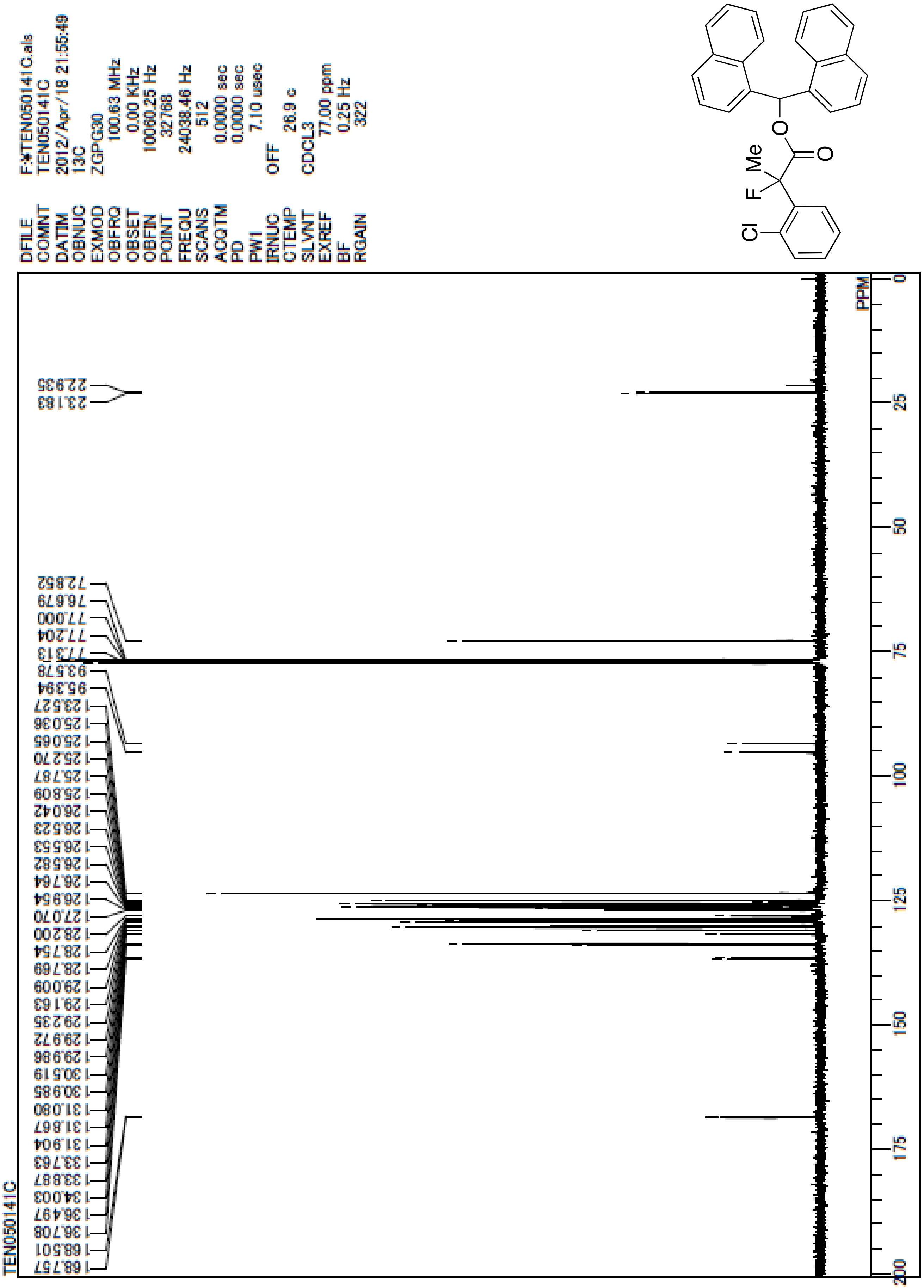
DFILE F:¥TEN050141C.als
COMNT TEN050141C
DATIM 2012/Apr/18 21:55:49
OBNUC 13C
EXMOD ZGPG30
OBFRQ 100.63 MHz
OBSET 0.00 KHz
OBFIN 10060.25 Hz
POINT 32768
FREQU 24038.46 Hz
SCANS 512
ACQTM 0.0000 sec
PD 0.0000 sec
PW1 7.10 usec
IRNUC OFF
CTEMP 26.9 c
SLVNT CDCL3
EXREF 77.00 ppm
BF 0.25 Hz
RGAIN 322
TEN050141C
22.935
23.183
72.852
76.679
77.000
77.204
77.313
93.578
95.394
123.527
125.036
125.065
125.270
125.787
125.809
126.042
126.523
126.553
126.582
126.764
126.954
127.070
128.200
128.754
128.769
129.009
129.163
129.235
129.972
129.986
130.519
130.985
131.080
131.867
131.904
133.763
133.887
134.003
136.497
136.708
168.501
168.757
PPM
0
25
50
75
100
125
150
175
200
Cl
F
Me
O
O

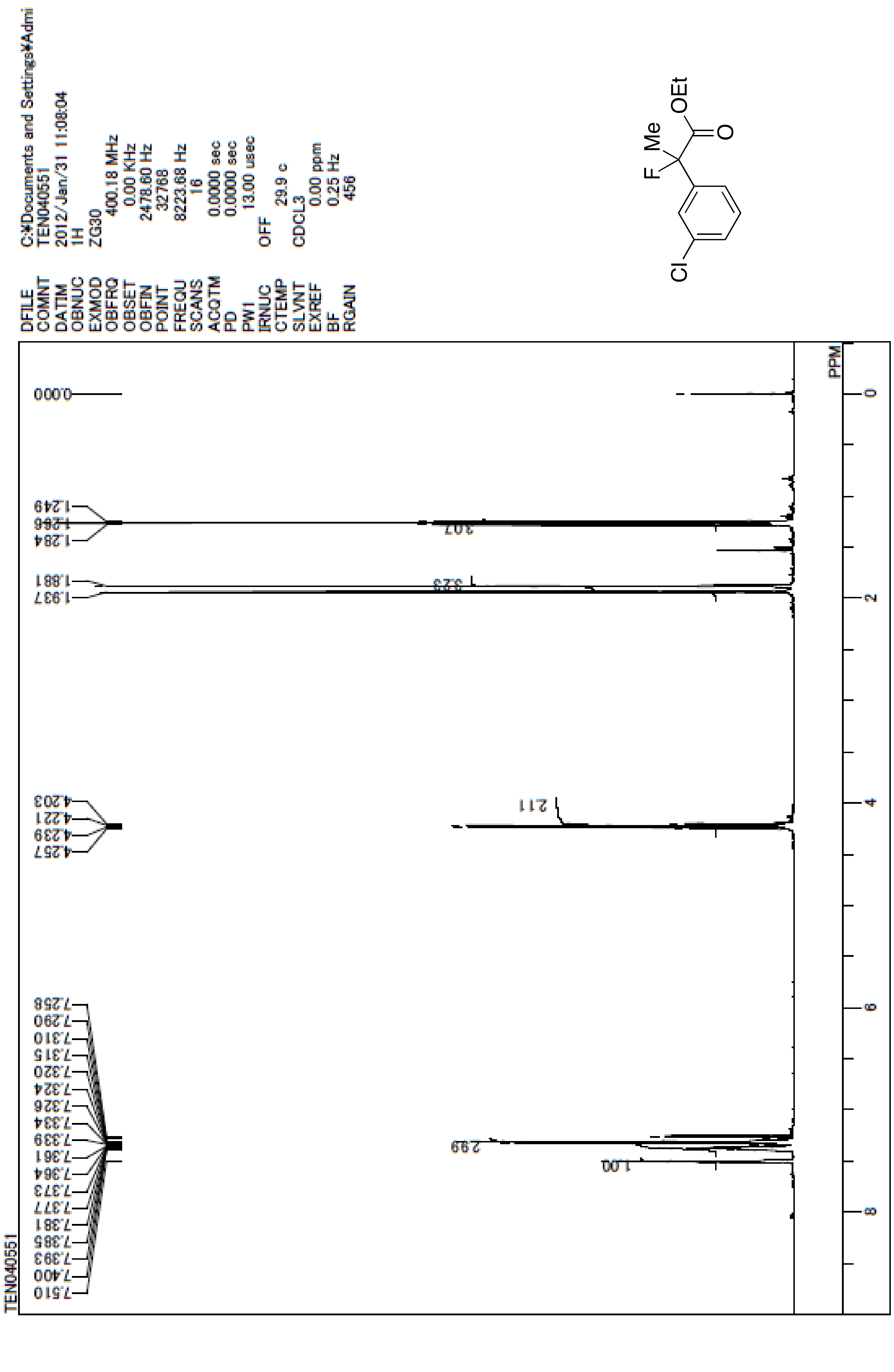
DFILE C:¥Documents and Settings¥Admi
COMNT TEN040551
DATIM 2012/Jan/31 11:08:04
OBNUC 1H
EXMOD ZG30
OBFRQ 400.18 MHz
OBSET 0.00 KHz
OBFIN 2478.60 Hz
POINT 32768
FREQU 8223.68 Hz
SCANS 16
ACQTM 0.0000 sec
PD 0.0000 sec
PW1 13.00 usec
IRNUC OFF
CTEMP 29.9 c
SLVNT CDCL3
EXREF 0.00 ppm
BF 0.25 Hz
RGAIN 456
F
Me
OEt
O
Cl
TEN040551
0.000
1.249
1.266
1.284
1.881
1.937
4.203
4.221
4.239
4.257
7.258
7.290
7.310
7.315
7.320
7.324
7.326
7.334
7.339
7.361
7.364
7.373
7.377
7.381
7.385
7.393
7.400
7.510
3.07
3.23
2.11
2.99
1.00
PPM
0
2
4
6
8

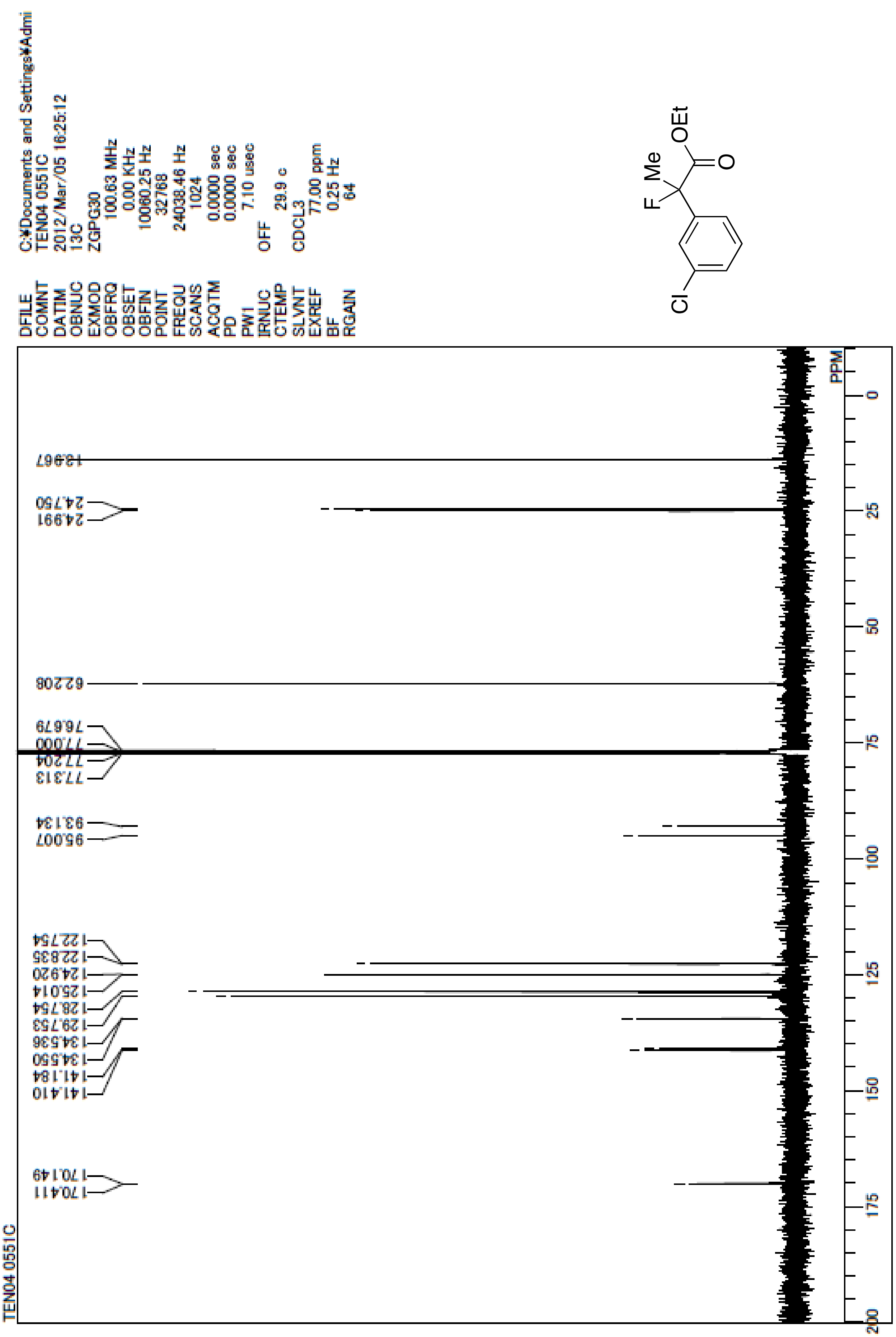

DFILE C:¥Documents and Settings¥Admi
COMNT TEN04 0551C
DATIM 2012/Mar/05 16:25:12
OBNUC 13C
EXMOD ZGPG30
OBFRQ 100.63 MHz
OBSET 0.00 KHz
OBFIN 10060.25 Hz
POINT 32768
FREQU 24038.46 Hz
SCANS 1024
ACQTM 0.0000 sec
PD 0.0000 sec
PW1 7.10 usec
IRNUC OFF
CTEMP 29.9 c
SLVNT CDCL3
EXREF 77.00 ppm
BF 0.25 Hz
RGAIN 64
Cl
F
Me
OEt
O
TEN04 0551C
13.967
24.750
24.991
62.208
76.679
77.000
77.204
77.313
93.134
95.007
122.754
122.835
124.920
125.014
128.754
129.753
134.536
134.550
141.184
141.410
170.149
170.411
PPM
0
25
50
75
100
125
150
175
200

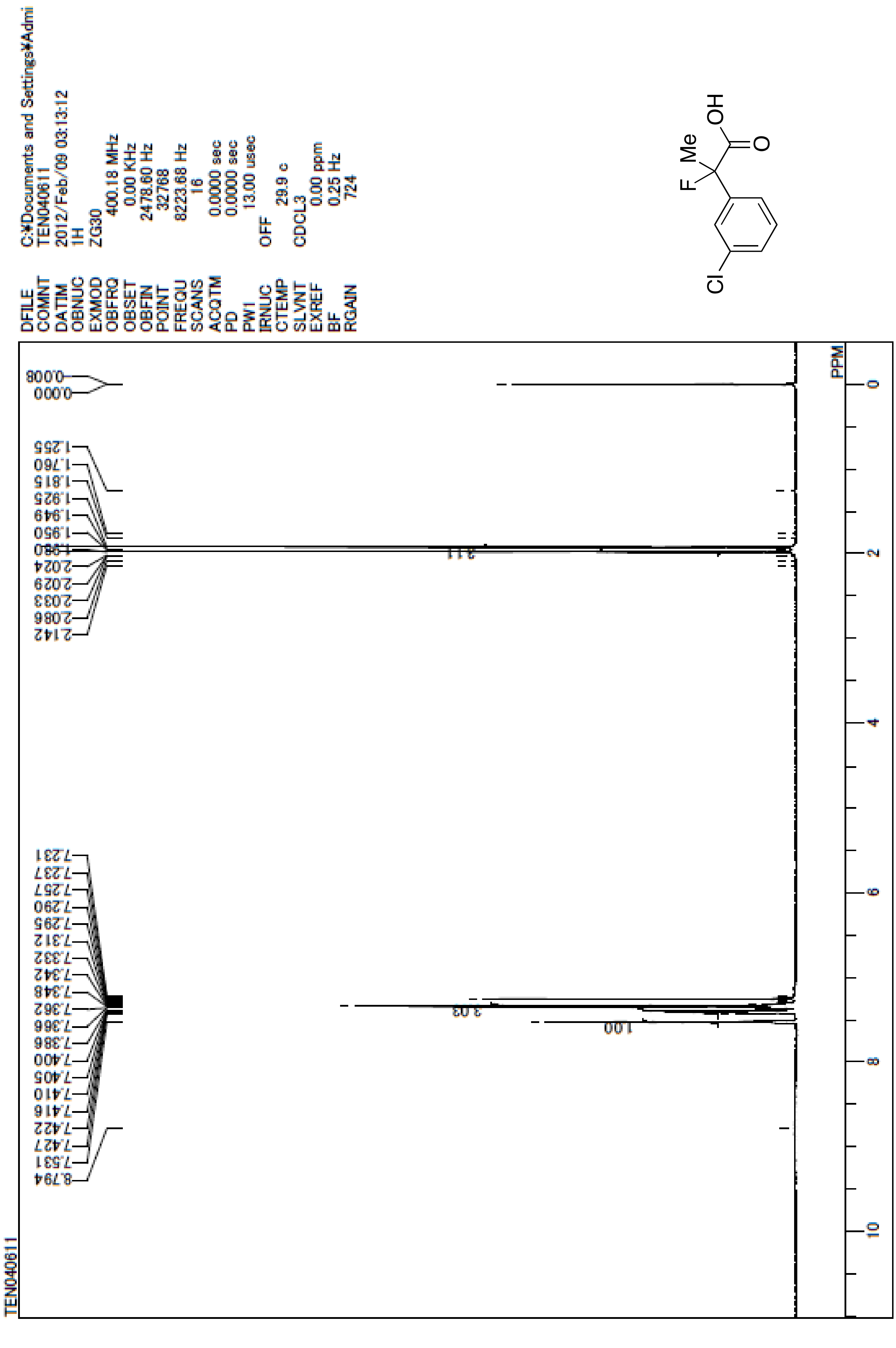
DFILE C:¥Documents and Settings¥Admi
COMNT TEN040611
DATIM 2012/Feb/09 03:13:12
OBNUC 1H
EXMOD ZG30
OBFRQ 400.18 MHz
OBSET 0.00 KHz
OBFIN 2478.60 Hz
POINT 32768
FREQU 8223.68 Hz
SCANS 16
ACQTM 0.0000 sec
PD 0.0000 sec
PW1 13.00 usec
IRNUC OFF
CTEMP 29.9 c
SLVNT CDCL3
EXREF 0.00 ppm
BF 0.25 Hz
RGAIN 724
TEN040611
PPM
F
Me
OH
O
Cl

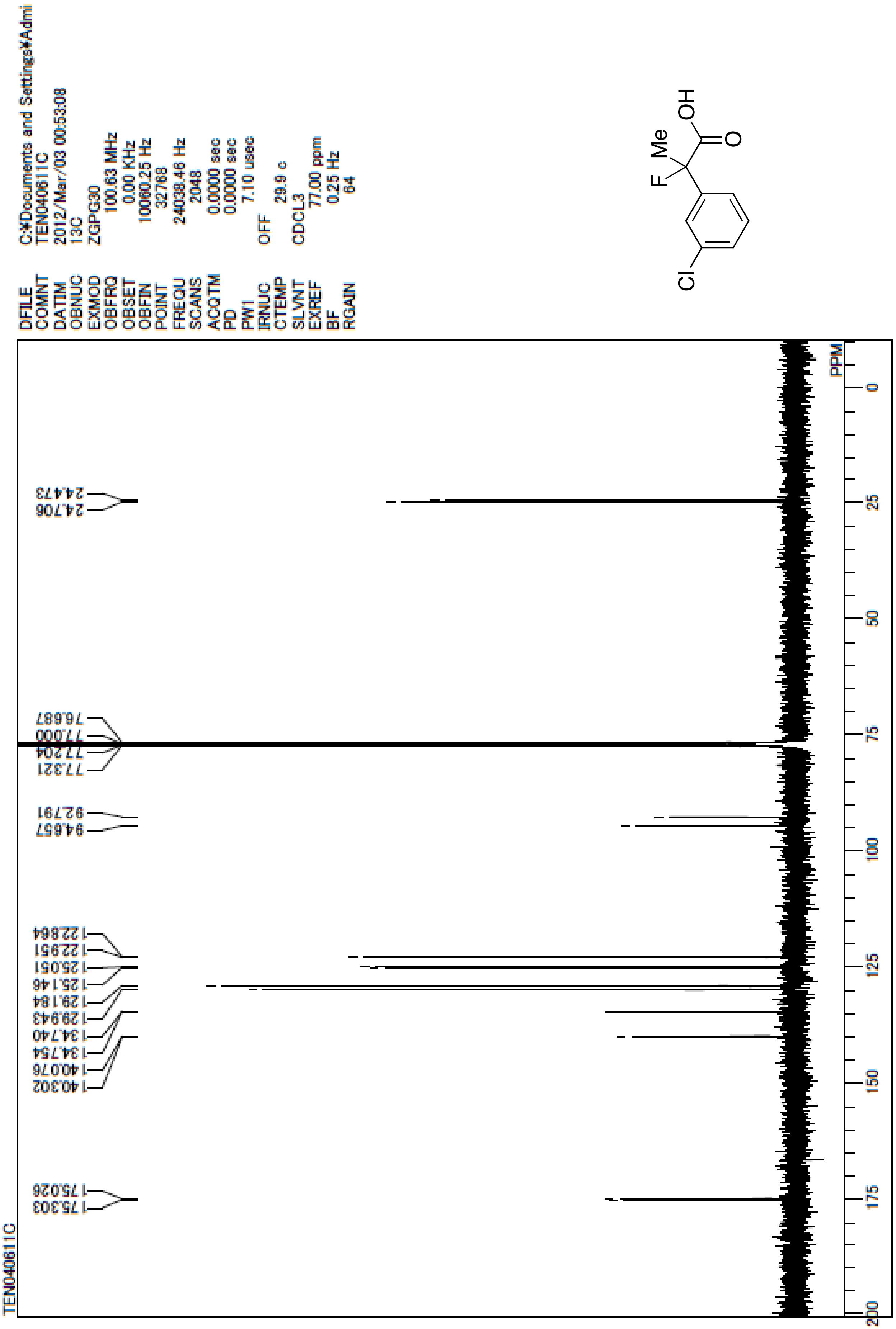
DFILE C:¥Documents and Settings¥Admi
COMNT TEN040611C
DATIM 2012/Mar/03 00:53:08
OBNUC 13C
EXMOD ZGPG30
OBFRQ 100.63 MHz
OBSET 0.00 KHz
OBFIN 10060.25 Hz
POINT 32768
FREQU 24038.46 Hz
SCANS 2048
ACQTM 0.0000 sec
PD 0.0000 sec
PW1 7.10 usec
IRNUC OFF
CTEMP 29.9 c
SLVNT CDCL3
EXREF 77.00 ppm
BF 0.25 Hz
RGAIN 64
F
Me
OH
O
Cl
24.473
24.706
76.687
77.000
77.204
77.321
92.791
94.657
122.864
122.951
125.051
125.146
129.184
129.943
134.740
134.754
140.076
140.302
175.026
175.303
TEN040611C
PPM
0
25
50
75
100
125
150
175
200

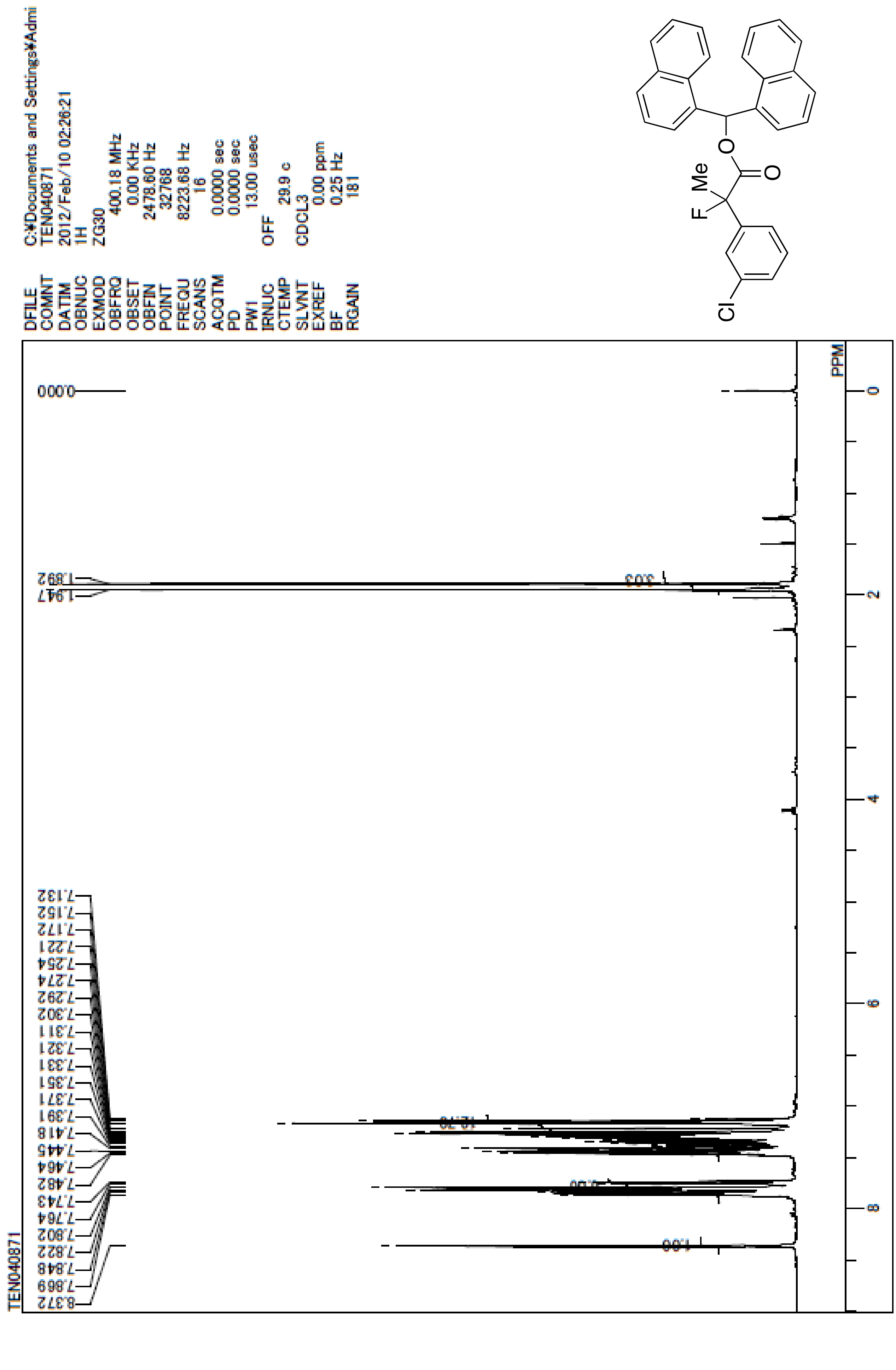
DFILE C:¥Documents and Settings¥Admi
COMNT TEN040871
DATIM 2012/Feb/10 02:26:21
OBNUC 1H
EXMOD ZG30
OBFRQ 400.18 MHz
OBSET 0.00 KHz
OBFIN 2478.60 Hz
POINT 32768
FREQU 8223.68 Hz
SCANS 16
ACQTM 0.0000 sec
PD 0.0000 sec
PW1 13.00 usec
IRNUC OFF
CTEMP 29.9 c
SLVNT CDCL3
EXREF 0.00 ppm
BF 0.25 Hz
RGAIN 181
TEN040871
0.000
1.892
1.947
7.132
7.152
7.172
7.221
7.254
7.274
7.292
7.302
7.311
7.321
7.331
7.351
7.371
7.391
7.418
7.445
7.464
7.482
7.743
7.764
7.802
7.822
7.848
7.869
8.372
3.03
12.70
1.00
PPM
Cl
F
Me
O
O

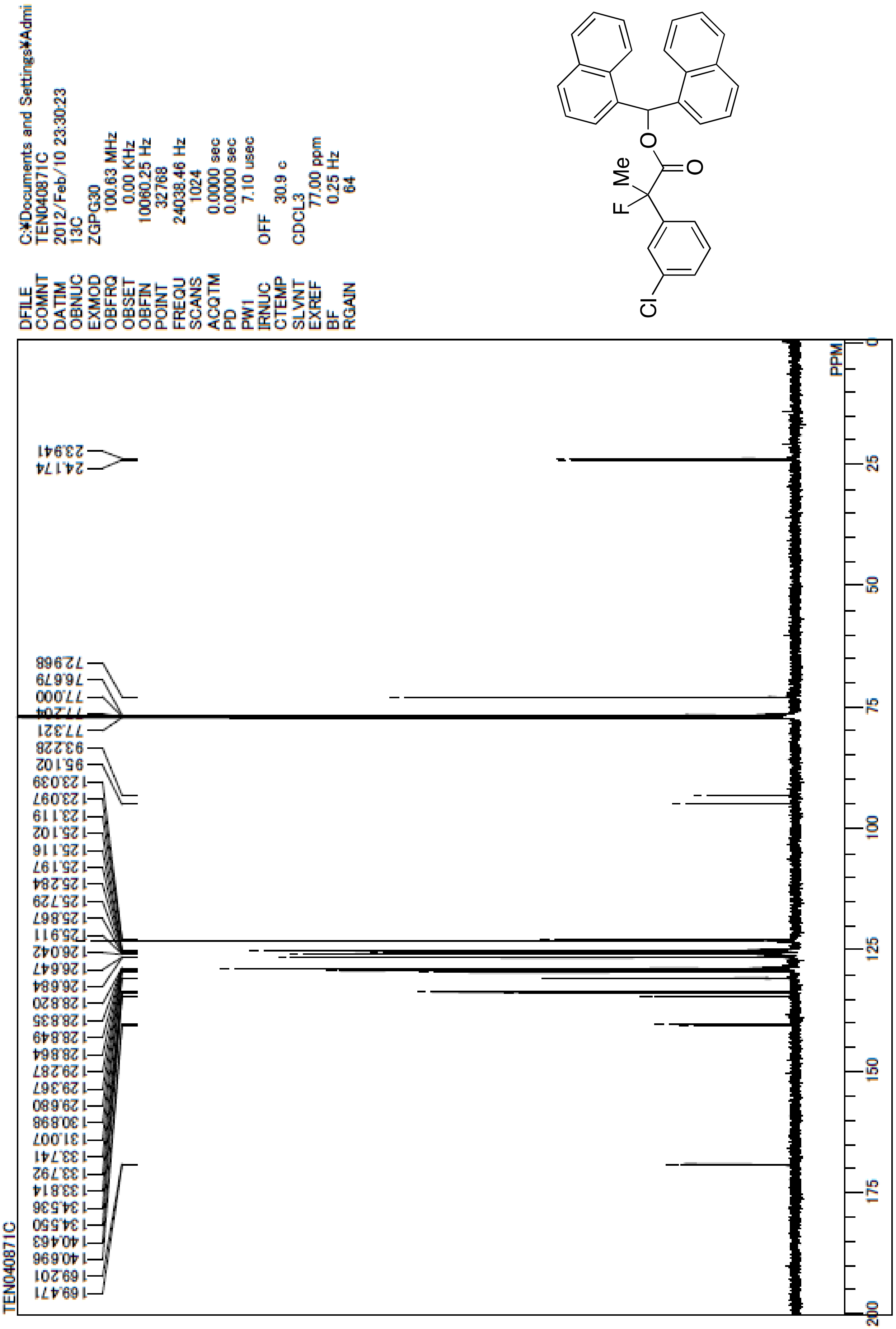
DFILE C:¥Documents and Settings¥Admi
COMNT TEN040871C
DATIM 2012/Feb/10 23:30:23
OBNUC 13C
EXMOD ZGPG30
OBFRQ 100.63 MHz
OBSET 0.00 KHz
OBFIN 10060.25 Hz
POINT 32768
FREQU 24038.46 Hz
SCANS 1024
ACQTM 0.0000 sec
PD 0.0000 sec
PW1 7.10 usec
IRNUC OFF
CTEMP 30.9 c
SLVNT CDCL3
EXREF 77.00 ppm
BF 0.25 Hz
RGAIN 64
F
Me
O
O
Cl
TEN040871C
23.941
24.174
72.968
76.679
77.000
77.204
77.321
93.228
95.102
123.039
123.097
123.119
125.102
125.116
125.197
125.284
125.729
125.867
125.911
126.042
126.647
126.684
128.820
128.835
128.849
128.864
129.287
129.367
129.680
130.898
131.007
133.741
133.792
133.814
134.536
134.550
140.463
140.696
169.201
169.471
PPM
0
25
50
75
100
125
150
175
200

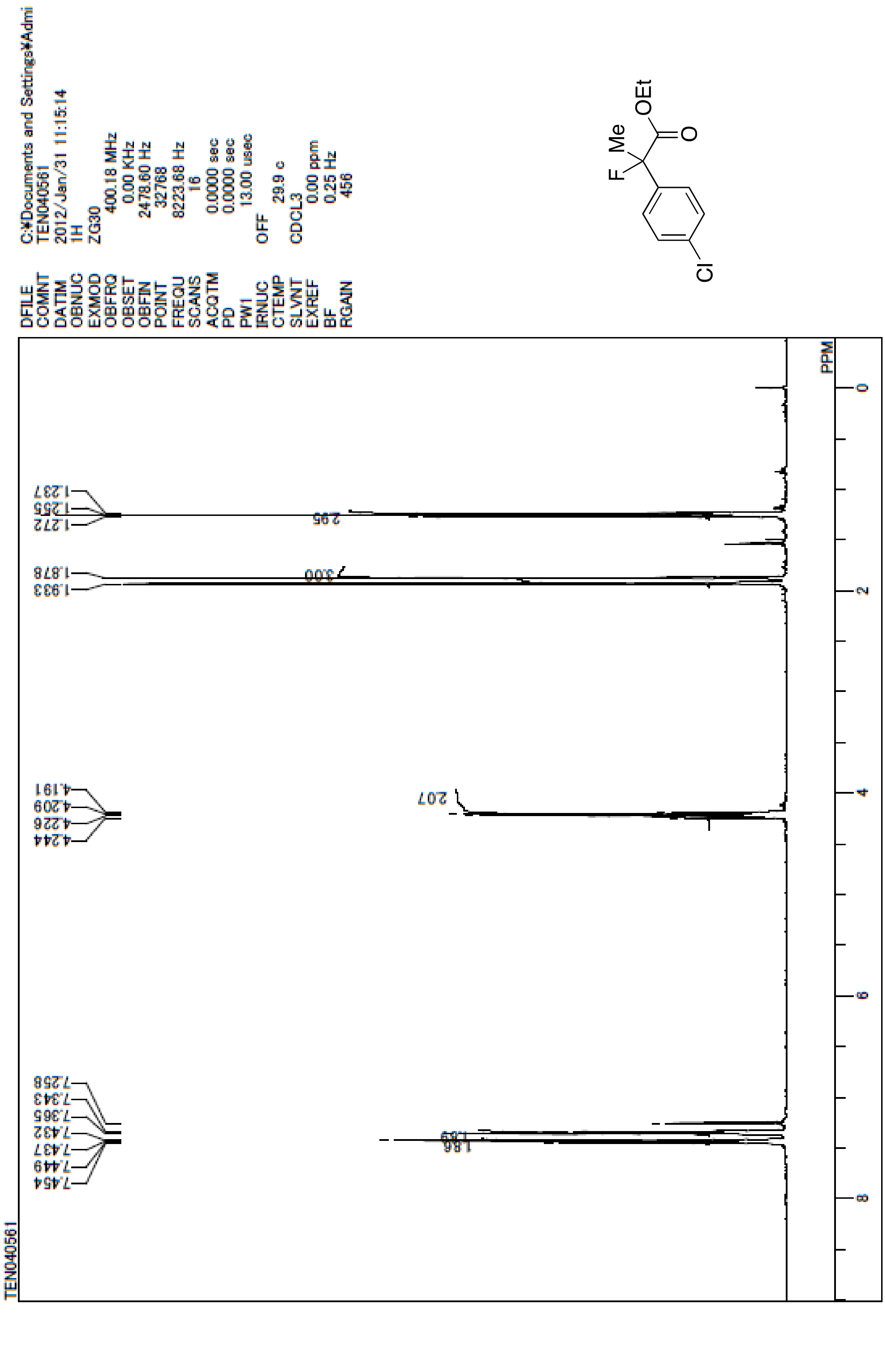
DFILE C:¥Documents and Settings¥Admi
COMNT TEN040561
DATIM 2012/Jan/31 11:15:14
OBNUC 1H
EXMOD ZG30
OBFRQ 400.18 MHz
OBSET 0.00 KHz
OBFIN 2478.60 Hz
POINT 32768
FREQU 8223.68 Hz
SCANS 16
ACQTM 0.0000 sec
PD 0.0000 sec
PW1 13.00 usec
IRNUC OFF
CTEMP 29.9 c
SLVNT CDCL3
EXREF 0.00 ppm
BF 0.25 Hz
RGAIN 456
F
Me
OEt
O
Cl
TEN040561
PPM
0
2
4
6
8
1.237
1.255
1.272
1.878
1.933
4.191
4.209
4.226
4.244
7.258
7.343
7.365
7.432
7.437
7.449
7.454
2.95
3.00
2.07
1.89
1.86

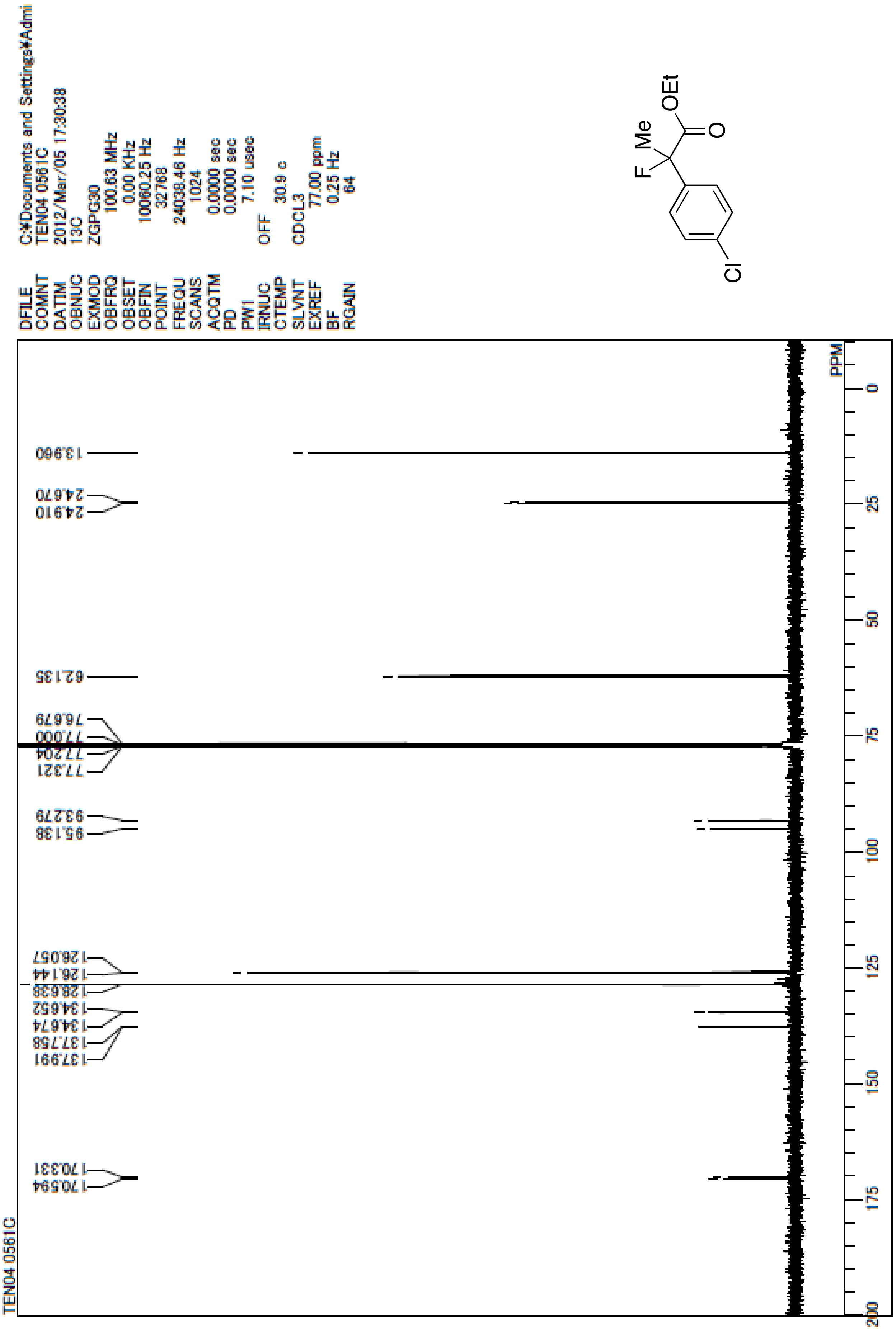
TEN04 0561C
DFILE C:¥Documents and Settings¥Admi
COMNT TEN04 0561C
DATIM 2012/Mar/05 17:30:38
OBNUC 13C
EXMOD ZGPG30
OBFRQ 100.63 MHz
OBSET 0.00 KHz
OBFIN 10060.25 Hz
POINT 32768
FREQU 24038.46 Hz
SCANS 1024
ACQTM 0.0000 sec
PD 0.0000 sec
PW1 7.10 usec
IRNUC OFF
CTEMP 30.9 c
SLVNT CDCL3
EXREF 77.00 ppm
BF 0.25 Hz
RGAIN 64
13.960
24.670
24.910
62.135
76.679
77.000
77.204
77.321
93.279
95.138
126.057
126.144
128.638
134.652
134.674
137.758
137.991
170.331
170.594
PPM
0
25
50
75
100
125
150
175
200
F
Me
OEt
O
Cl

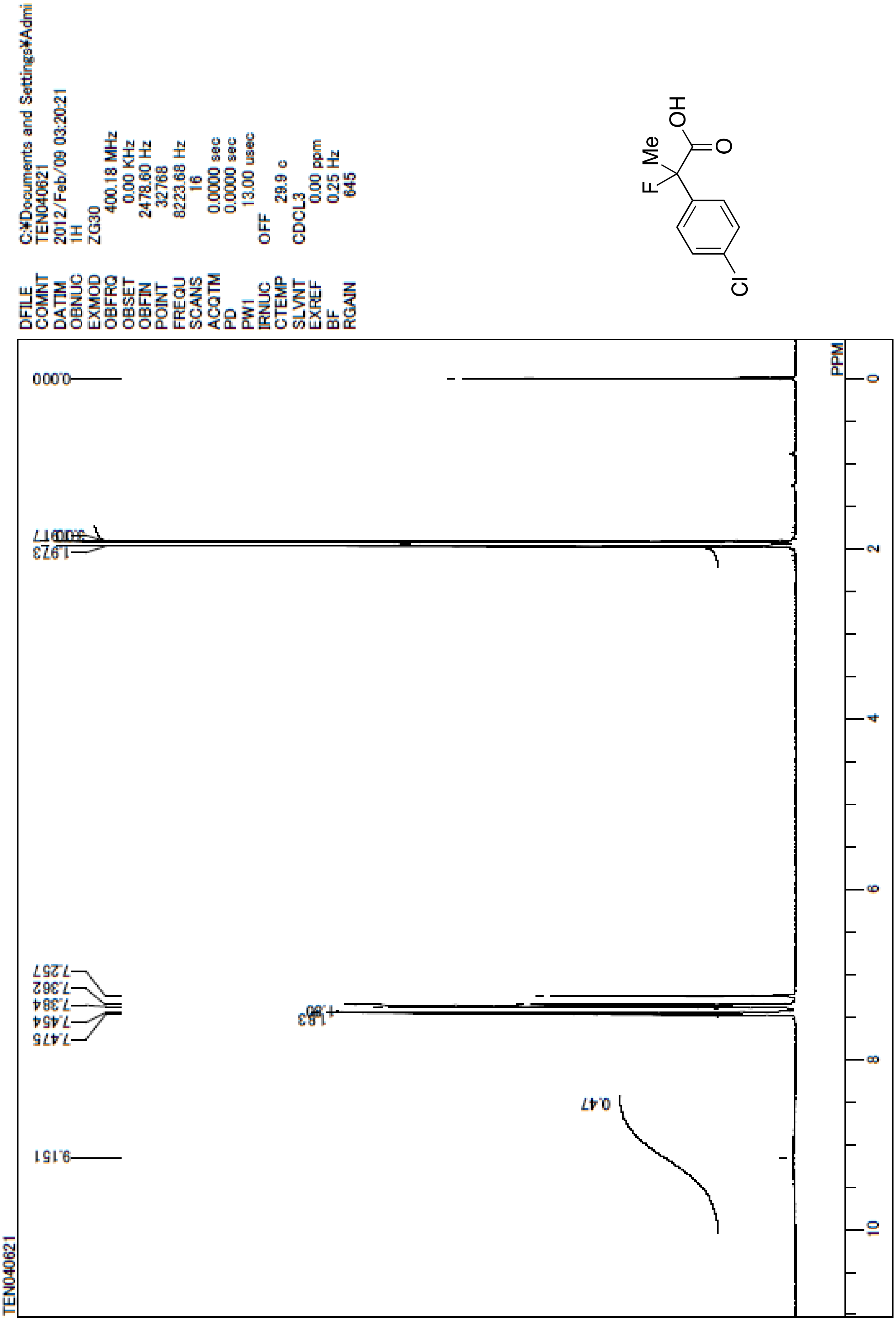
DFILE C:¥Documents and Settings¥Admi
COMNT TEN040621
DATIM 2012/Feb/09 03:20:21
OBNUC 1H
EXMOD ZG30
OBFRQ 400.18 MHz
OBSET 0.00 KHz
OBFIN 2478.60 Hz
POINT 32768
FREQU 8223.68 Hz
SCANS 16
ACQTM 0.0000 sec
PD 0.0000 sec
PW1 13.00 usec
IRNUC OFF
CTEMP 29.9 c
SLVNT CDCL3
EXREF 0.00 ppm
BF 0.25 Hz
RGAIN 645
TEN040621
PPM
F
Me
OH
O
Cl

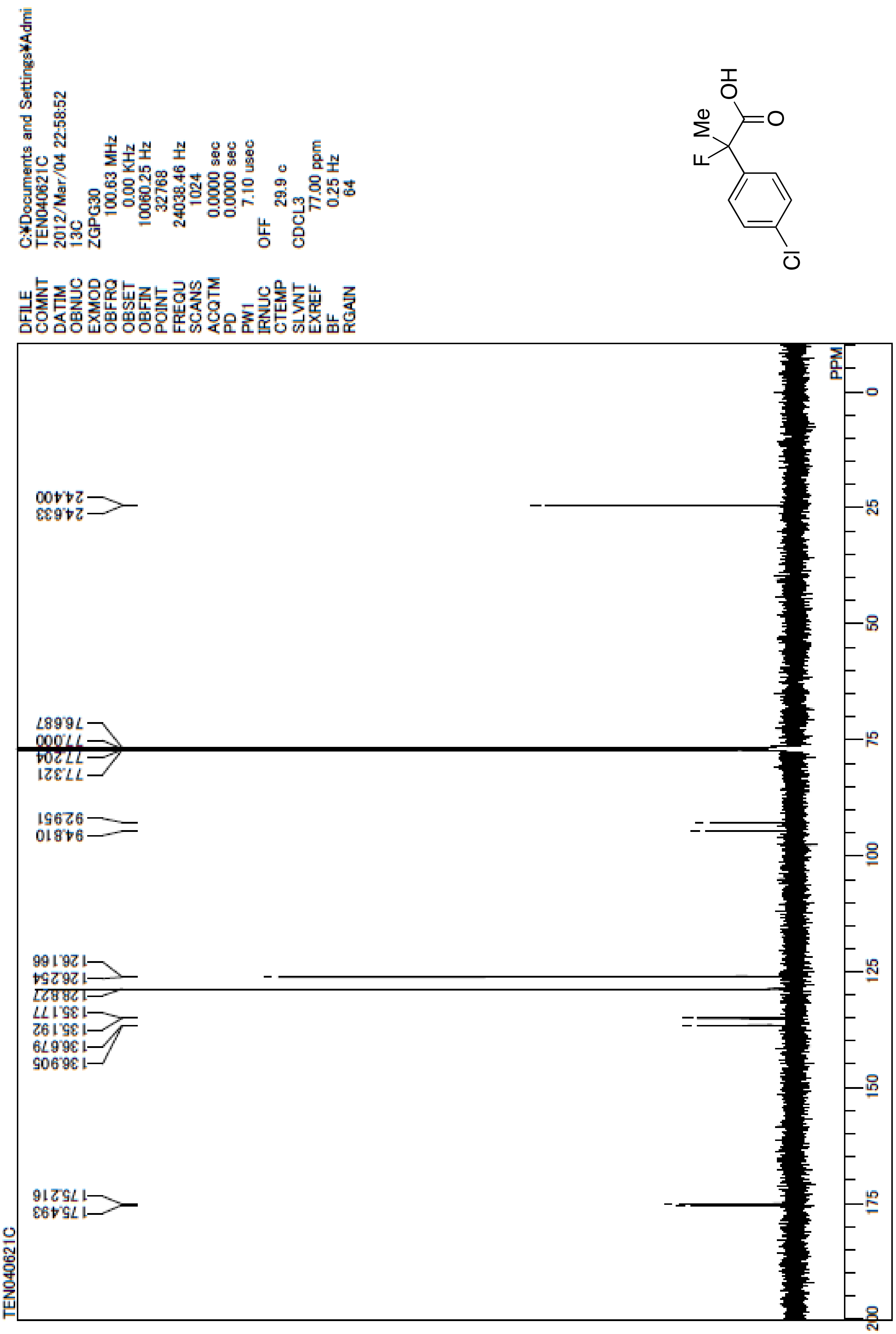
DFILE C:¥Documents and Settings¥Admi
COMNT TEN040621C
DATIM 2012/Mar/04 22:58:52
OBNUC 13C
EXMOD ZGPG30
OBFRQ 100.63 MHz
OBSET 0.00 KHz
OBFIN 10060.25 Hz
POINT 32768
FREQU 24038.46 Hz
SCANS 1024
ACQTM 0.0000 sec
PD 0.0000 sec
PW1 7.10 usec
IRNUC OFF
CTEMP 29.9 c
SLVNT CDCL3
EXREF 77.00 ppm
BF 0.25 Hz
RGAIN 64
F
Me
OH
O
Cl
24.400
24.633
76.687
77.000
77.204
77.321
92.951
94.810
126.166
126.254
128.827
135.177
135.192
136.679
136.905
175.216
175.493
TEN040621C
PPM
0
25
50
75
100
125
150
175
200

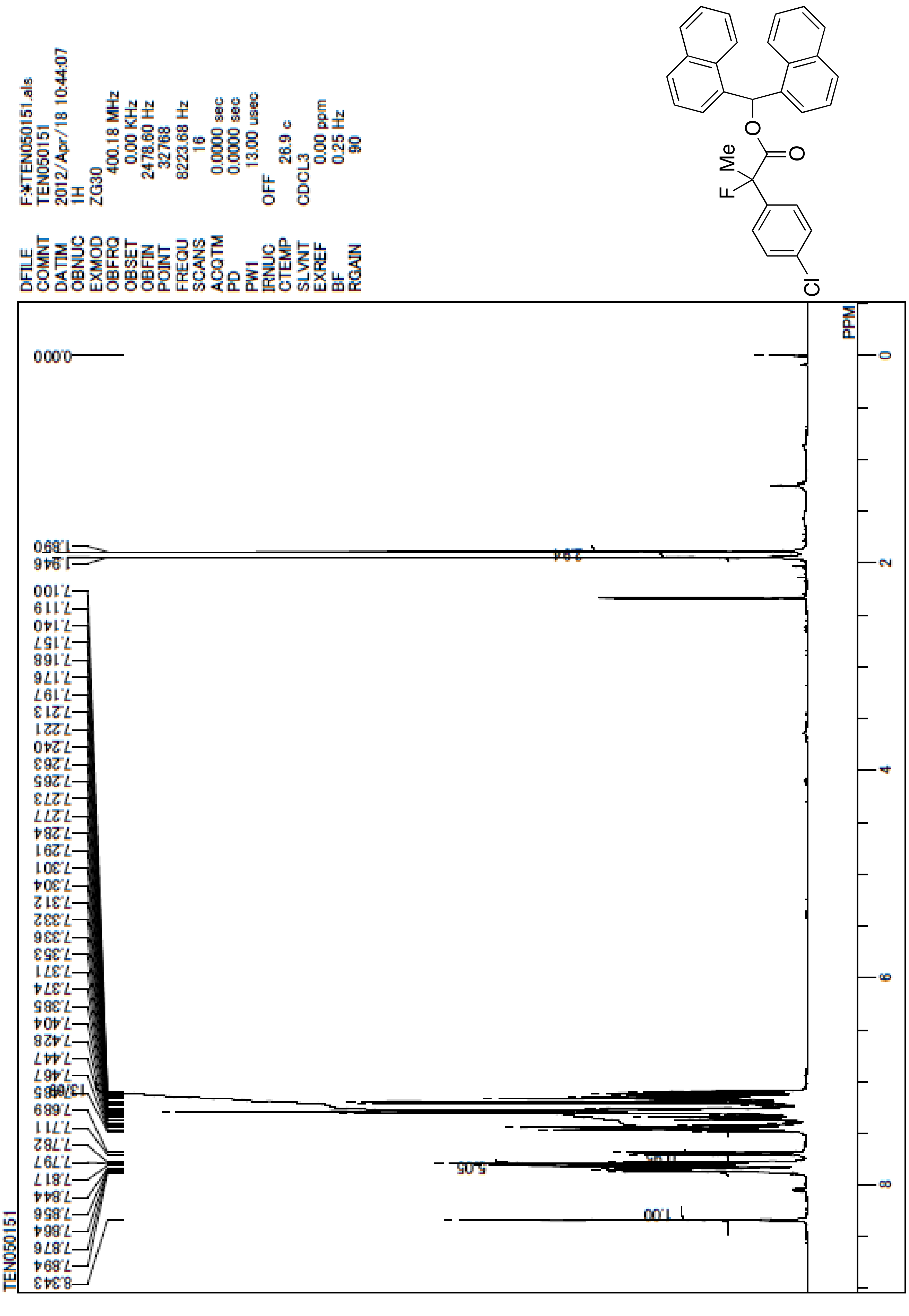
DFILE F:¥TEN050151.als
COMNT TEN050151
DATIM 2012/Apr/18 10:44:07
OBNUC 1H
EXMOD ZG30
OBFRQ 400.18 MHz
OBSET 0.00 KHz
OBFIN 2478.60 Hz
POINT 32768
FREQU 8223.68 Hz
SCANS 16
ACQTM 0.0000 sec
PD 0.0000 sec
PW1 13.00 usec
IRNUC OFF
CTEMP 26.9 c
SLVNT CDCL3
EXREF 0.00 ppm
BF 0.25 Hz
RGAIN 90
TEN050151
PPM
F
Me
O
Cl

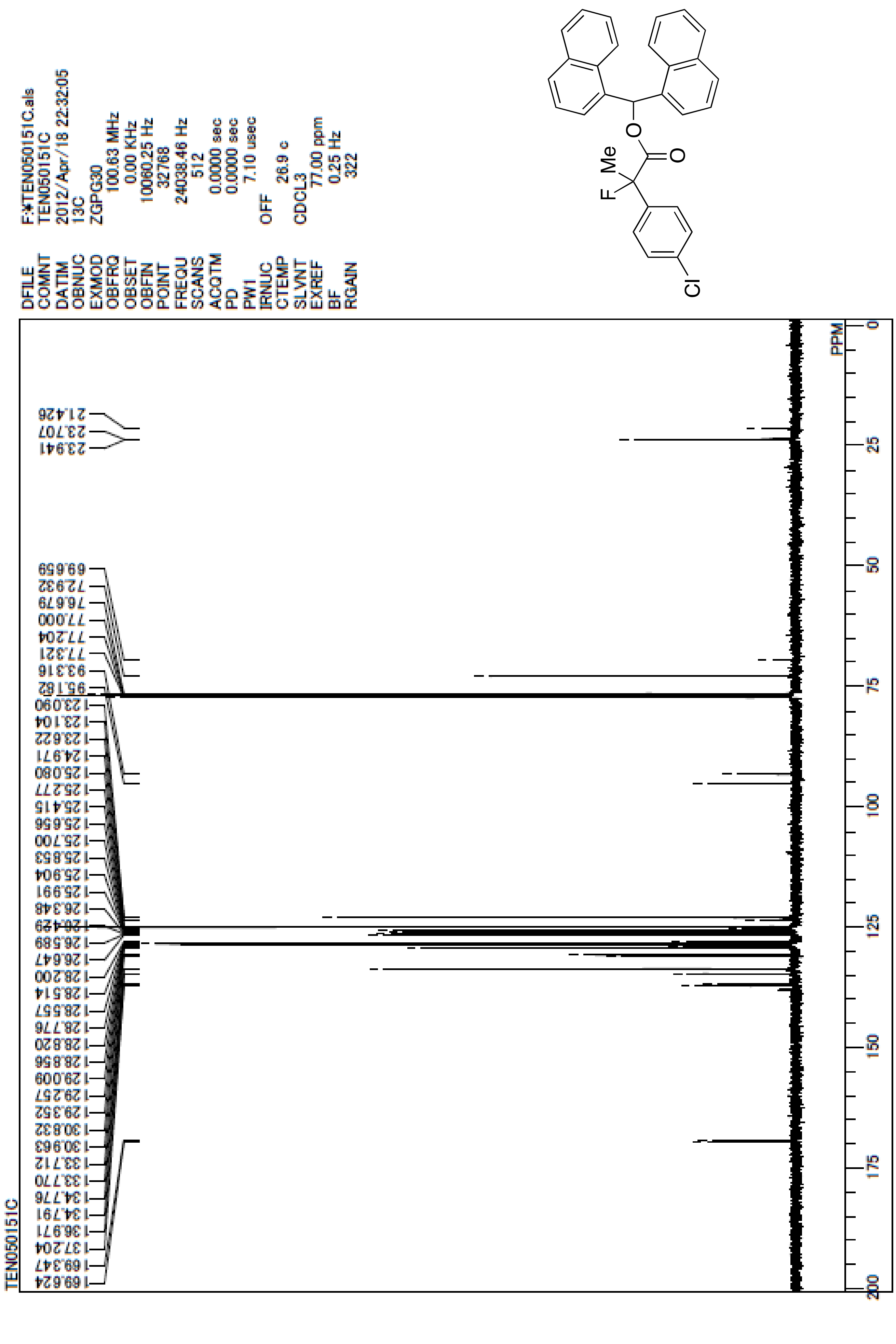
DFILE F:¥TEN050151C.als
COMNT TEN050151C
DATIM 2012/Apr/18 22:32:05
OBNUC 13C
EXMOD ZGPG30
OBFRQ 100.63 MHz
OBSET 0.00 KHz
OBFIN 10080.25 Hz
POINT 32768
FREQU 24038.46 Hz
SCANS 512
ACQTM 0.0000 sec
PD 0.0000 sec
PW1 7.10 usec
IRNUC OFF
CTEMP 26.9 c
SLVNT CDCL3
EXREF 77.00 ppm
BF 0.25 Hz
RGAIN 322
TEN050151C
21.426
23.707
23.941
69.659
72.932
76.679
77.000
77.204
77.321
93.316
95.182
123.090
123.104
123.622
124.971
125.080
125.277
125.415
125.656
125.700
125.853
125.904
125.991
126.348
126.429
126.589
126.647
128.200
128.514
128.557
128.776
128.820
128.856
129.009
129.257
129.352
130.832
130.963
133.712
133.770
134.776
134.791
136.971
137.204
169.347
169.624
PPM
0
25
50
75
100
125
150
175
200
F
Me
O
O
Cl

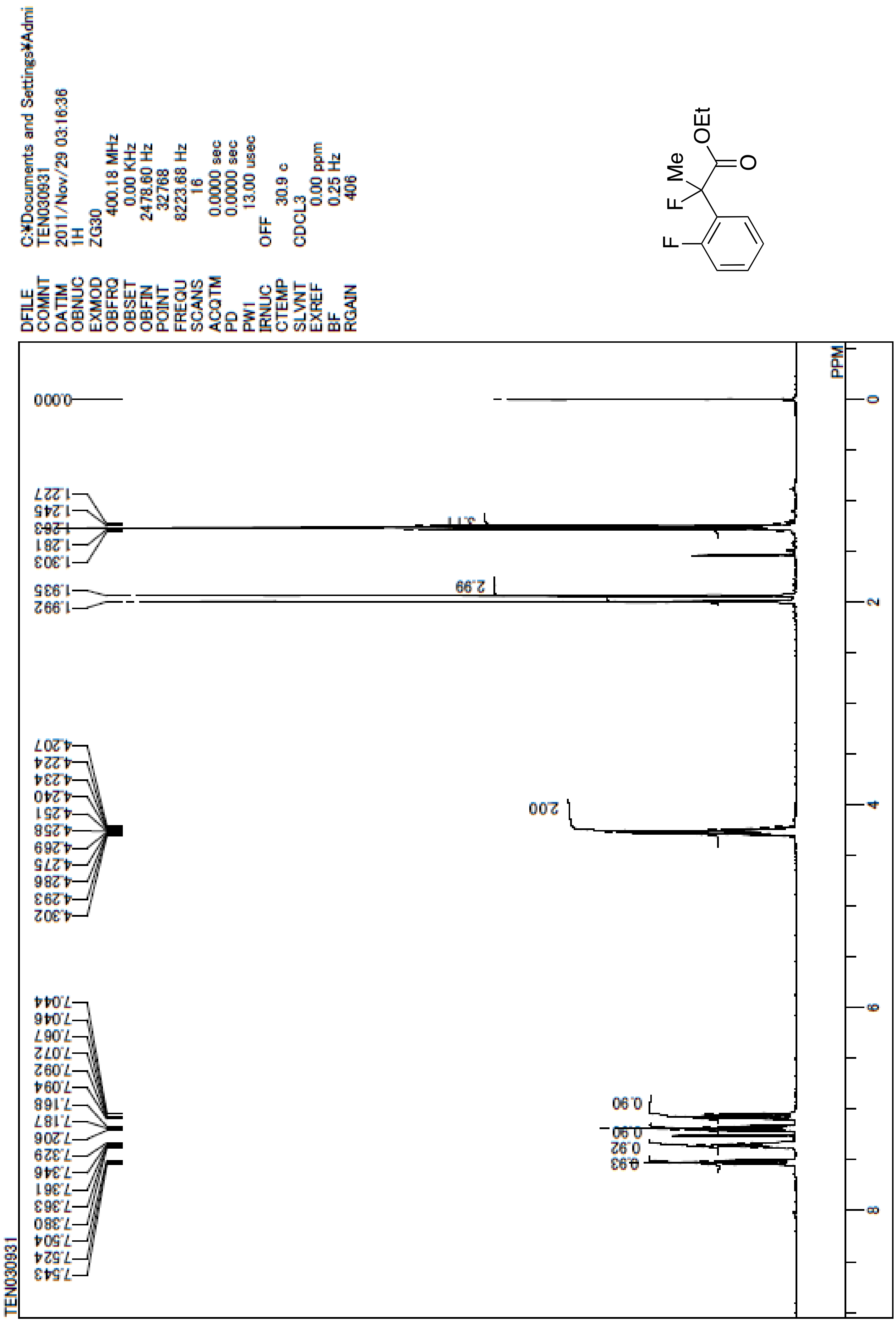
DFILE C:¥Documents and Settings¥Admi
COMNT TEN030931
DATIM 2011/Nov/29 03:16:36
OBNUC 1H
EXMOD ZG30
OBFRQ 400.18 MHz
OBSET 0.00 KHz
OBFIN 2478.60 Hz
POINT 32768
FREQU 8223.68 Hz
SCANS 16
ACQTM 0.0000 sec
PD 0.0000 sec
PW1 13.00 usec
IRNUC OFF
CTEMP 30.9 c
SLVNT CDCL3
EXREF 0.00 ppm
BF 0.25 Hz
RGAIN 406
TEN030931
PPM
F
F
Me
OEt
O

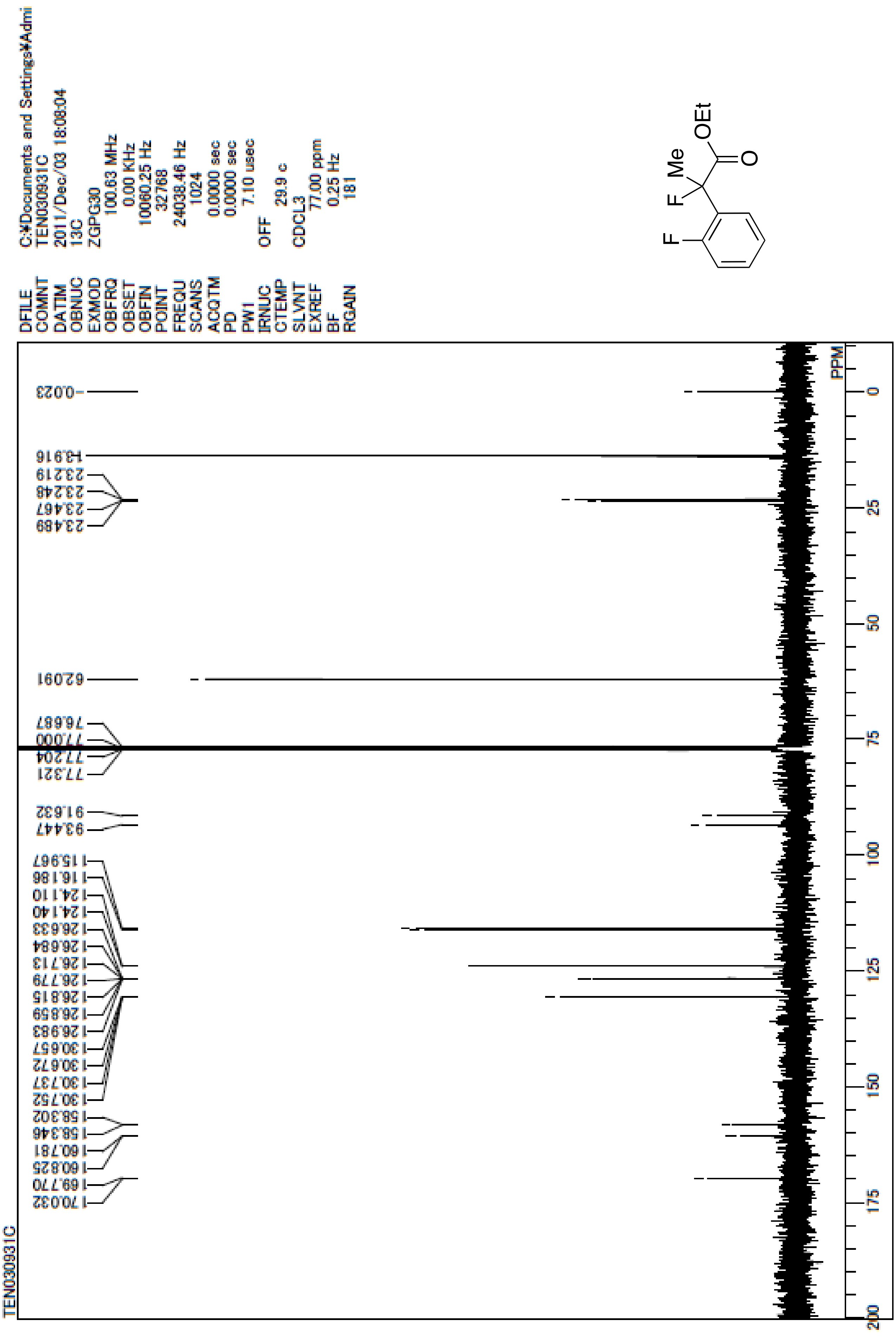
DFILE C:¥Documents and Settings¥Admi
COMNT TEN030931C
DATIM 2011/Dec/03 18:08:04
OBNUC 13C
EXMOD ZGPG30
OBFRQ 100.63 MHz
OBSET 0.00 KHz
OBFIN 10060.25 Hz
POINT 32768
FREQU 24038.46 Hz
SCANS 1024
ACQTM 0.0000 sec
PD 0.0000 sec
PW1 7.10 usec
IRNUC OFF
CTEMP 29.9 c
SLVNT CDCL3
EXREF 77.00 ppm
BF 0.25 Hz
RGAIN 181
F
F
Me
OEt
O
TEN030931C
-0.023
13.916
23.219
23.248
23.467
23.489
62.091
76.687
77.000
77.204
77.321
91.632
93.447
115.967
116.186
124.110
124.140
126.633
126.684
126.713
126.779
126.815
126.859
126.983
130.657
130.672
130.737
130.752
158.302
158.346
160.781
160.825
169.770
170.032
PPM
0
25
50
75
100
125
150
175
200

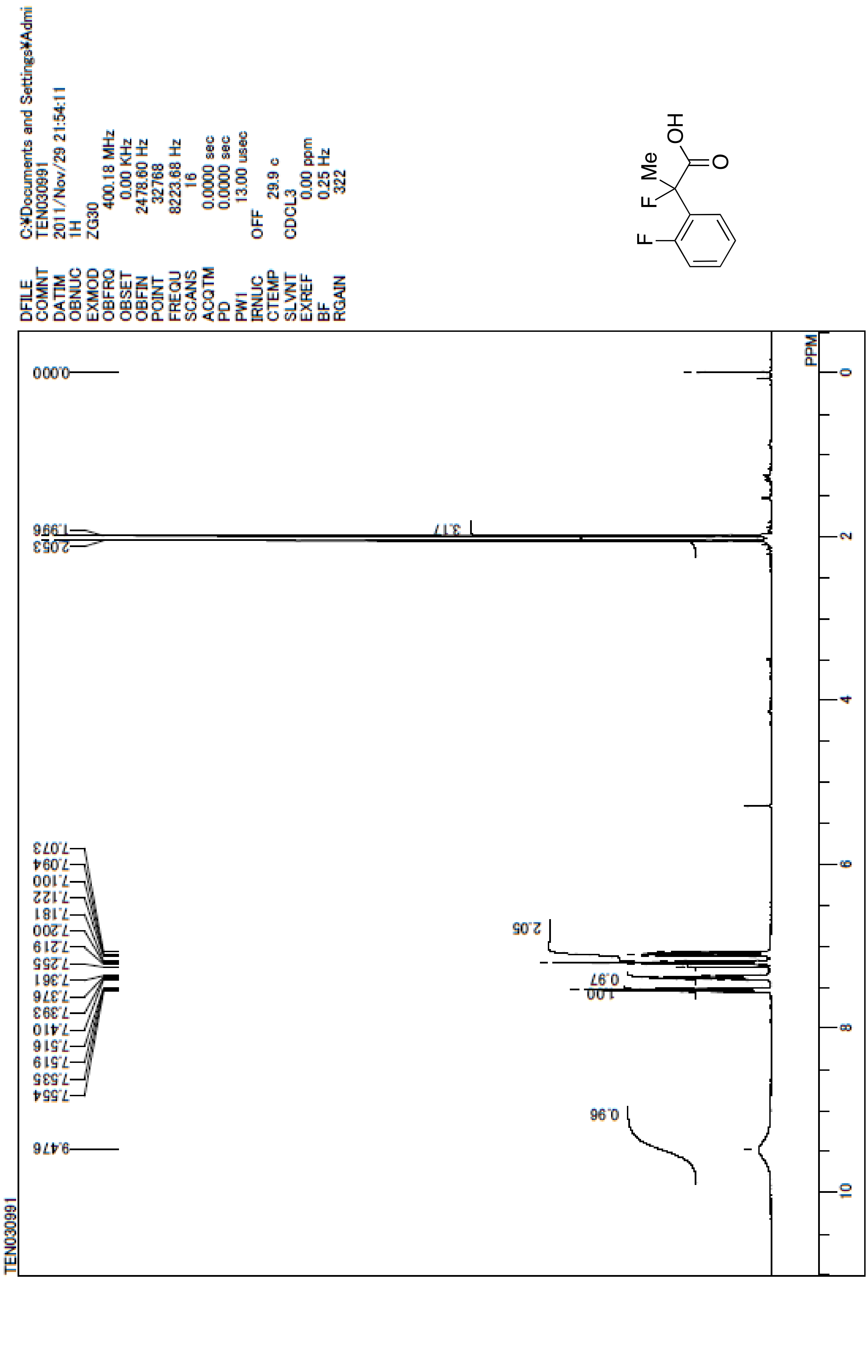
DFILE C:¥Documents and Settings¥Admi
COMNT TEN030991
DATIM 2011/Nov/29 21:54:11
OBNUC 1H
EXMOD ZG30
OBFRQ 400.18 MHz
OBSET 0.00 KHz
OBFIN 2478.60 Hz
POINT 32768
FREQU 8223.68 Hz
SCANS 16
ACQTM 0.0000 sec
PD 0.0000 sec
PW1 13.00 usec
IRNUC OFF
CTEMP 29.9 c
SLVNT CDCL3
EXREF 0.00 ppm
BF 0.25 Hz
RGAIN 322
F
F
Me
OH
O
TEN030991
PPM

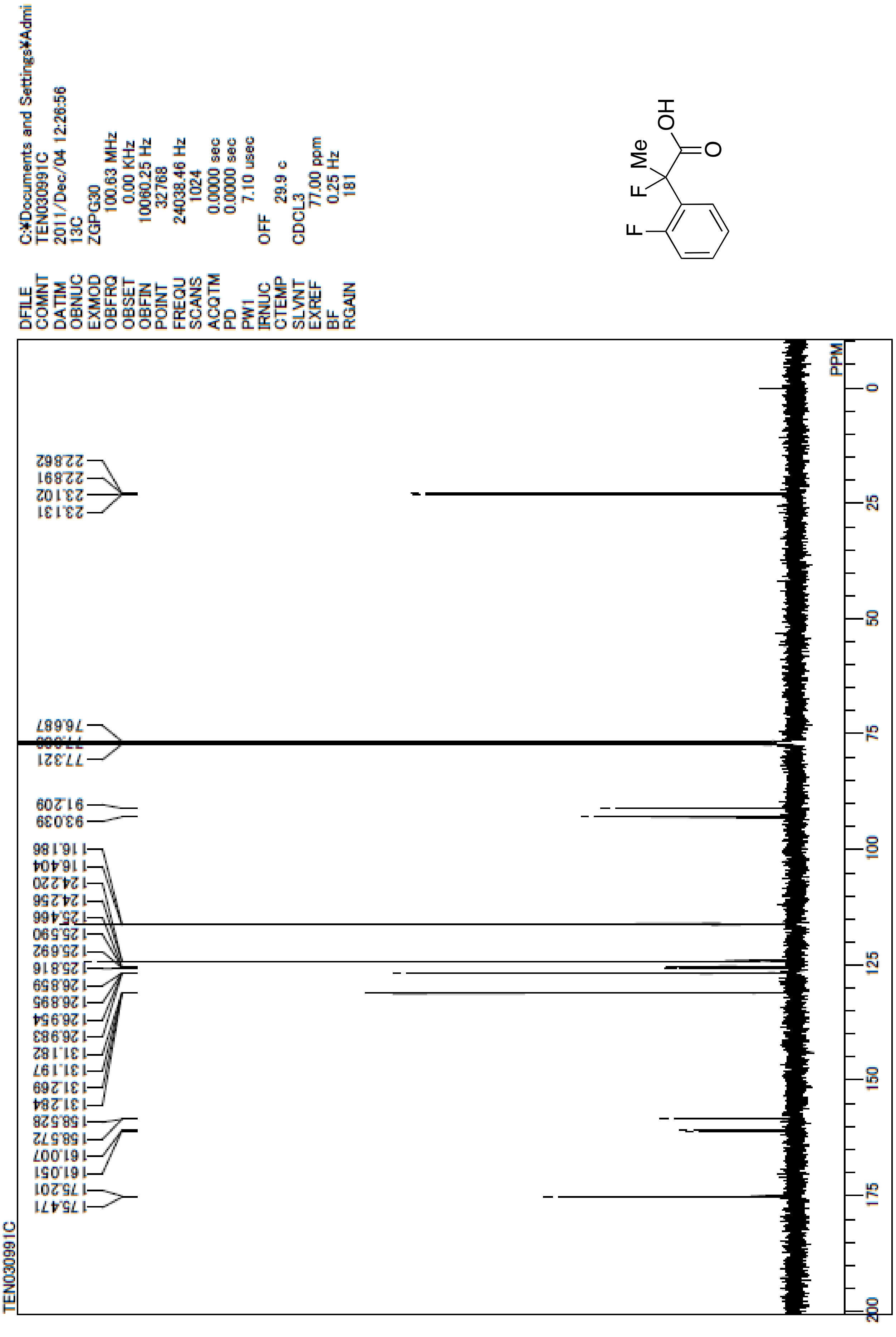
TEN030991C
DFILE C:¥Documents and Settings¥Admi
COMNT TEN030991C
DATIM 2011/Dec/04 12:26:56
OBNUC 13C
EXMOD ZGPG30
OBFRQ 100.63 MHz
OBSET 0.00 KHz
OBFIN 10060.25 Hz
POINT 32768
FREQU 24038.46 Hz
SCANS 1024
ACQTM 0.0000 sec
PD 0.0000 sec
PW1 7.10 usec
IRNUC OFF
CTEMP 29.9 c
SLVNT CDCL3
EXREF 77.00 ppm
BF 0.25 Hz
RGAIN 181
22.862
22.891
23.102
23.131
76.687
77.321
91.209
93.039
116.186
116.404
124.220
124.256
125.466
125.590
125.692
125.816
126.859
126.895
126.954
126.983
131.182
131.197
131.269
131.284
158.528
158.572
161.007
161.051
175.201
175.471
PPM
0
25
50
75
100
125
150
175
200
F
F
Me
OH
O

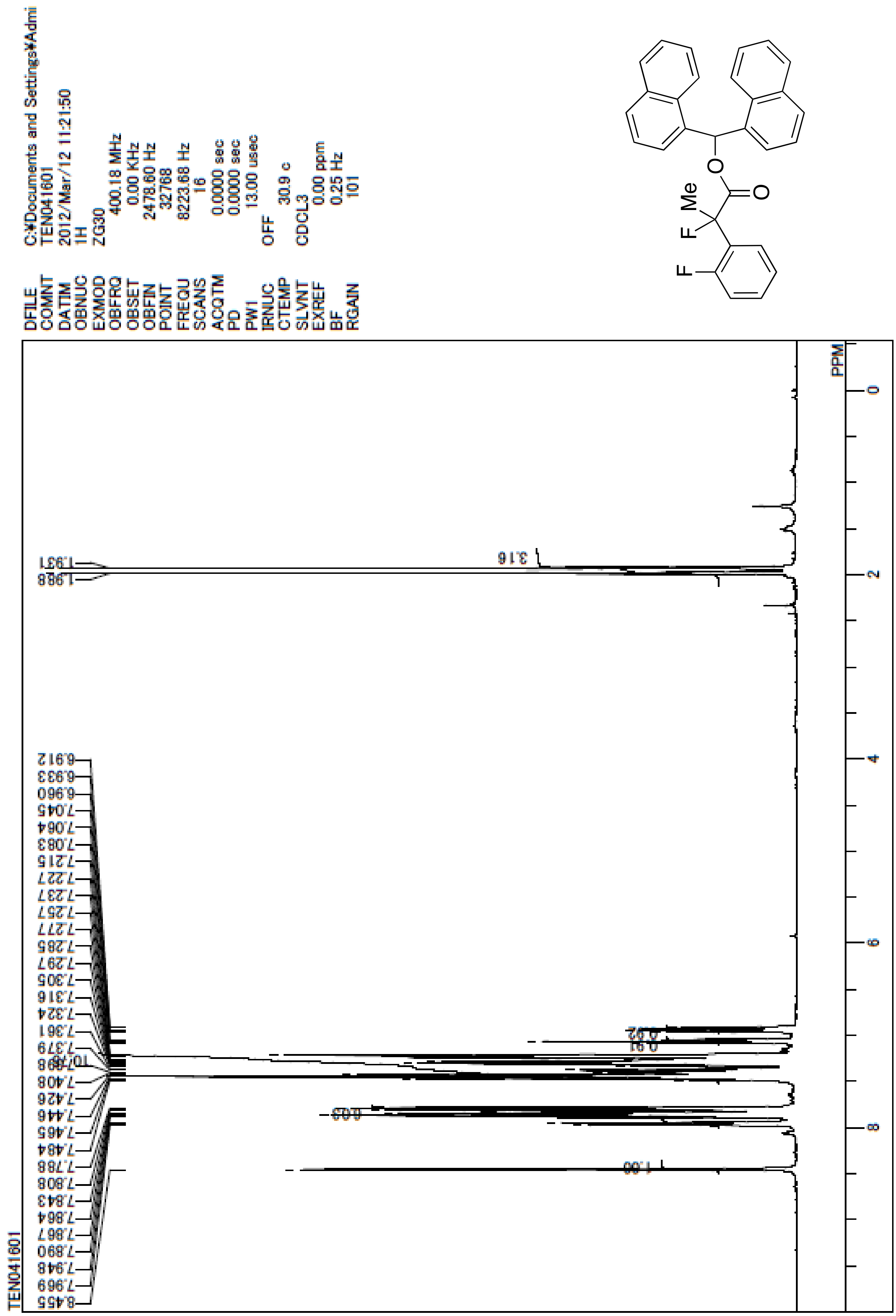
DFILE C:¥Documents and Settings¥Admi
COMNT TEN041601
DATIM 2012/Mar/12 11:21:50
OBNUC 1H
EXMOD ZG30
OBFRQ 400.18 MHz
OBSET 0.00 KHz
OBFIN 2478.60 Hz
POINT 32768
FREQU 8223.68 Hz
SCANS 16
ACQTM 0.0000 sec
PD 0.0000 sec
PW1 13.00 usec
IRNUC OFF
CTEMP 30.9 c
SLVNT CDCL3
EXREF 0.00 ppm
BF 0.25 Hz
RGAIN 101
F
F
Me
O
O
TEN041601
PPM

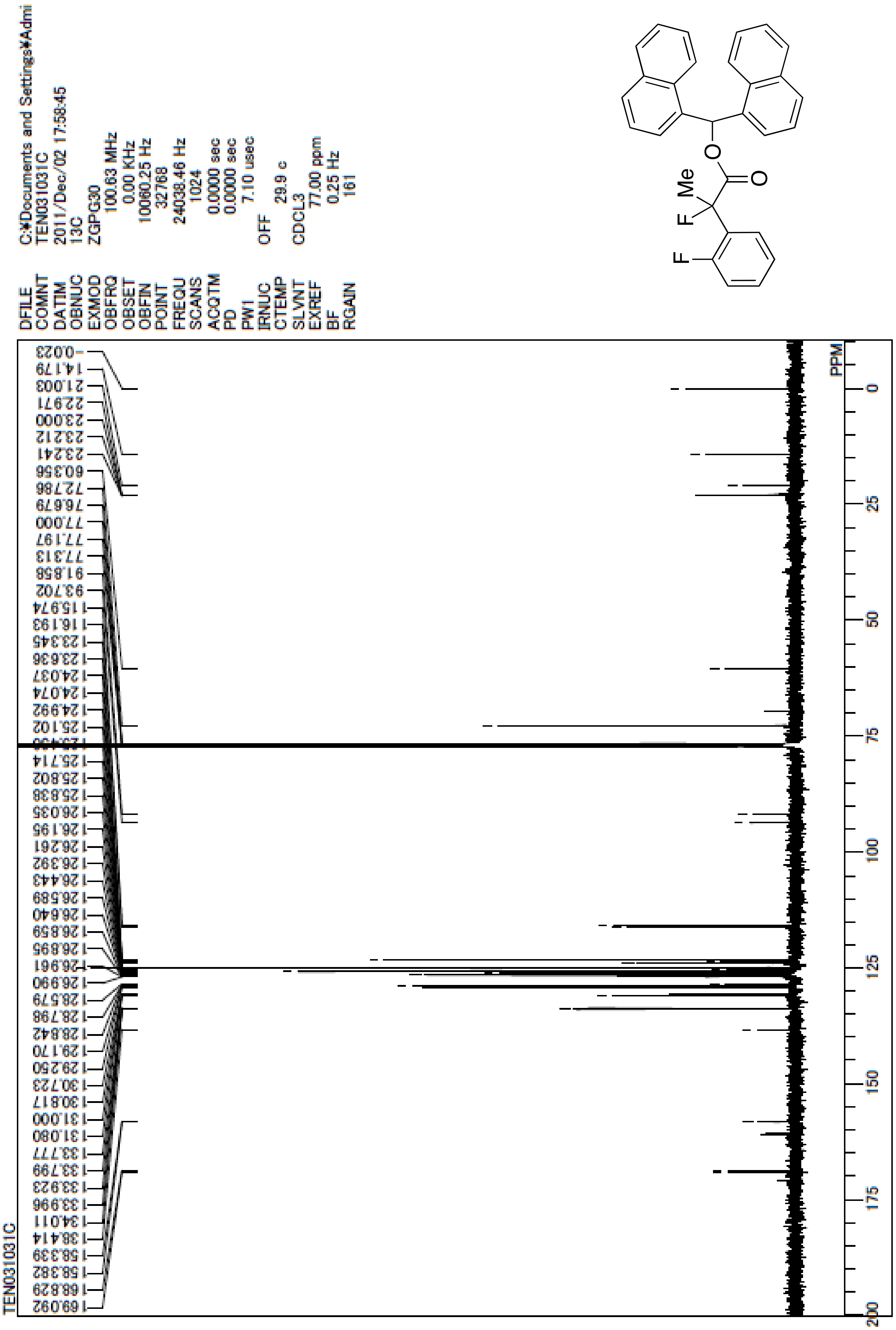
DFILE C:¥Documents and Settings¥Admi
COMNT TEN031031C
DATIM 2011/Dec/02 17:58:45
OBNUC 13C
EXMOD ZGPG30
OBFRQ 100.63 MHz
OBSET 0.00 KHz
OBFIN 10060.25 Hz
POINT 32768
FREQU 24038.46 Hz
SCANS 1024
ACQTM 0.0000 sec
PD 0.0000 sec
PW1 7.10 usec
IRNUC OFF
CTEMP 29.9 c
SLVNT CDCL3
EXREF 77.00 ppm
BF 0.25 Hz
RGAIN 161
F
F
Me
O
O
TEN031031C
-0.023
14.179
21.003
22.971
23.000
23.212
23.241
60.356
72.786
76.679
77.000
77.197
77.313
91.858
93.702
115.974
116.193
123.345
123.636
124.037
124.074
124.992
125.102
125.714
125.802
125.838
126.035
126.195
126.261
126.392
126.443
126.589
126.640
126.859
126.895
126.961
126.990
128.579
128.798
128.842
129.170
129.250
130.723
130.817
131.000
131.080
133.777
133.799
133.923
133.996
134.011
138.414
158.339
158.382
168.829
169.092
PPM
0
25
50
75
100
125
150
175
200

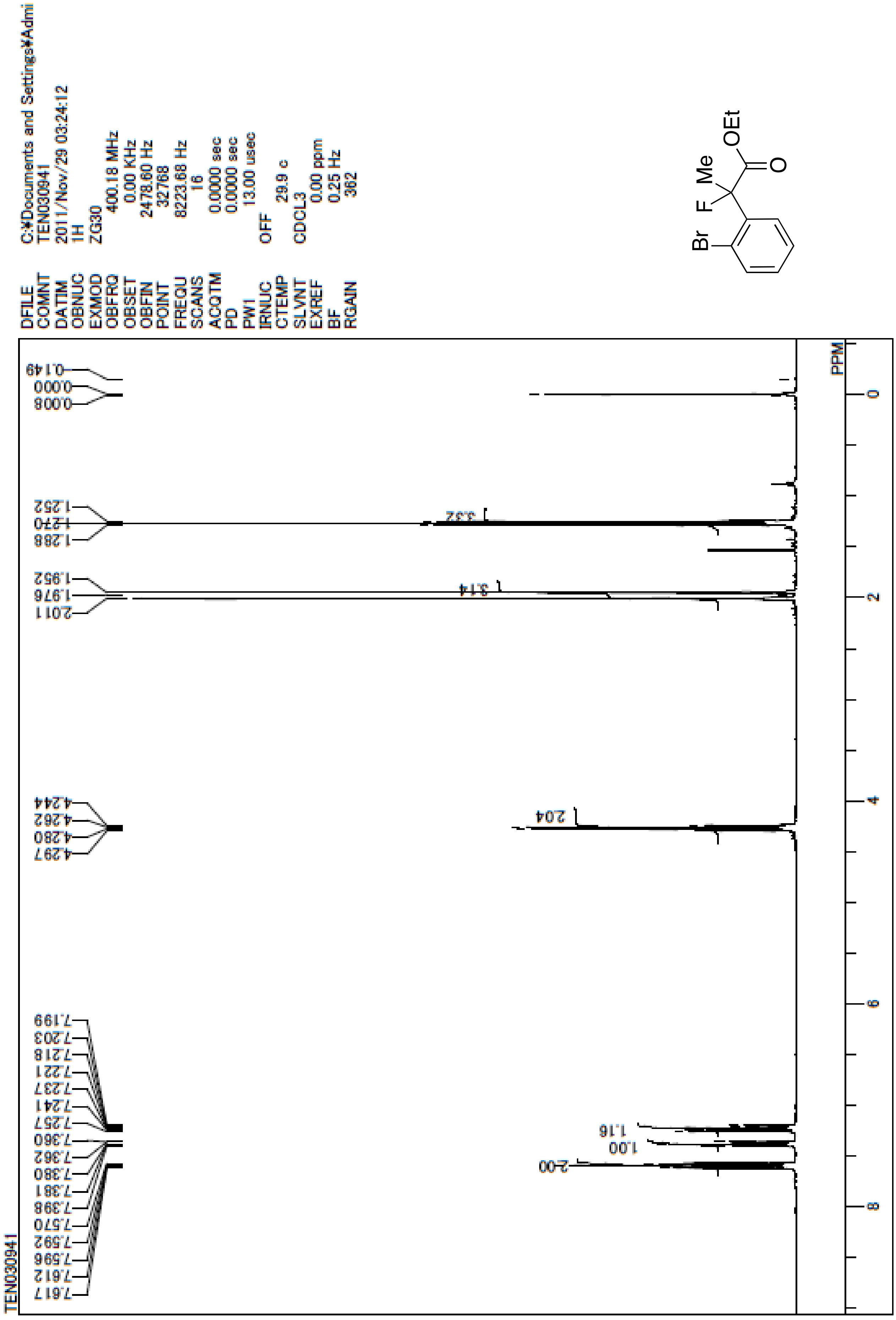

DFILE C:¥Documents and Settings¥Admi
COMNT TEN030941
DATIM 2011/Nov/29 03:24:12
OBNUC 1H
EXMOD ZG30
OBFRQ 400.18 MHz
OBSET 0.00 KHz
OBFIN 2478.60 Hz
POINT 32768
FREQU 8223.68 Hz
SCANS 16
ACQTM 0.0000 sec
PD 0.0000 sec
PW1 13.00 usec
IRNUC OFF
CTEMP 29.9 c
SLVNT CDCL3
EXREF 0.00 ppm
BF 0.25 Hz
RGAIN 362
Br
F
Me
OEt
O
TEN030941
PPM

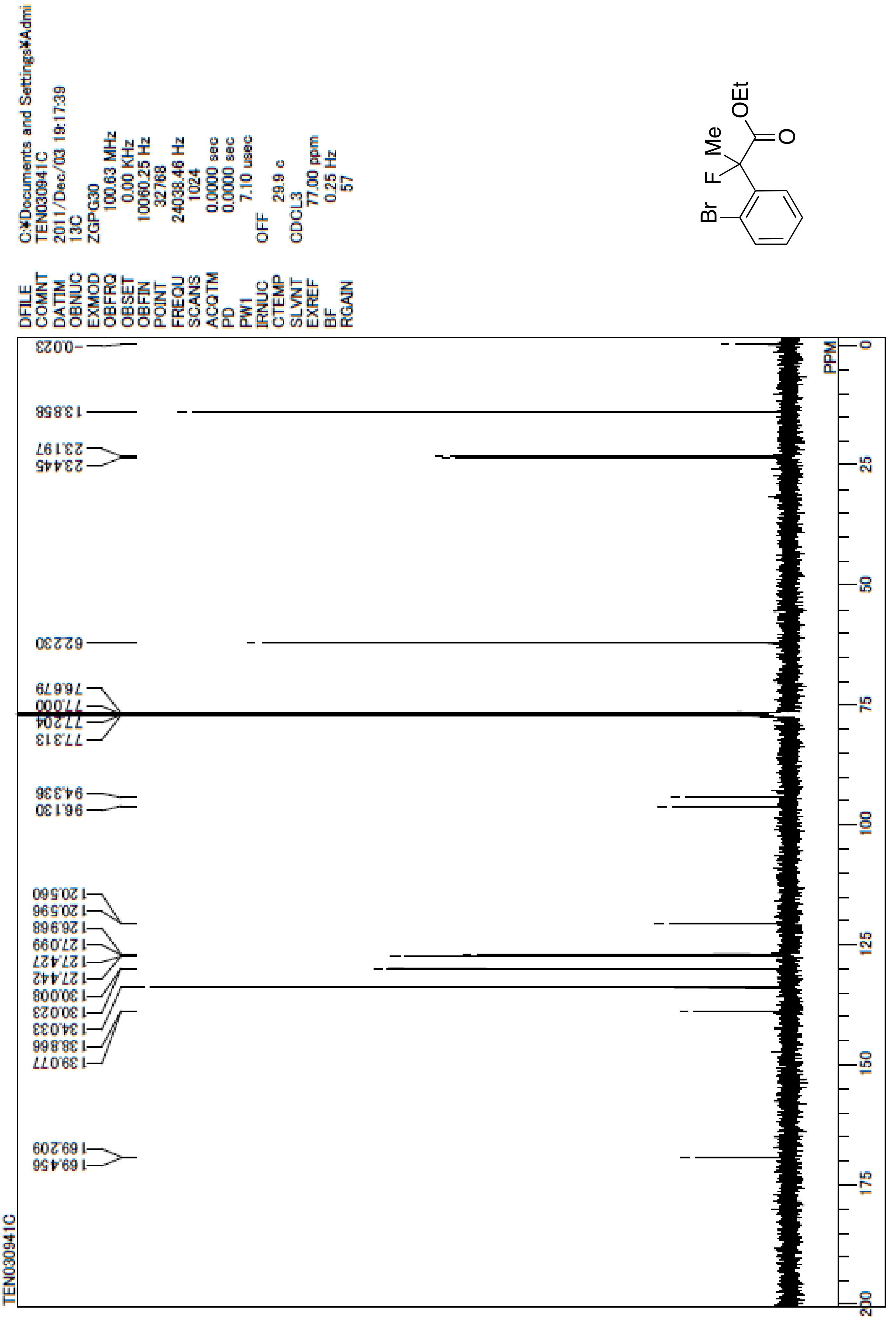
DFILE C:¥Documents and Settings¥Admi
COMNT TEN030941C
DATIM 2011/Dec/03 19:17:39
OBNUC 13C
EXMOD ZGPG30
OBFRQ 100.63 MHz
OBSET 0.00 KHz
OBFIN 10060.25 Hz
POINT 32768
FREQU 24038.46 Hz
SCANS 1024
ACQTM 0.0000 sec
PD 0.0000 sec
PW1 7.10 usec
IRNUC OFF
CTEMP 29.9 c
SLVNT CDCL3
EXREF 77.00 ppm
BF 0.25 Hz
RGAIN 57
TEN030941C
-0.023
13.858
23.197
23.445
62.230
76.679
77.000
77.204
77.313
94.336
96.130
120.560
120.596
126.968
127.099
127.427
127.442
130.008
130.023
134.033
138.866
139.077
169.209
169.456
PPM
0
25
50
75
100
125
150
175
200
Br
F
Me
OEt
O

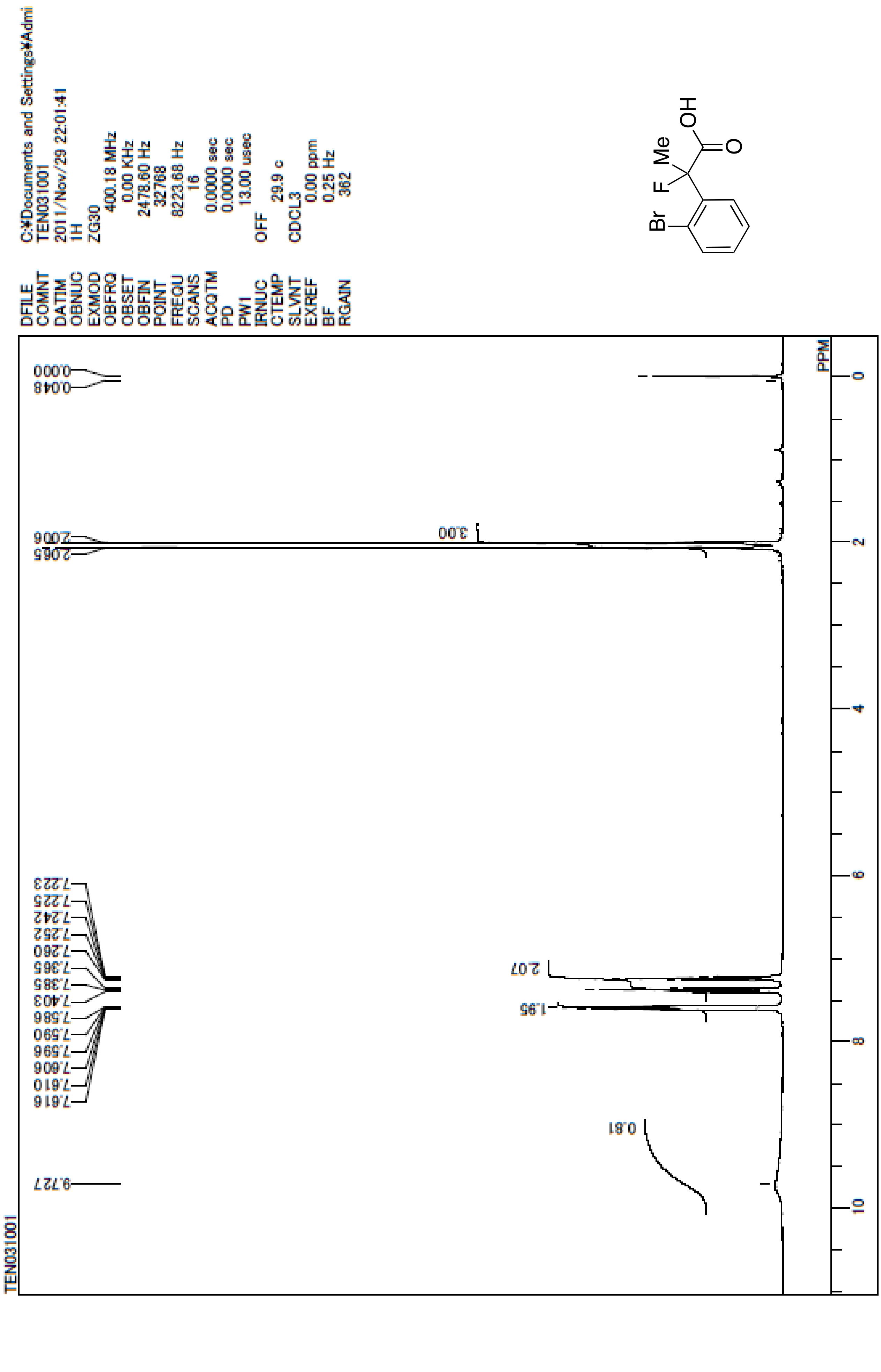

DFILE C:¥Documents and Settings¥Admi
COMNT TEN031001
DATIM 2011/Nov/29 22:01:41
OBNUC 1H
EXMOD ZG30
OBFRQ 400.18 MHz
OBSET 0.00 KHz
OBFIN 2478.60 Hz
POINT 32768
FREQU 8223.68 Hz
SCANS 16
ACQTM 0.0000 sec
PD 0.0000 sec
PW1 13.00 usec
IRNUC OFF
CTEMP 29.9 c
SLVNT CDCL3
EXREF 0.00 ppm
BF 0.25 Hz
RGAIN 362
Br
F
Me
OH
O
TEN031001
PPM
0.000
0.048
2.006
2.065
3.00
7.223
7.225
7.242
7.252
7.260
7.365
7.385
7.403
7.586
7.590
7.596
7.606
7.610
7.616
2.07
1.95
0.81
9.727
0
2
4
6
8
10

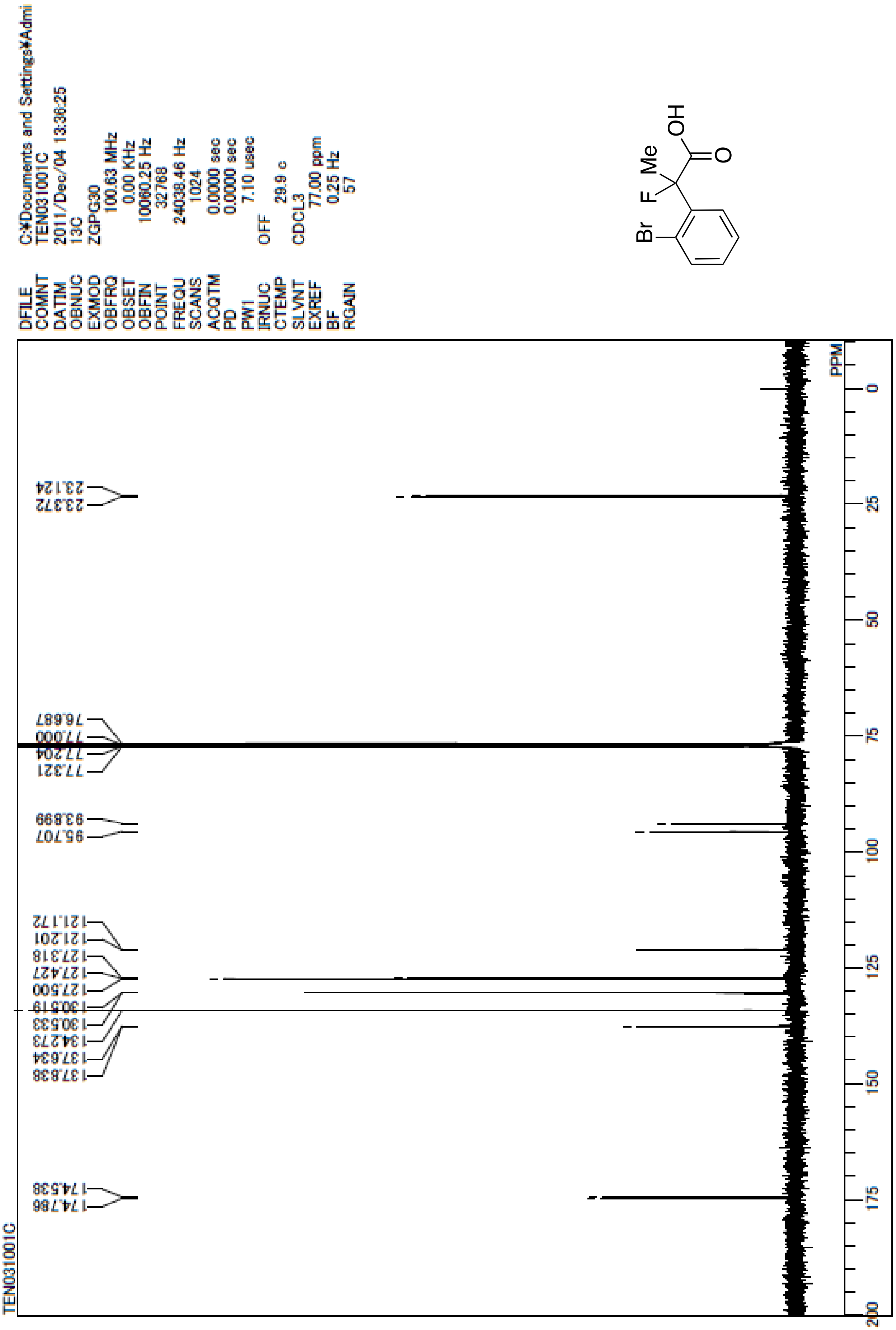
DFILE C:¥Documents and Settings¥Admi
COMNT TEN031001C
DATIM 2011/Dec/04 13:36:25
OBNUC 13C
EXMOD ZGPG30
OBFRQ 100.63 MHz
OBSET 0.00 KHz
OBFIN 10060.25 Hz
POINT 32768
FREQU 24038.46 Hz
SCANS 1024
ACQTM 0.0000 sec
PD 0.0000 sec
PW1 7.10 usec
IRNUC OFF
CTEMP 29.9 c
SLVNT CDCL3
EXREF 77.00 ppm
BF 0.25 Hz
RGAIN 57
Br
F
Me
OH
O
TEN031001C
23.124
23.372
76.687
77.000
77.204
77.321
93.899
95.707
121.172
121.201
127.318
127.427
127.500
130.519
130.533
134.273
137.634
137.838
174.538
174.786
PPM
0
25
50
75
100
125
150
175
200

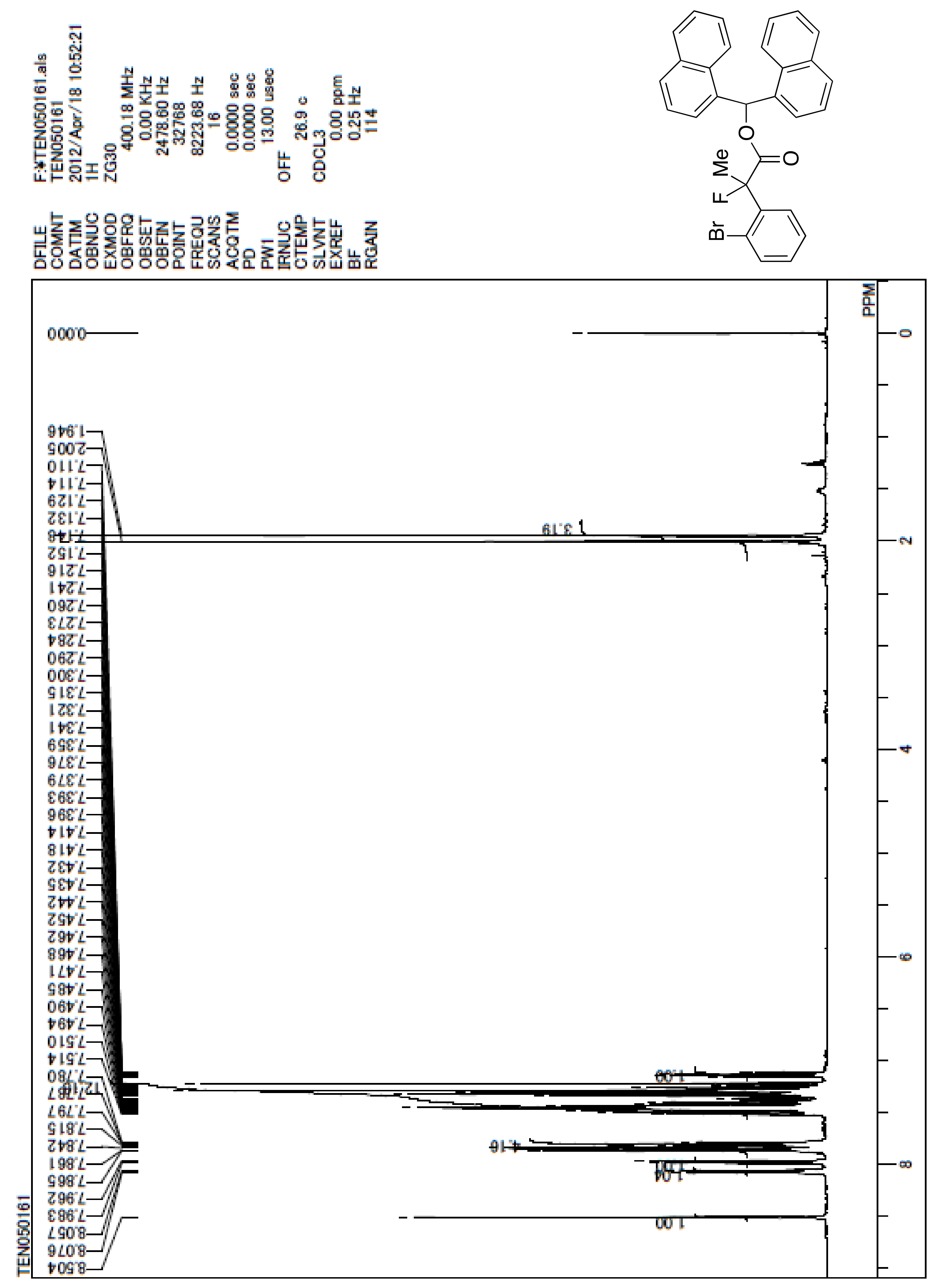
DFILE F:¥TEN050161.als
COMNT TEN050161
DATIM 2012/Apr/18 10:52:21
OBNUC 1H
EXMOD ZG30
OBFRQ 400.18 MHz
OBSET 0.00 KHz
OBFIN 2478.60 Hz
POINT 32768
FREQU 8223.68 Hz
SCANS 16
ACQTM 0.0000 sec
PD 0.0000 sec
PW1 13.00 usec
IRNUC OFF
CTEMP 26.9 c
SLVNT CDCL3
EXREF 0.00 ppm
BF 0.25 Hz
RGAIN 114
TEN050161
PPM
Br
F
Me
O
O

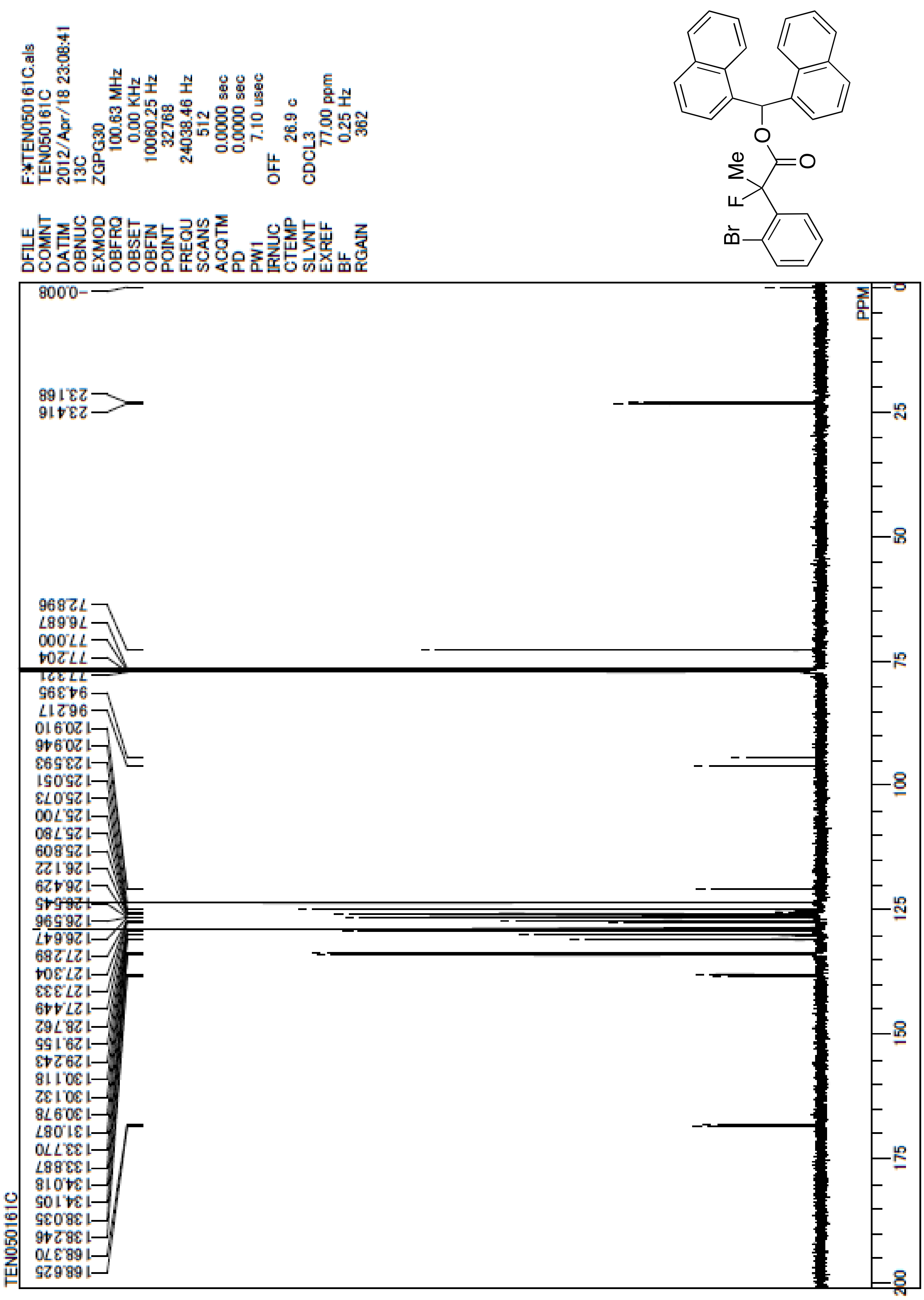
TEN050161C
DFILE F:¥TEN050161C.als
COMNT TEN050161C
DATIM 2012/Apr/18 23:08:41
OBNUC 13C
EXMOD ZGPG30
OBFRQ 100.63 MHz
OBSET 0.00 KHz
OBFIN 10060.25 Hz
POINT 32768
FREQU 24038.46 Hz
SCANS 512
ACQTM 0.0000 sec
PD 0.0000 sec
PW1 7.10 usec
IRNUC OFF
CTEMP 26.9 c
SLVNT CDCL3
EXREF 77.00 ppm
BF 0.25 Hz
RGAIN 362
-0.008
23.168
23.416
72.896
76.687
77.000
77.204
77.321
94.395
96.217
120.910
120.946
123.593
125.051
125.073
125.700
125.780
125.809
126.122
126.429
126.545
126.596
126.647
127.289
127.304
127.333
127.449
128.762
129.155
129.243
130.118
130.132
130.978
131.087
133.770
133.887
134.018
134.105
138.035
138.246
168.370
168.625
PPM
0
25
50
75
100
125
150
175
200
Br
F
Me
O
O

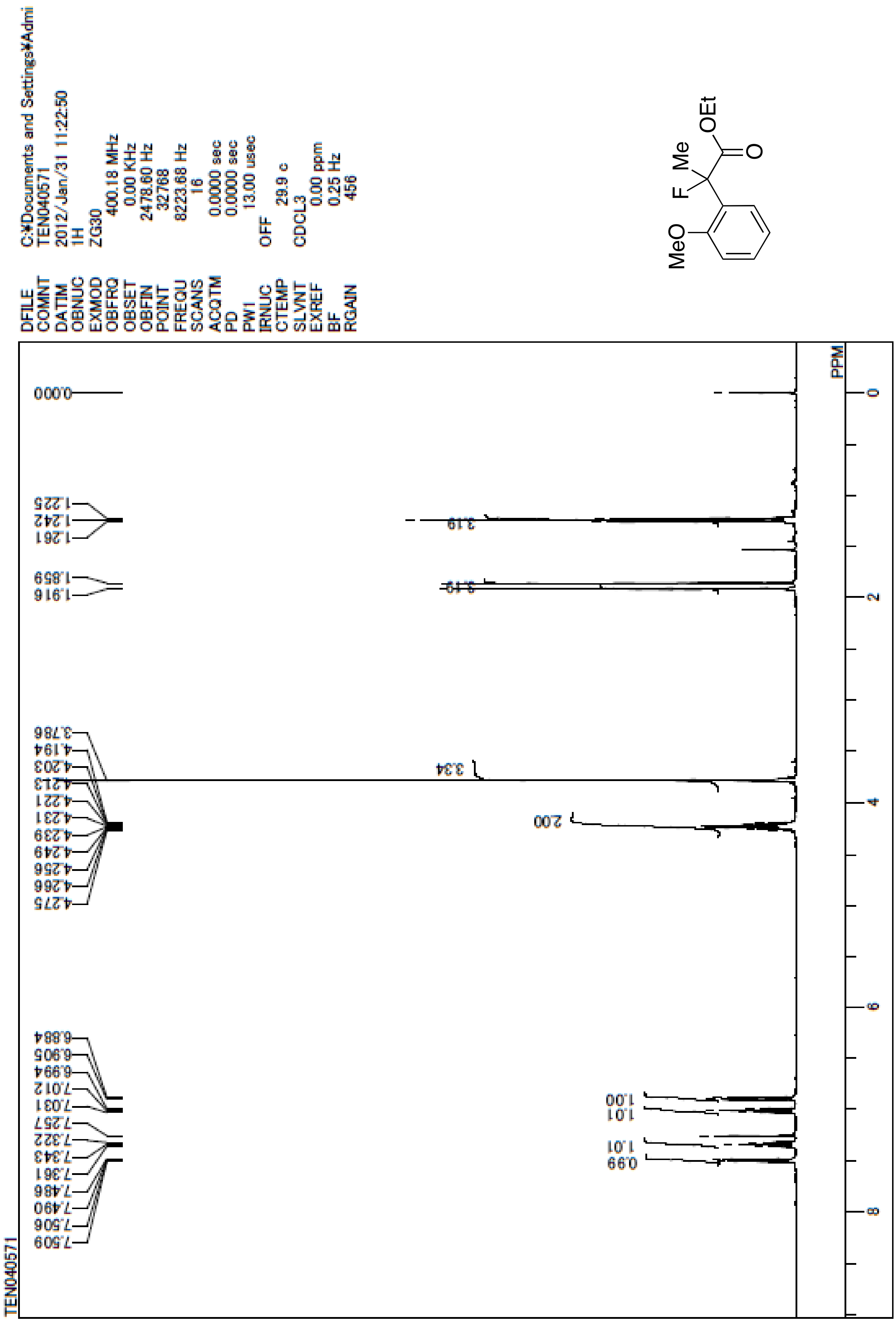
DFILE C:¥Documents and Settings¥Admi
COMNT TEN040571
DATIM 2012/Jan/31 11:22:50
OBNUC 1H
EXMOD ZG30
OBFRQ 400.18 MHz
OBSET 0.00 KHz
OBFIN 2478.60 Hz
POINT 32768
FREQU 8223.68 Hz
SCANS 16
ACQTM 0.0000 sec
PD 0.0000 sec
PW1 13.00 usec
IRNUC OFF
CTEMP 29.9 c
SLVNT CDCL3
EXREF 0.00 ppm
BF 0.25 Hz
RGAIN 456
MeO
F
Me
OEt
O
TEN040571
0.000
1.225
1.242
1.261
1.859
1.916
3.786
4.194
4.203
4.213
4.221
4.231
4.239
4.249
4.256
4.266
4.275
6.884
6.905
6.994
7.012
7.031
7.257
7.322
7.343
7.361
7.486
7.490
7.506
7.509
3.19
3.19
3.34
2.00
1.00
1.01
1.01
0.99
PPM
0
2
4
6
8

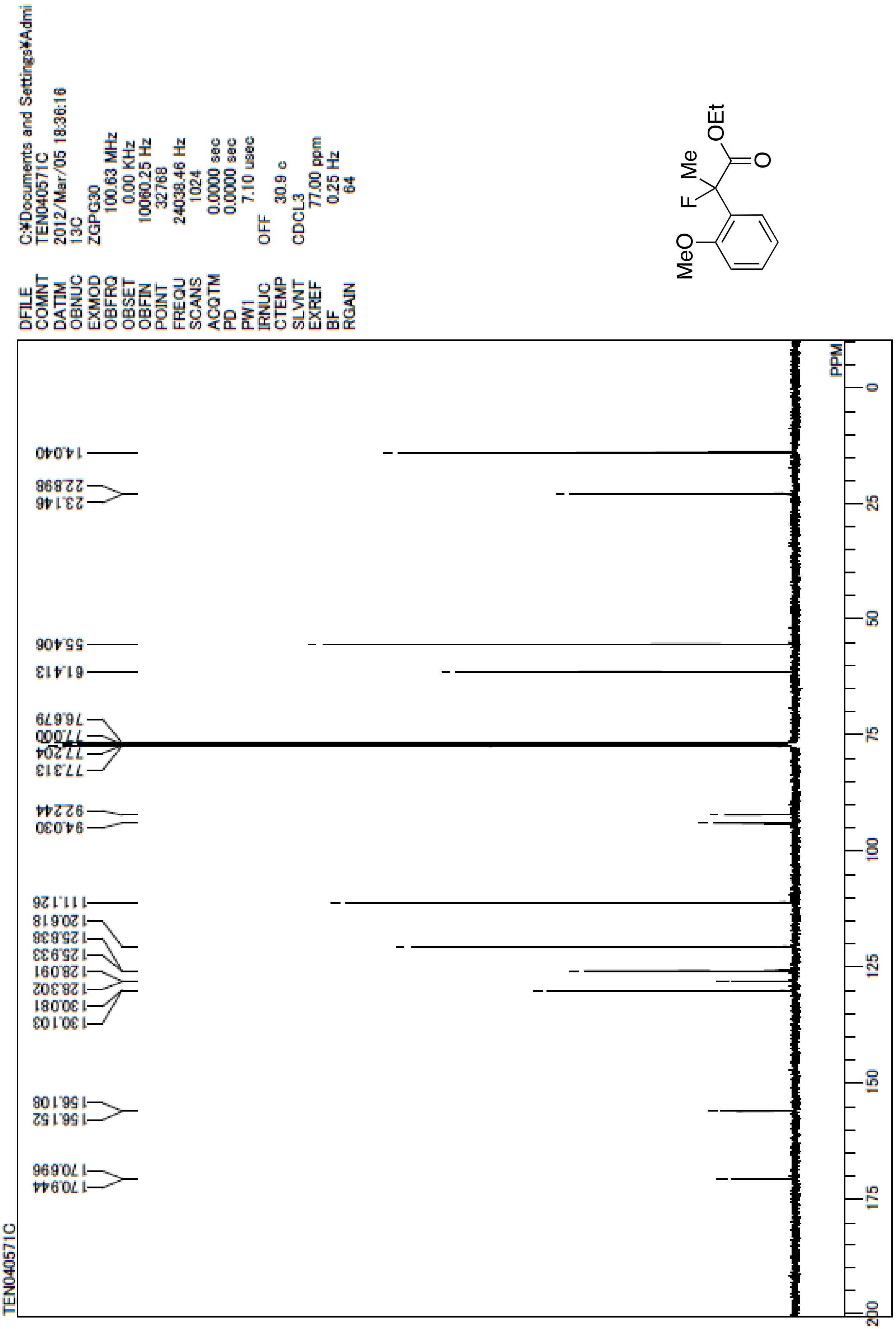
DFILE C:¥Documents and Settings¥Admi
COMNT TEN040571C
DATIM 2012/Mar/05 18:36:16
OBNUC 13C
EXMOD ZGPG30
OBFRQ 100.63 MHz
OBSET 0.00 KHz
OBFIN 10060.25 Hz
POINT 32768
FREQU 24038.46 Hz
SCANS 1024
ACQTM 0.0000 sec
PD 0.0000 sec
PW1 7.10 usec
IRNUC OFF
CTEMP 30.9 c
SLVNT CDCL3
EXREF 77.00 ppm
BF 0.25 Hz
RGAIN 64
MeO
F
Me
OEt
O
TEN040571C
PPM

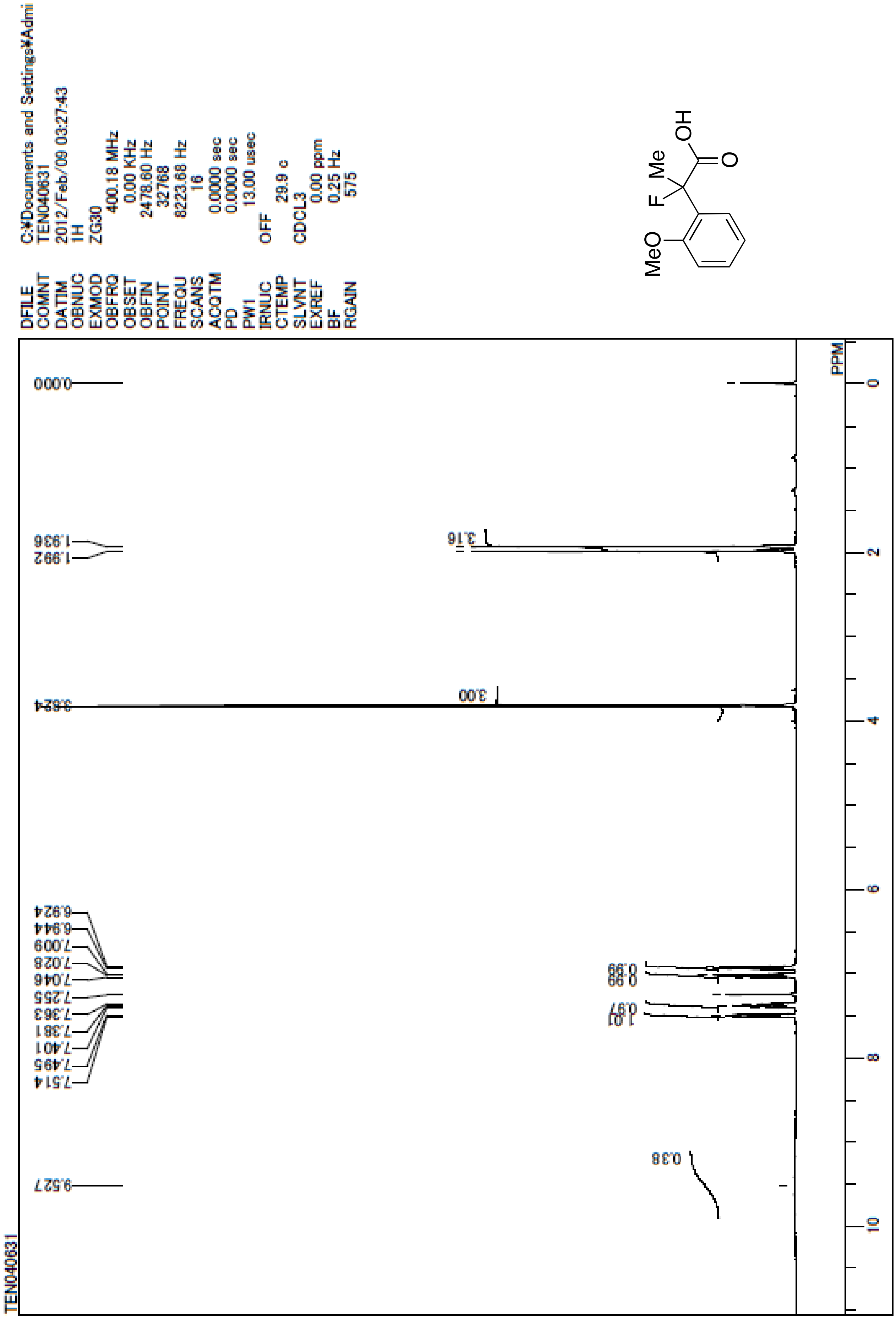
DFILE C:¥Documents and Settings¥Admi
COMNT TEN040631
DATIM 2012/Feb/09 03:27:43
OBNUC 1H
EXMOD ZG30
OBFRQ 400.18 MHz
OBSET 0.00 KHz
OBFIN 2478.60 Hz
POINT 32768
FREQU 8223.68 Hz
SCANS 16
ACQTM 0.0000 sec
PD 0.0000 sec
PW1 13.00 usec
IRNUC OFF
CTEMP 29.9 c
SLVNT CDCL3
EXREF 0.00 ppm
BF 0.25 Hz
RGAIN 575
MeO
F
Me
OH
O
TEN040631
PPM

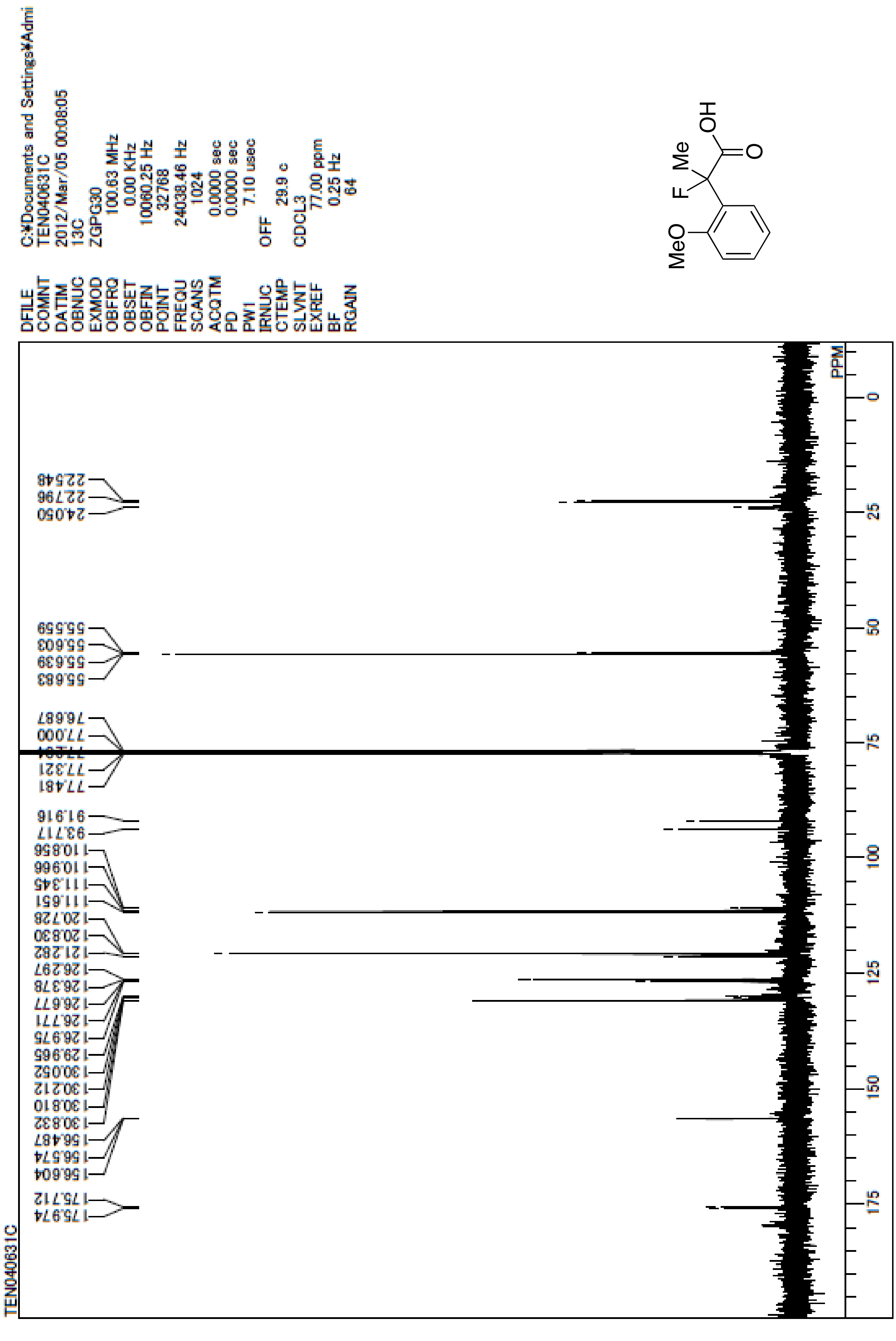

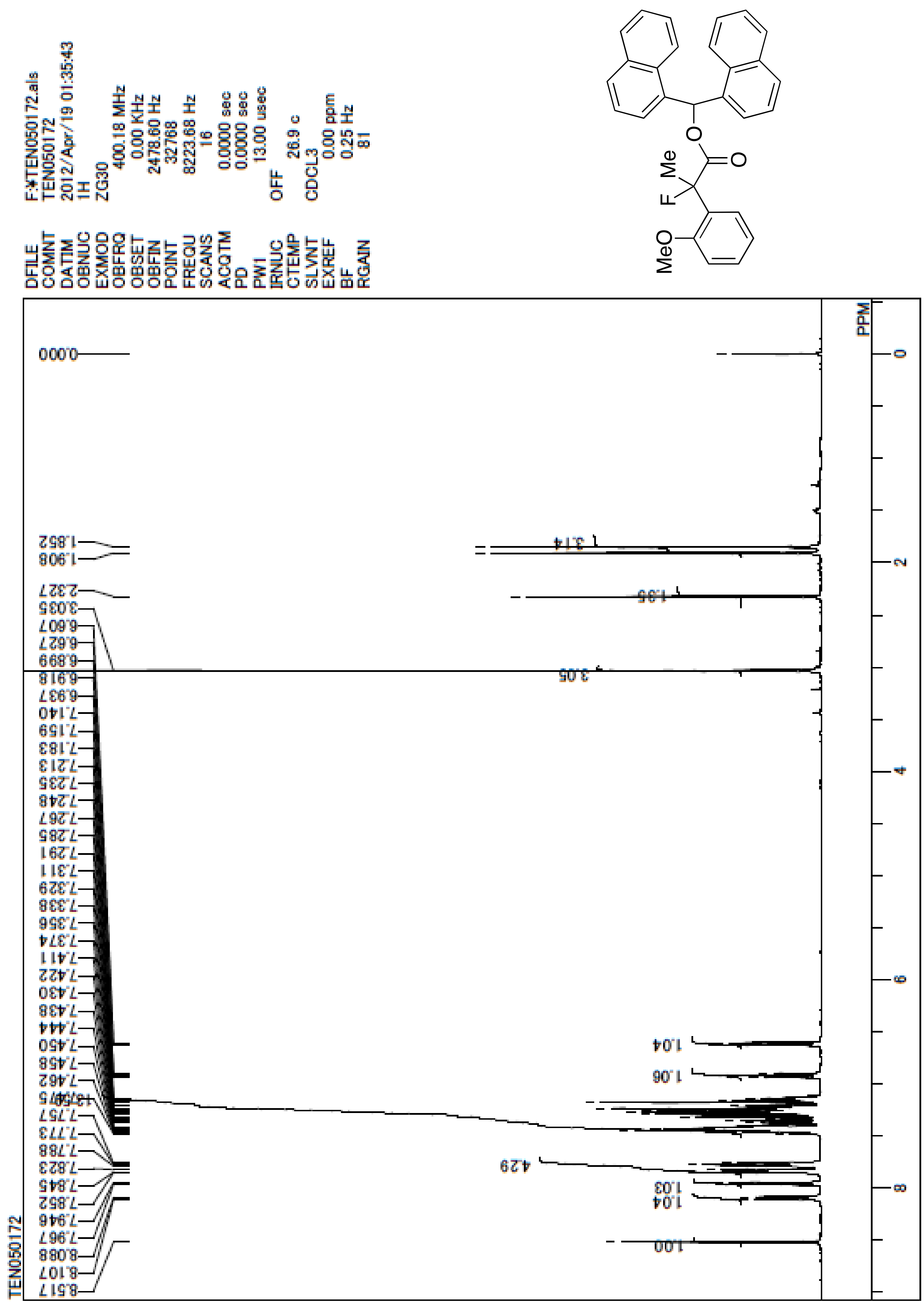

DFILE F:¥TEN050172.als
COMNT TEN050172
DATIM 2012/Apr/19 01:35:43
OBNUC 1H
EXMOD ZG30
OBFRQ 400.18 MHz
OBSET 0.00 KHz
OBFIN 2478.60 Hz
POINT 32768
FREQU 8223.68 Hz
SCANS 16
ACQTM 0.0000 sec
PD 0.0000 sec
PW1 13.00 usec
IRNUC OFF
CTEMP 26.9 c
SLVNT CDCL3
EXREF 0.00 ppm
BF 0.25 Hz
RGAIN 81
MeO
F
Me
O
O
PPM
TEN050172

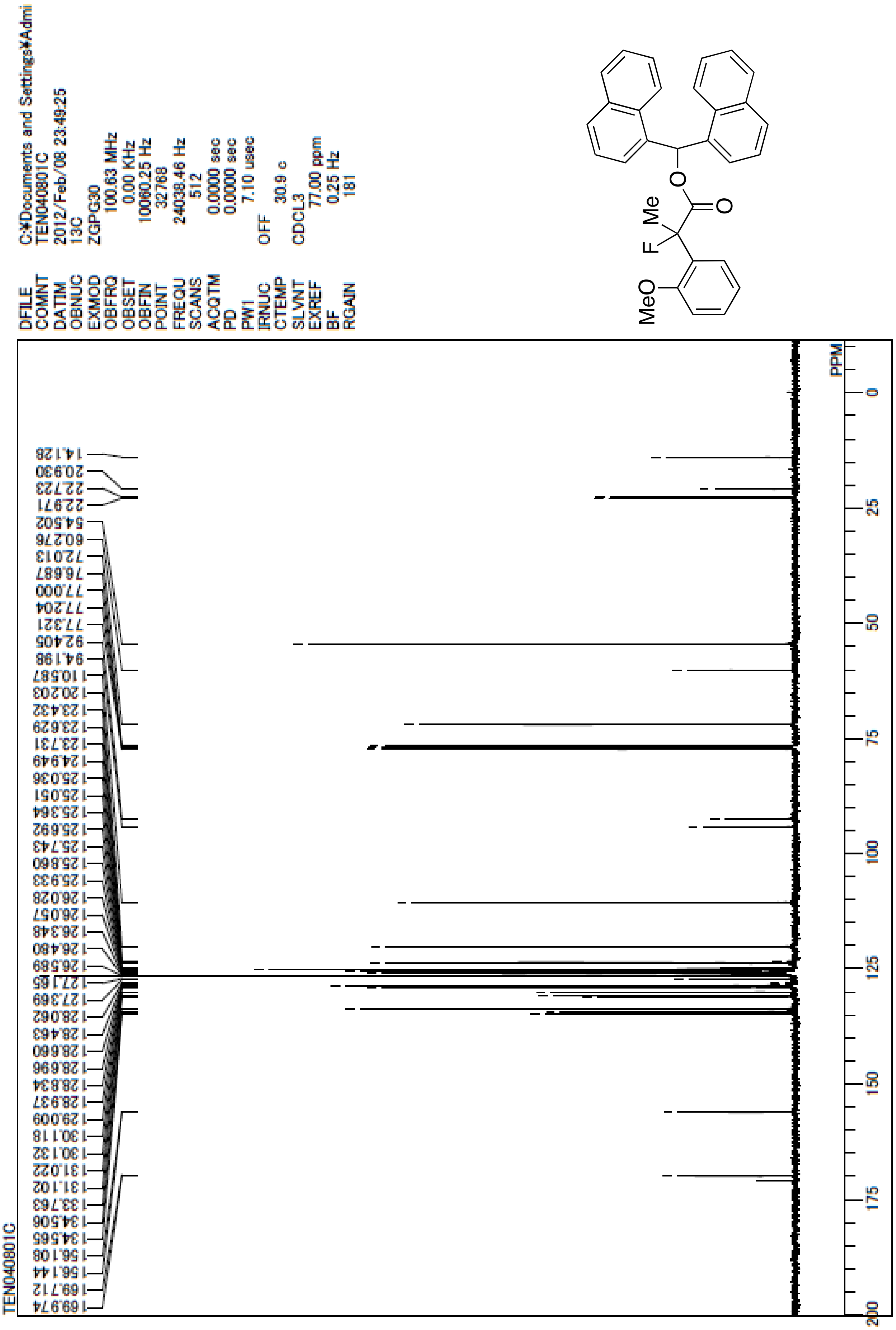
DFILE C:¥Documents and Settings¥Admi
COMNT TEN040801C
DATIM 2012/Feb/08 23:49:25
OBNUC 13C
EXMOD ZGPG30
OBFRQ 100.63 MHz
OBSET 0.00 KHz
OBFIN 10060.25 Hz
POINT 32768
FREQU 24038.46 Hz
SCANS 512
ACQTM 0.0000 sec
PD 0.0000 sec
PW1 7.10 usec
IRNUC OFF
CTEMP 30.9 c
SLVNT CDCL3
EXREF 77.00 ppm
BF 0.25 Hz
RGAIN 181
MeO
F
Me
O
O
TEN040801C
14.128
20.930
22.723
22.971
54.502
60.276
72.013
76.687
77.000
77.204
77.321
92.405
94.198
110.587
120.203
123.432
123.629
123.731
124.949
125.036
125.051
125.364
125.692
125.743
125.860
125.933
126.028
126.057
126.348
126.480
126.569
127.165
127.369
128.062
128.463
128.660
128.696
128.834
128.937
129.009
130.118
130.132
131.022
131.102
133.763
134.506
134.565
156.108
156.144
169.712
169.974
PPM
0
25
50
75
100
125
150
175
200

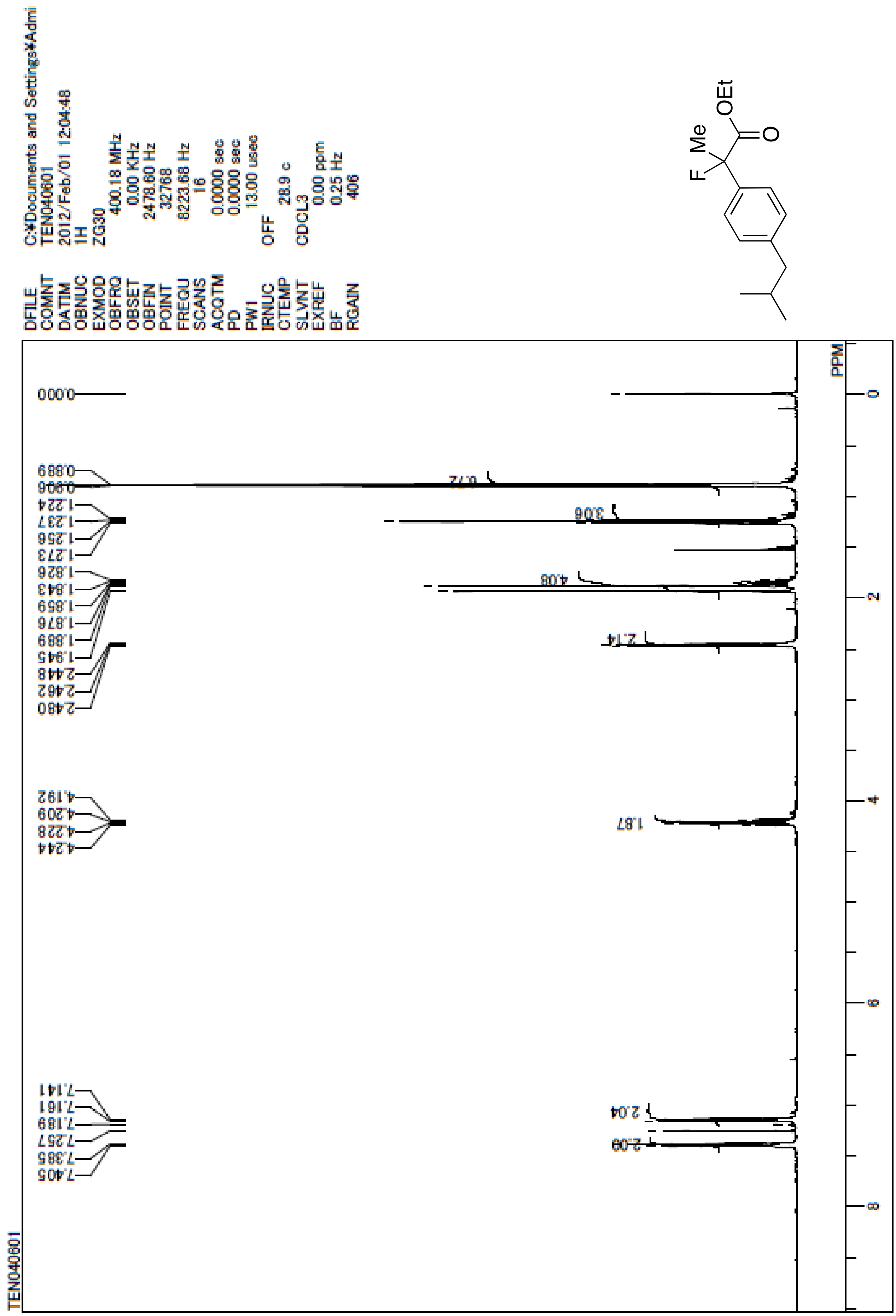

DFILE C:¥Documents and Settings¥Admi
COMNT TEN040601
DATIM 2012/Feb/01 12:04:48
OBNUC 1H
EXMOD ZG30
OBFRQ 400.18 MHz
OBSET 0.00 KHz
OBFIN 2478.60 Hz
POINT 32768
FREQU 8223.68 Hz
SCANS 16
ACQTM 0.0000 sec
PD 0.0000 sec
PW1 13.00 usec
IRNUC OFF
CTEMP 28.9 c
SLVNT CDCL3
EXREF 0.00 ppm
BF 0.25 Hz
RGAIN 406
F
Me
OEt
O
TEN040601
PPM

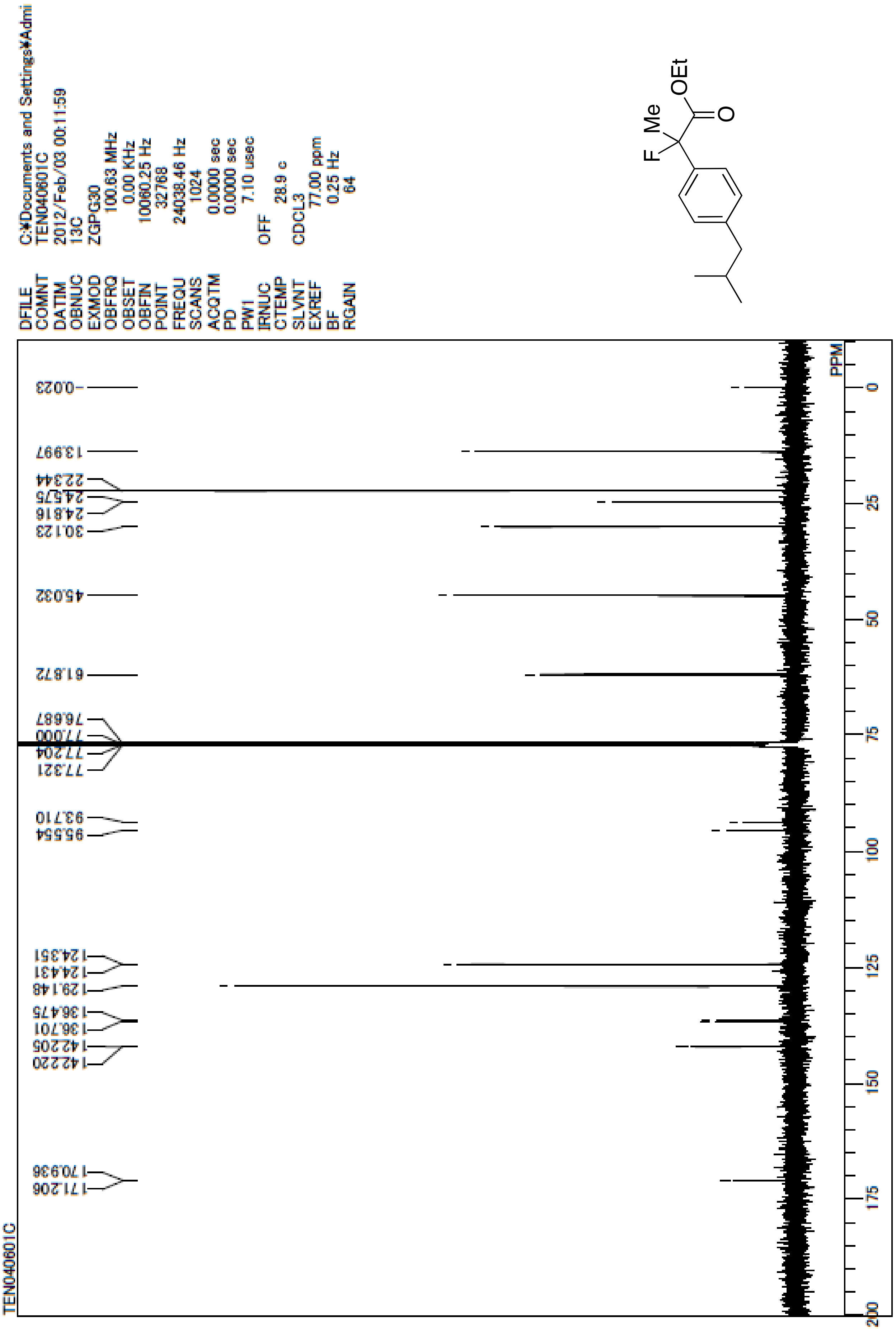
DFILE C:¥Documents and Settings¥Admi
COMNT TEN040601C
DATIM 2012/Feb/03 00:11:59
OBNUC 13C
EXMOD ZGPG30
OBFRQ 100.63 MHz
OBSET 0.00 KHz
OBFIN 10060.25 Hz
POINT 32768
FREQU 24038.46 Hz
SCANS 1024
ACQTM 0.0000 sec
PD 0.0000 sec
PW1 7.10 usec
IRNUC OFF
CTEMP 28.9 c
SLVNT CDCL3
EXREF 77.00 ppm
BF 0.25 Hz
RGAIN 64
F
Me
OEt
O
TEN040601C
PPM

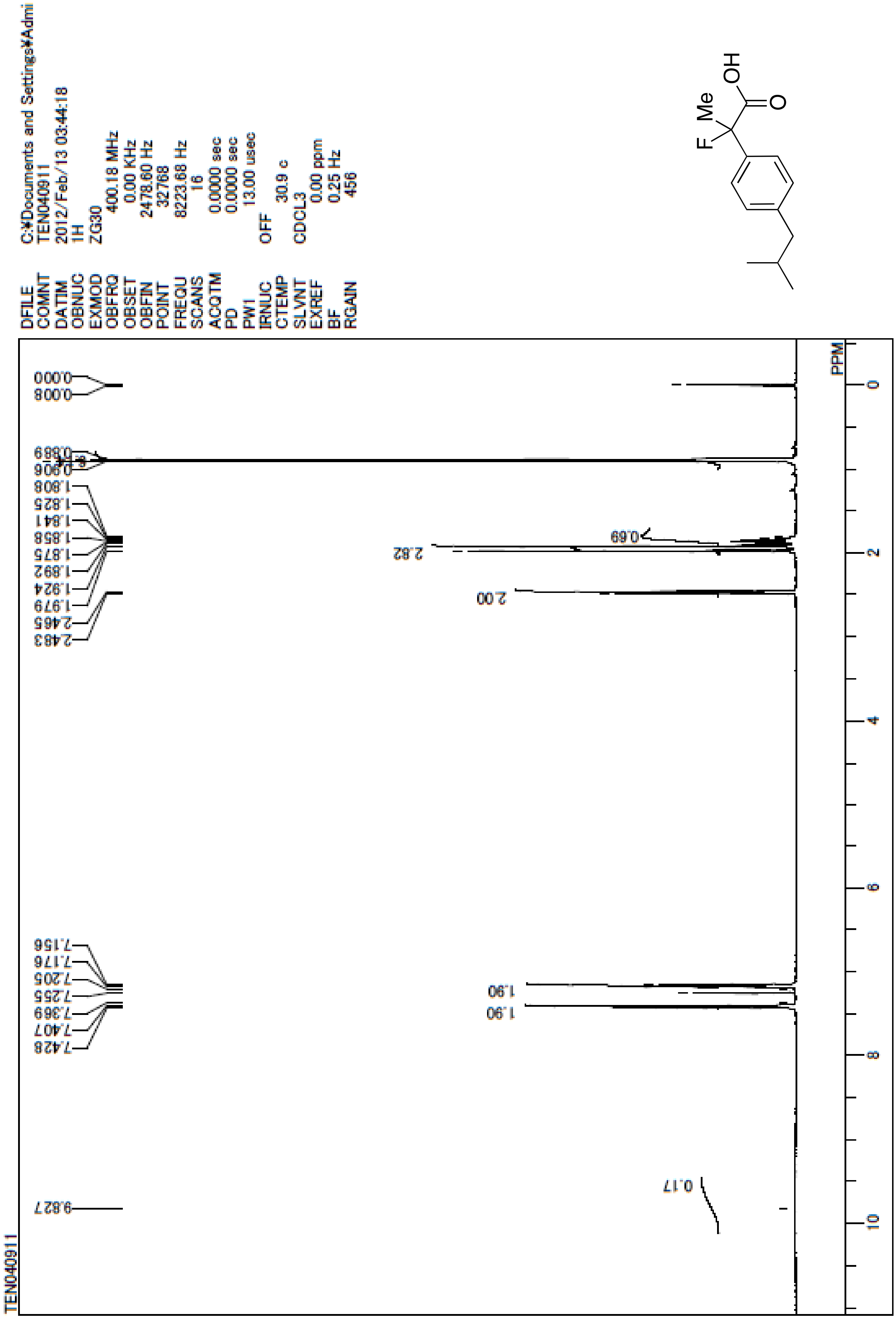
DFILE C:¥Documents and Settings¥Admi
COMNT TEN040911
DATIM 2012/Feb/13 03:44:18
OBNUC 1H
EXMOD ZG30
OBFRQ 400.18 MHz
OBSET 0.00 KHz
OBFIN 2478.60 Hz
POINT 32768
FREQU 8223.68 Hz
SCANS 16
ACQTM 0.0000 sec
PD 0.0000 sec
PW1 13.00 usec
IRNUC OFF
CTEMP 30.9 c
SLVNT CDCL3
EXREF 0.00 ppm
BF 0.25 Hz
RGAIN 456
F
Me
OH
O
TEN040911
PPM

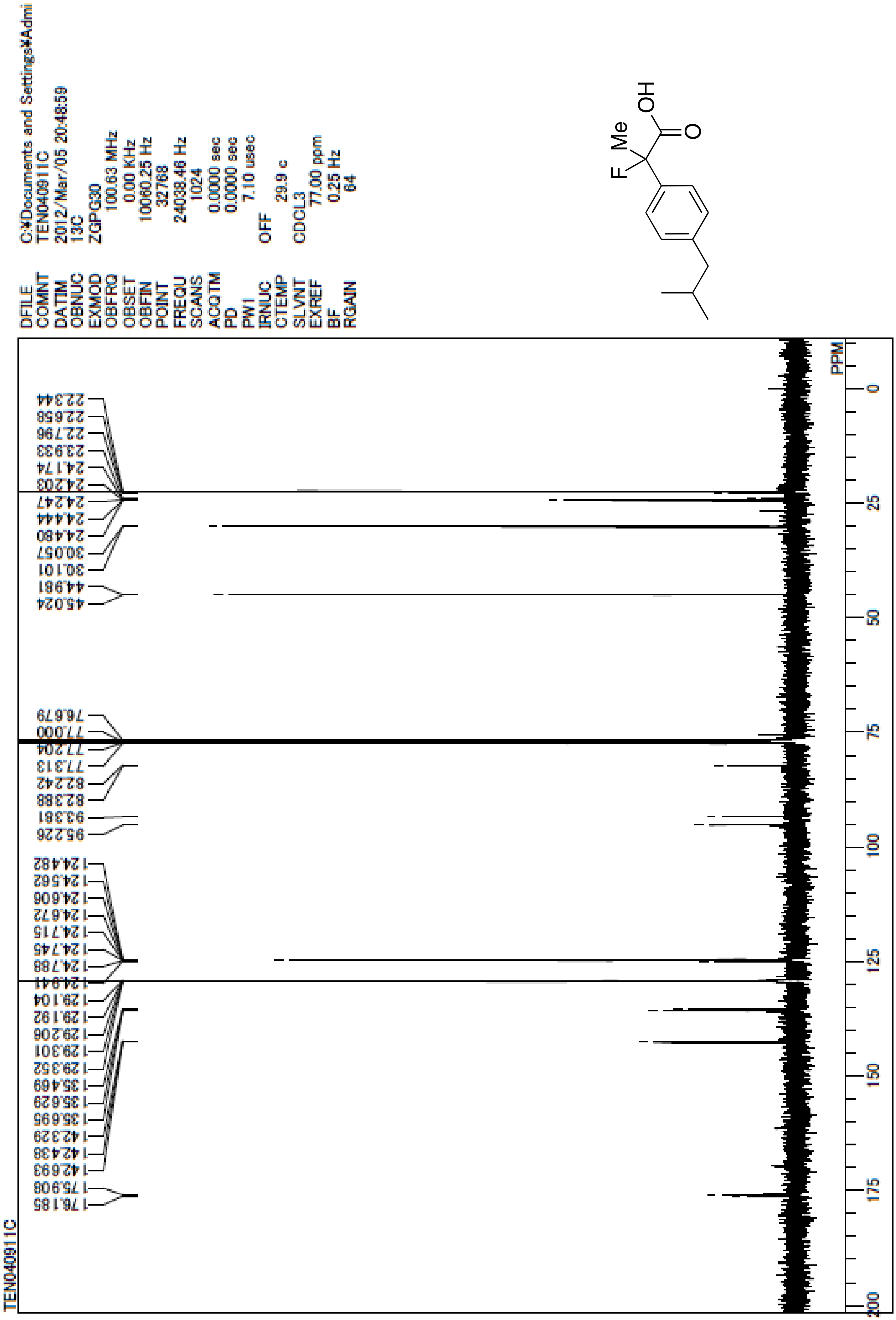
DFILE C:¥Documents and Settings¥Admi
COMNT TEN040911C
DATIM 2012/Mar/05 20:48:59
OBNUC 13C
EXMOD ZGPG30
OBFRQ 100.63 MHz
OBSET 0.00 KHz
OBFIN 10060.25 Hz
POINT 32768
FREQU 24038.46 Hz
SCANS 1024
ACQTM 0.0000 sec
PD 0.0000 sec
PW1 7.10 usec
IRNUC OFF
CTEMP 29.9 c
SLVNT CDCL3
EXREF 77.00 ppm
BF 0.25 Hz
RGAIN 64
F
Me
OH
O
TEN040911C
22.344
22.658
22.796
23.933
24.174
24.203
24.247
24.444
24.480
30.057
30.101
44.981
45.024
76.679
77.000
77.204
77.313
82.242
82.388
93.381
95.226
124.482
124.562
124.606
124.672
124.715
124.745
124.788
124.841
129.104
129.192
129.206
129.301
129.352
135.469
135.629
135.695
142.329
142.438
142.693
175.908
176.185
PPM
0
25
50
75
100
125
150
175
200

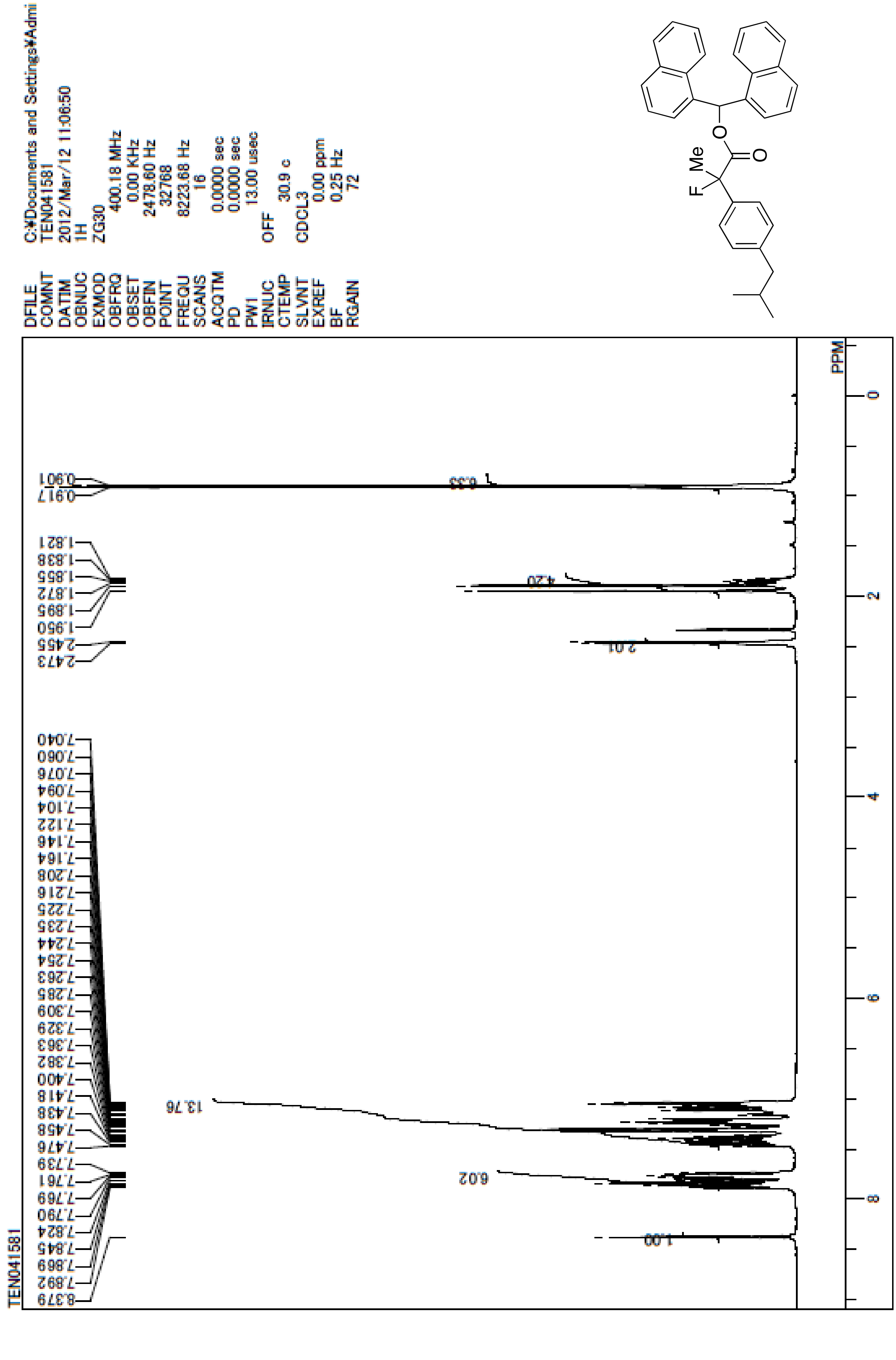

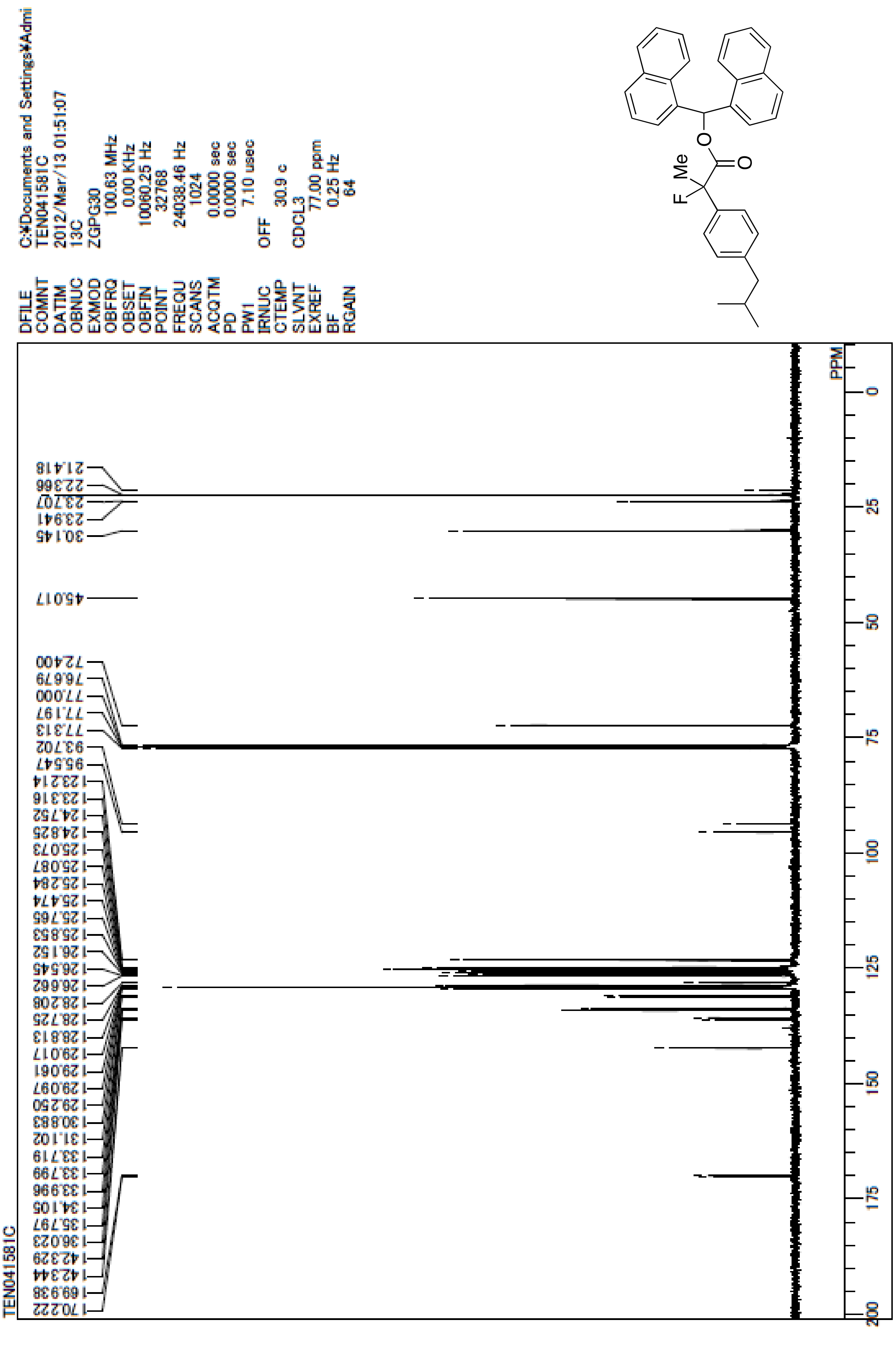
DFILE C:¥Documents and Settings¥Admi
COMNT TEN041581C
DATIM 2012/Mar/13 01:51:07
OBNUC 13C
EXMOD ZGPG30
OBFRQ 100.63 MHz
OBSET 0.00 KHz
OBFIN 10060.25 Hz
POINT 32768
FREQU 24038.46 Hz
SCANS 1024
ACQTM 0.0000 sec
PD 0.0000 sec
PW1 7.10 usec
IRNUC OFF
CTEMP 30.9 c
SLVNT CDCL3
EXREF 77.00 ppm
BF 0.25 Hz
RGAIN 64
F
Me
O
O
TEN041581C
21.418
22.366
23.707
23.941
30.145
45.017
72.400
76.679
77.000
77.197
77.313
93.702
95.547
123.214
123.316
124.752
124.825
125.073
125.087
125.284
125.474
125.765
125.853
126.152
126.545
126.662
128.206
128.725
128.813
129.017
129.061
129.097
129.250
130.883
131.102
133.719
133.799
133.996
134.105
135.797
136.023
142.329
142.344
169.938
170.222
PPM
0
25
50
75
100
125
150
175
200
